# Tetratellura[36]octaphyrin(1.1.1.1.1.1.1.1) Metalation: From Dynamic Behavior to Rigid Chiral Figure-of-Eight Molecule; Activation of the C–Te Bond by Ruthenium

**DOI:** 10.1021/acs.inorgchem.4c03506

**Published:** 2024-11-06

**Authors:** Paulina Krzyszowska, Emilia Ganczar, Piotr J. Chmielewski, Ewa Pacholska-Dudziak

**Affiliations:** Department of Chemistry, University of Wroclaw, ul. Joliot-Curie 14, 50-383 Wroclaw, Poland

## Abstract

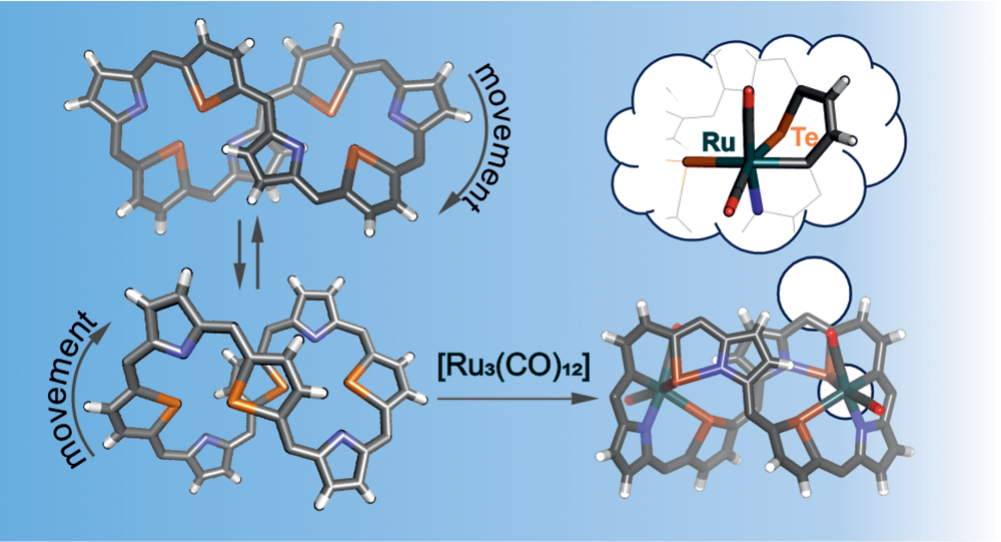

The large expanded telluraporphyrin, tetratellura[36]octaphyrin(1.1.1.1.1.1.1.1), was synthesized in 7% yield by acid-catalyzed condensation of pyrrole with 2,5-bis(phenylhydroxymethyl)tellurophene and subsequent oxidation. The macrocycle acquires the chiral figure-eight conformation with two alternative spatial arrangements of the heterocyclic rings. The molecule exhibits dynamic behavior in solution, which was studied by means of ^1^H NMR spectroscopy. The reaction with chlorine led to a reversible oxidative addition at two distant tellurium atoms, which altered the preferred conformations. Metalation of the tetratellura[36]octaphyrin with triruthenium dodecacarbonyl selectively activated two of the eight Te–C bonds, resulting in the formation of an organometallic diruthenium compound. During the reaction, two tellurophene rings were converted to 1-ruthena-2-telluracyclohexadiene units containing octahedral ruthenium(II) centers. The rigid structure of this chiral complex allowed the separation of enantiomers.

## Abstract 2

A chiral figure-eight expanded telluraporphyrin is reported, namely tetratellura[36]octaphyrin(1.1.1.1.1.1.1.1). The molecule exhibits high flexibility in solution and forms two mutually transforming conformers, one with tellurophene rings at the figure-eight ribbon-crossing and the other with pyrrole rings at this position. Metalation of the tetratellura[36]octaphyrin with triruthenium dodecacarbonyl resulted in the formation of a rigid organometallic diruthenium(II) compound incorporating 1-ruthena-2-telluracyclohexadiene units.

## Introduction

**Chart 1:**
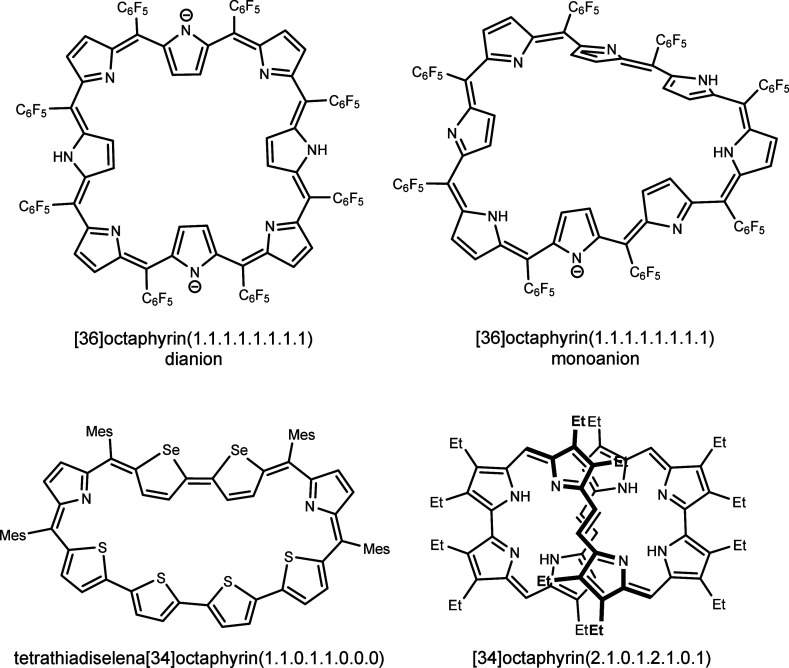
Examples of Octaphyrin Scaffolds^12−14^a

Octaphyrins are expanded porphyrins that exhibit several intriguing features, such as structural diversity and conformational flexibility in solution, including the π-system topology switch and electronic state variability. Their large central cavity is capable of multication and anion-binding.^1−4^ The most typical building blocks, the pyrrole rings, are frequently replaced by geometrically and electronically different pentacyclic rings, like furan, thiophene, and selenophene, giving more degrees of freedom to the octaphyrin properties.^5−7^ Thus, the symmetry and shape of octaphyrins depend on the identity of the eight constituent units forming the macrocycle and the number and distribution of *meso* links.^8−10^ These factors determine the so-called free curvature of the macrocyclic ring, a geometrical descriptor of an angular strain.^9^ This strain is reduced by several conformational changes, like subunit inversions and π-surface twisting, leading to a variety of octaphyrins shapes, from planar, including square,^11,12^ or crescent-shaped,^13^ to figure-eights (360°-twisted band)^14,15^ and several distorted 3D geometries (Chart 1). The π-system topology and the π-electron count in the conjugation path, which ranges from 30 to 40 for various octaphyrins, may result in Hückel aromatic, Hückel antiaromatic, or Möbius aromatic structures, reflected in diatropicity or paratropicity in their ^1^H NMR spectra.^9,16^ Stabilization of exotic Möbius-band topology (180°-twisted band) structures was achieved by protonation, deprotonation, or palladium(II) binding in the macrocycle.^12,17,18^ Also a bis-zinc(II) complex of an internally bridged tetrabromo[36]octaphyrin has been shown to be a 36-π-electron Möbius aromatic species.^19^ A rare example of a stable radical cation is represented by a bis-silicon [37]octaphyrin, exhibiting a figure-of-eight structure, as characterized in the solid state.^16^ The figure-eight, which is a chiral doubly twisted conformation, was first documented in the seminal work on [32]octaphyrin(1.0.1.0.1.0.1.0).^20^ Such a geometry proved relatively common and was observed for different peripheral substitution patterns and several *meso*-bridging patterns, like [34]octaphyrin(1.1.1.0.1.1.1.0),^14^ [36]octaphyrin(2.1.0.1.2.1.0.1),^21^ the classical octaaryl[36]octaphyrin(1.1.1.1.1.1.1.1),^22^ and its two tetrathia-analogues.^15^ In the case of [32]octaphyrin(1.0.1.0.1.0.1.0), the figure-eight structure was stabilized by π···π and C–H···π interactions, as well as a set of N–H···N hydrogen bonds, leading to conformational stability, allowing the enantiomers’ separation.^23,24^ An unusual example of the optical resolution was achieved for [38]octaphyrin(1.1.1.1.1.1.1.1) due to a quadruple inner *N*-methylation leading to relatively high inversion barrier.^25^ An introduction of larger π-conjugated building blocks, such as the phenanthrene moieties in diphenanthrioctaphyrin(1.1.1.0.1.1.1.0),^26^ allowed the formation of two distinct “locked conformations” that readily interconvert in the presence of the hydrogen bond acceptors. Such conformationally locked expanded porphyrinoids may represent promising functional materials with unique chiroptical properties.^27^ On the other hand, the dynamic properties on several levels of motion, depending on the specific *meso* link arrangement and the peripheral substitution pattern, are of interest and have been studied for figure-eight octaphyrins. Thus, for tetrathia[36]octaphyrin(1.1.1.1.1.1.1), the conveyor-belt motion of the entire macrocyclic ring, which does not require a racemization step, was proposed on the basis of detailed NMR studies.^15^ The mechanism of enantiomers’ interconversion was examined for a series of *meso*-tetraphenyl[32]octaphyrins(1.0.1.0.1.0.1.0) with different alkyl substituents on 2,2′-bipyrrole components.^24^ Based on the spectroscopic observation of diastereotopic CH_2_ protons as a symmetry probe, an inversion was proposed to occur by a stretching–compressing mechanism that does not involve planarization. In cases where the interconversion mechanism would involve energetically requiring unwinding and rewinding of the figure-eight, the octaphyrins are configurationally stable.^9,14,21^

a A number in square brackets denotes the π-electrons count in the conjugated pathway; numbers in round brackets indicate the lengths of the bridges connecting the heterocyclic units.

Octaphyrins, possessing wide cavities, are suitable for two or more metal or nonmetal cations binding. Metalation of figure-eight octaphyrins may fix the macrocycle shape or may be accompanied by macrocyclic skeleton conformation changes or side reactions. Stable figure-eight bis(palladium) and bis(nickel) complexes of [32]octaphyrin(1.0.1.0.1.0.1.0) were obtained in a metal-templated synthesis.^28^ Metalation of [36]octaphyrin(2.1.0.1.2.1.0.1) by metal sources with optically active carboxylate gave enantioselectively stereochemically stable bimetallic complexes.^21,29^ The binding of two phosphorus(V) centers, through NNC and NNNCC sites, stabilizes the highly reduced 40-π-electron-conjugated system, a so-called expanded isophlorin.^30^ The figure-eight-shaped [36]octaphyrin unwinding and pyrrole inversion were triggered by a single iridium(I) binding.^31^ Metalation frequently triggered spectacular and unexpected skeletal rearrangements,^32^ such as the formation of dinuclear nickel spirodicorrole from the dioxo-derivative of octaphyrin(1.1.1.0.1.1.1.0);^33^ [36]octaphyrin(1.1.1.1.1.1.1.1) splitting upon metalation with copper, providing a rare example of “molecular mitosis”;^34^ and internally bridged octaphyrin(1.1.1.1.1.1.1.1) with zinc(II) in oxidative conditions, transformed into porphyrin dimers.^35^ Interestingly, the copper(II) insertion to perfluorinated [36]octaphyrin(1.1.1.1.1.1.1.1) is accompanied by hydrolytic cleavage of the pyrrolic ring to a keto-imine.^36^

## Results

**Scheme 1:**
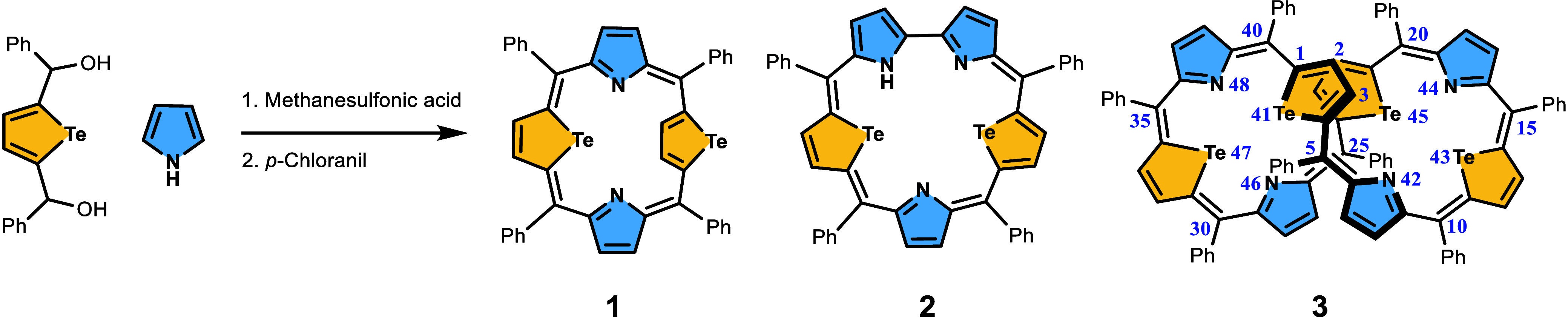
One-Pot Synthesis of a Variety of *meso*-Phenyl-Substituted Telluraporphyrinoids

Tellurophene-containing porphyrinoids are notable for their atypical geometry, dynamic properties, and distinctive reactivity toward transition metals, resulting from the reactivity of the Te–C bond. A limited number of expanded telluraporphyrins have been documented in the literature.^37,38^ As an effort to expand the group of expanded telluraporphyrins, here we report the synthesis of an octaphyrin(1.1.1.1.1.1.1.1) heteroanalogue, containing four tellurophene units, namely, *meso*-octaphenyl-41,43,45,47-tetratellura[36]octaphyrin(1.1.1.1.1.1.1.1), **3** (Scheme 1). The product was obtained in a one-pot acid-catalyzed condensation of pyrrole and 2,5-bis(phenylhydroxymethyl)tellurophene in a 1:1 molar ratio, followed by oxidation with chloranil. Macrocyclization leading to the desired tetratellura[36]octaphyrin was accompanied by smaller rings formation, accessible within the same synthetic protocol, in particular the main reaction product, 21,23-ditelluraporphyrin, **1**.^37,38^ Another minor product, already reported in the literature, 26,28-ditellurasapphyrin, **2**, was formed in important amounts with methanesulfonic acid as catalyst and in the presence of excess pyrrole, with a yield strongly dependent on the substrates molar ratio.^38,39^ In contrast, the tetratellura[36]octaphyrin, **3**, yield was maximized (7%) for the equimolar substrate mixture, while the choice of the acid was not crucial, and both tested acids, methanesulfonic and boron trifluoride etherate, gave similar results. The tetratellura[36]octaphyrin was also formed in the reaction between 2,5-bis(phenylhydroxymethyl)tellurophene and telluratripyrrane, according to the [3 + 1 + 3 + 1] scheme.^38^ For each method, the complex postsynthetic mixture, containing tetraphenyl-21,23-ditelluraporphyrin, **1**, tetraphenyl-26,28-ditellurasapphyrin, **2**, 41,43,45,47-tetratellura[36]octaphyrin **3**, and other unidentified products, required deliberate separation. Standard basic alumina chromatography allowed for the separation of two green porphyrinoids, **1** and **3**, from the mixture, which subsequently were subjected to size-exclusion chromatography.

**Scheme 2:**
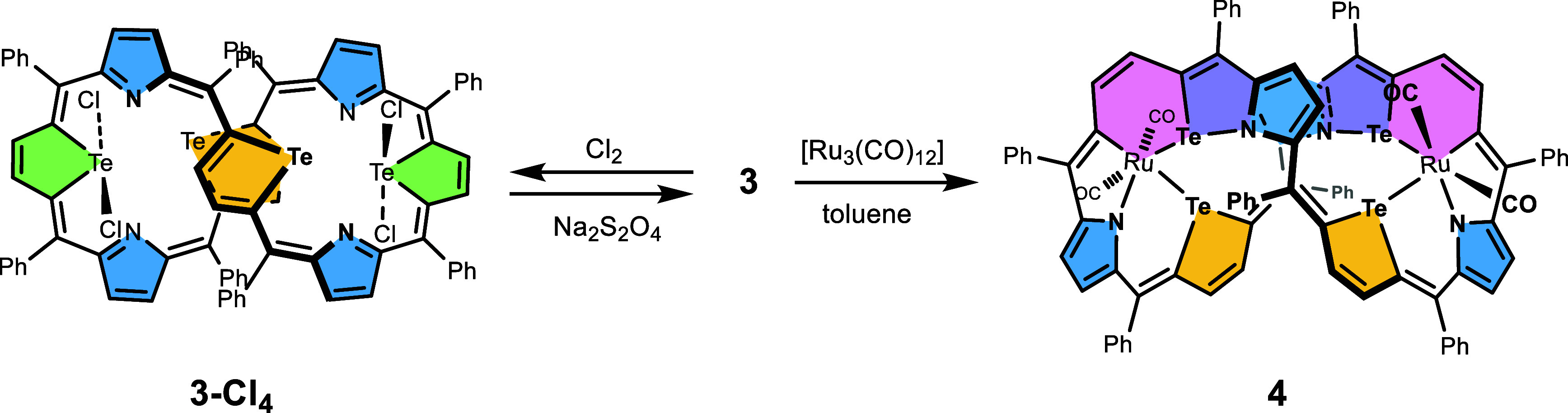
Reactivity of Tetratellura[36]octaphyrin **3**

The tetratelluraoctaphyrin **3** forms a stable in air and light sea-green solution, distinctly different in color from the grass-green ditelluraporphyrin **1**. In contrast to **1**, macrocycle **3** slowly decomposes under the influence of excess hydrochloric acid or 2,3-dichloro-5,6-dicyano-1,4-benzoquinone (DDQ). The macrocycle **3** underwent oxidative addition with Cl_2_ to give a moderately stable 41,41,45,45-tetrachloro-41,43,45,47-tetratellura[36]octaphyrin, **3-Cl**_**4**_, chlorinated at two distant tellurium atoms (Scheme 2). The single product was formed quantitatively if chlorine was added slowly in small portions under ^1^H NMR spectroscopy control, while the use of an excess of chorine yielded **3-Cl**_**4**_ contaminated with a mixture of unstable products. Upon standing in solution, tetrachloroderivative **3-Cl**_**4**_ underwent a gradual transformation into a mixture of macrocycle **3** and unidentified products. Its chromatographic workup led to the decomposition of the macrocyclic species. A well-controlled reduction of **3-Cl**_**4**_ with sodium dithionite (Na_2_S_2_O_4_/H_2_O) allowed for a practically quantitative recovery of **3**, while the use of zinc amalgam gave a less clean product. The chlorination of **3**, with the formation of tellurium(IV)-containing **3-Cl**_**4**_, proceeded easily, unlike 21-telluraporphyrin, which under a straightforward halogenation gave only monohalogenated species,^40^ while 21,21-dihaloderivatives could only be obtained from 21-oxo-21-telluraporphyrin with HCl.^41^

The 36-π-electron system of **3** was also expected to exhibit redox reactivity. The cyclic voltammetry shows an irreversible oxidation at 0.31 V and an irreversible reduction at −0.92 V (vs Fc^+^/Fc; Figure S11 in the Supporting Information). However, attempts to oxidize the π-electron system of tetratellura[36]octaphyrin and/or to induce skeletal fusion reactions, which are relatively common among expanded porphyrinoids, were not successful. The reaction with FeCl_3_ led to **3-Cl**_**4**_, while other typical reagents (DDQ, chloranil, and diphenyliodonium diacetate (PIDA))^42,43^ resulted in the macrocycle decomposition to tar products. The reduction of the 4*n*-π-electron system to a related 4*n* + 2-π-electron potentially aromatic structure, known for the sulfur congener, tetrathia[38]octaphyrin,^15^ failed for **3** (NaBH_4_, Na_2_S_2_O_4_) as well. Nevertheless, a four-electron reduction was observed concomitant with the tetratellura[36]octaphyrin metalation. Thus, the metalation of **3** with an excess of triruthenium dodecacarbonyl resulted in the insertion of ruthenium atoms into two tellurium–carbon bonds, leading to the formation of symmetrical organometallic dinuclear ruthenium(II) product **4** (Scheme 2). The insertion proceeds via an oxidative addition of the Te–C bond to the low-valent ruthenium(0), with the formation of ruthenium(II) species. The transformation of two tellurophene units into 1-ruthena-2-telluracyclohexadiene rings, RuTeC_4_, was accompanied by the fusion of these six-membered rings with adjacent pyrrole rings through the formation of a Te–N bond and a formal four-electron macrocycle reduction to a 40-π-electron circuit. The use of a lower than optimal quantity of [Ru_3_(CO)_12_] resulted in a decreased conversion of the substrate, yet the formation of a diruthenium complex was observed exclusively, indicating that the second ruthenium insertion was facilitated. The excess of the metal source consistently yielded **4**, however, and traces of a compound containing three ruthenium atoms and six carbonyls were detected by mass spectrometry. What is more, solely one of several conceivable isomers of **4** was observed; thus, taking under consideration its relatively high yield, 70%, the reaction exhibits high selectivity. The product, **4**, with *C*_2_ symmetry exhibited chirality, and the enantiomers were stable enough to permit their separation. The transformation of two heterocyclic building blocks into six-membered units, 1-ruthena-2-telluracyclohexadiene, represents a very rare metal-binding mode by porphyrinoids. A similar RuTeC_4_ ring has been described for the reaction of benzo[*b*]tellurophene with triruthenium dodecacarbonyl.^44^ In the field of porphyrinoid chemistry, a rupture of a pyrrole ring accompanied by an insertion of a ruthenium atom into a C–N bond was observed for a *N,N’*-vinyl-bridged porphyrin under the influence of triruthenium dodecacarbonyl.^45,46^ The six-membered 1-rhoda-2-telluracyclohexadiene unit, RhTeC_4_, integrated within the porphyrin frame was spectroscopically detected in a transient species during the transformation of 21,23-ditelluraporphyrin into 21-rhoda-23-telluraporphyrin, which is a porphyrin comprising a rhodacyclopentadiene unit.^47^ The complex **4** did not undergo further transformation into a metallacyclopentadiene-containing porphyrinoid.

**Figure 1:**
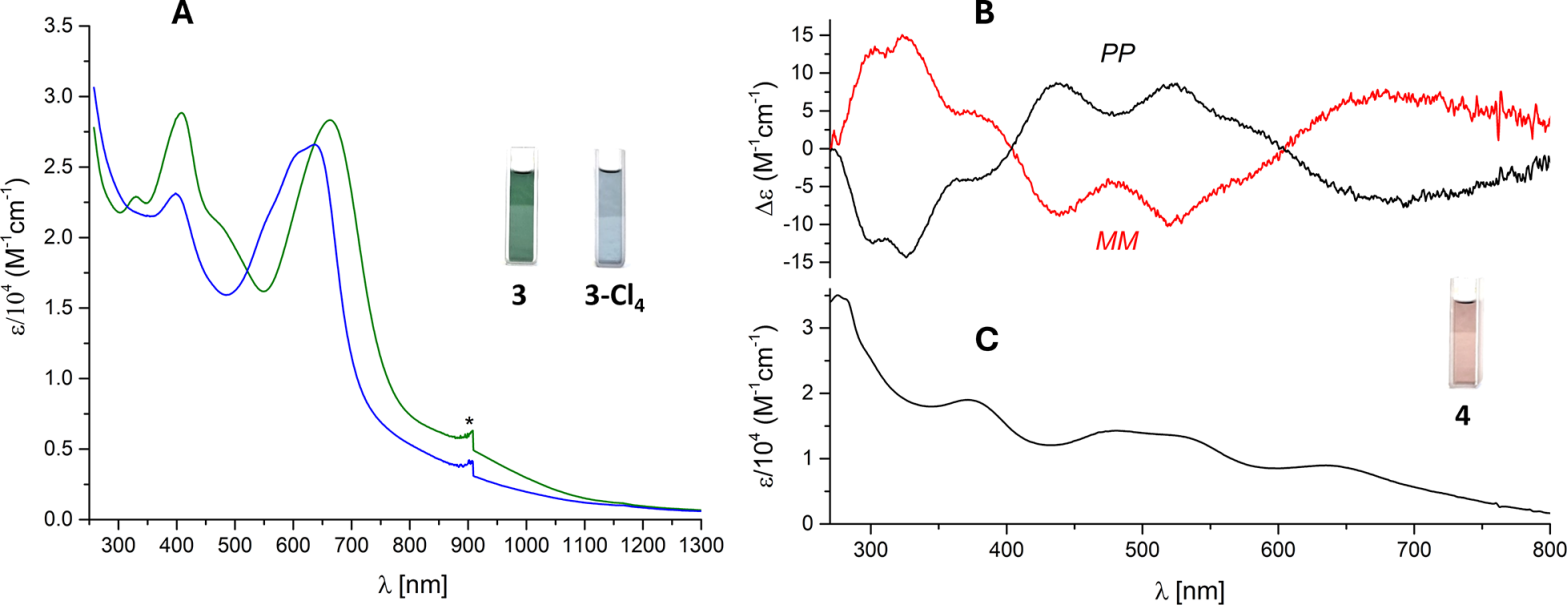
(A) UV–vis electronic spectra (in CH_2_Cl_2_) of **3** (green line) and **3-Cl**_**4**_ (blue line); * = artifacts. (B) Circular dichroism of **4** (CH_2_Cl_2_/hexane, 3:7). (C) UV–vis electronic spectrum of **4** (CH_2_Cl_2_).

The electronic spectra of compounds **3** and **3-Cl**_**4**_ exhibit two broad bands of similar intensity at 400 and 410 nm, respectively, and at 600 and 650 nm (Figure 1A). The green (**3**) and blue (**3-Cl**_**4**_) colors of these compounds’ solutions reflect the absorption in these regions. In contrast to that of porphyrins and aromatic expanded porphyrins, an intense Soret band is absent. The electronic spectrum of the pink-brown ruthenium derivative, **4** (Figure 1C), devoid of distinct features, shows five broad overlapping bands spread from 295 to 647 nm, with gradually descending intensities.

All of the presented molecules are chiral, but only ruthenium derivative **4** possesses a structure rigid enough to allow the separation of the racemic mixture into enantiomers. The separation was performed by HPLC equipped with a chiral stationary phase. The optical activity of both collected fractions was confirmed by the measurement of circular dichroism spectra (Figure 1B). The absolute configurations of the enantiomers were assigned by a comparison of the experimental and calculated by time-dependent density functional theory (TDDFT) spectrum for the *P,P* enantiomer on the basis of X-ray crystallographic data (Figure S23 in the Supporting Information). The enantiomers were found to be relatively stable in a toluene solution at room temperature, with racemization occurring completely within 9 days.

## X-ray Structures

**Figure 2:**
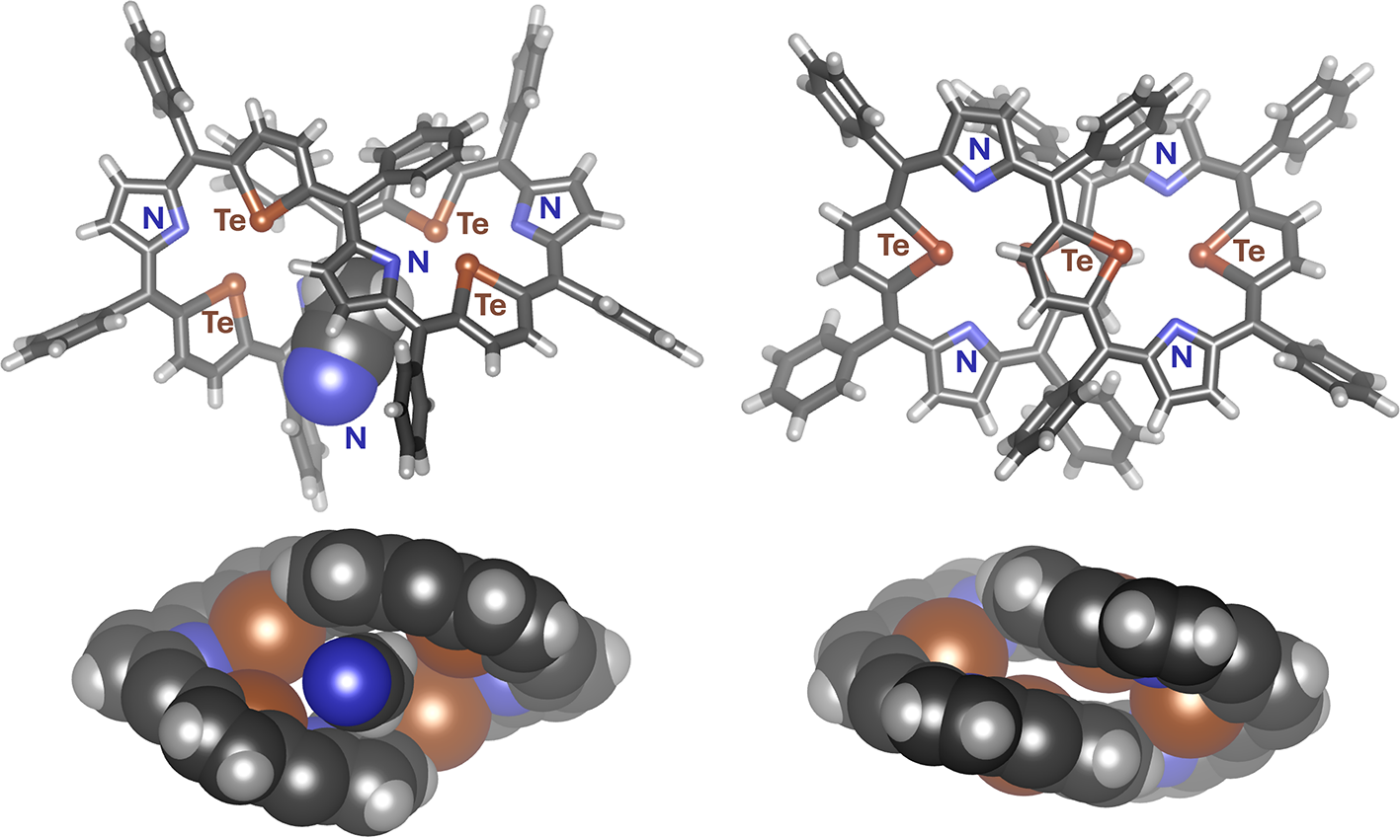
X-ray molecular structures of CH_3_CN·**3-NN** (left) and **3-TeTe** (right). The *M,M* enantiomer is presented; in the bottom projection (CPK) phenyl rings are omitted for clarity.

The crystallization of the macrocycle **3** by slow diffusion of acetonitrile into the chloroform solution of the tetratellura[36]octaphyrin gave two differently shaped crystals, easily crystallizing plates and slowly crystallizing needles. The use of *n*-hexane as the precipitating solvent yielded X-ray quality needles and only poor-quality plates unsuitable for X-ray studies, clearly showing the importance of solvents in crystal packing. The two crystal habits disclosed various molecular structures of the tetratellura[36]octaphyrin, although both presented twisted figure-eight-shaped molecules. Both forms exhibited near *C*_2_ symmetry and chirality but crystallized in achiral space groups as racemic mixtures. The conformation revealed in the plates (*P*2_1_/*c*), grown from an acetonitrile/chloroform system, had two pyrrole rings on the three-dimensional figure-eight central crossing and hereafter is to be denoted as **3-NN** (Figure 2 and Figure S24 in the Supporting Information). For each molecule of **3-NN** there were five molecules of acetonitrile in the crystal structure, occupying 2311 Å^3^, that is, 27% of the unit cell volume.^48^ In comparison, the conformation found in needles (*C*2/*c*) obtained with *n*-hexane/chloroform exhibited two tellurophene rings in this position and is to be referred to as **3-TeTe** (Figure 2 and Figure S25 in the Supporting Information). In this crystal structure, the solvent, one chloroform molecule per one octaphyrin molecule, occupies only 10% of the unit cell. The central pentacyclic rings in **3-TeTe** are significantly closer to each other (tellurophene centroids distance, “*the intersection pitch*”: 4.66 Å) than two pyrrole rings on the ribbon-crossing in **3-NN**, with centroids separated by 6.70 Å. The gap in the latter form is large enough to allow for intercalation of an acetonitrile molecule, oriented with the CH_3_ group toward the cavity, bonding to the two central pyrrole rings via three C–H···π hydrogen bonds^49^ (centroid···H distances: 2.57, 2.90, and 3.25 Å). The C–H···π interactions of CH_3_ groups of acetonitrile or in other small molecules or cations with pyrrole units are common in porphyrinoid crystal structures, e.g., they play a role in additional stabilization of bowl-shaped calixpyrroles, where typically an acetonitrile molecule redirects the CH_3_ group toward π-systems of four tilted pyrroles.^50,51^ It is noteworthy that X-ray quality monocrystals of this habit (the plates) could only be obtained in the presence of acetonitrile, showing the importance of interactions with this solvent for crystal growth. The difference in the gap sizes between **3-NN** and **3-TeTe** is not merely the crystal-packing function or the intercalation effect but an intrinsic property of the molecule, as shown by the Density Functional Theory (DFT) studies. In the geometries obtained by DFT optimization in a vacuum or in the presence of solvents treated as a Polarizable Continuum Model (PCM), the substantial difference in spacing was retained, although both structures remained slightly more open due to the absence of the crystal lattice (calculated centroids distances, vacuum: 5.53 Å for **3-TeTe** vs 6.83 Å for **3-NN**). In the acetonitrile complex, CH_3_CN⊃**3-NN**, reproduced by DFT calculations, the macrocycle geometry was more compact than that in a vacuum. The C–H···π interactions, between CH_3_CN and two central pyrrole rings, on the overall molecular geometry were reflected by the shortening of the pyrrole···pyrrole distance. Pyrrole centroids in **3-NN** optimized in vacuum were separated by 6.83 Å, while in CH_3_CN⊃**3-NN** by 6.72 Å, which was close to the value 6.70 Å obtained in the X-ray crystal structure. The calculated complexation energy reached an unexpectedly large, as for noncovalent interactions, value of −17.25 kcal/mol, however, comparable to other calculated energies of (pyrrole)_2_·acetonitrile complexes found in the literature, ranging from −11.6 to −15.2 kcal/mol, depending on the geometry.^52^

**Figure 3:**
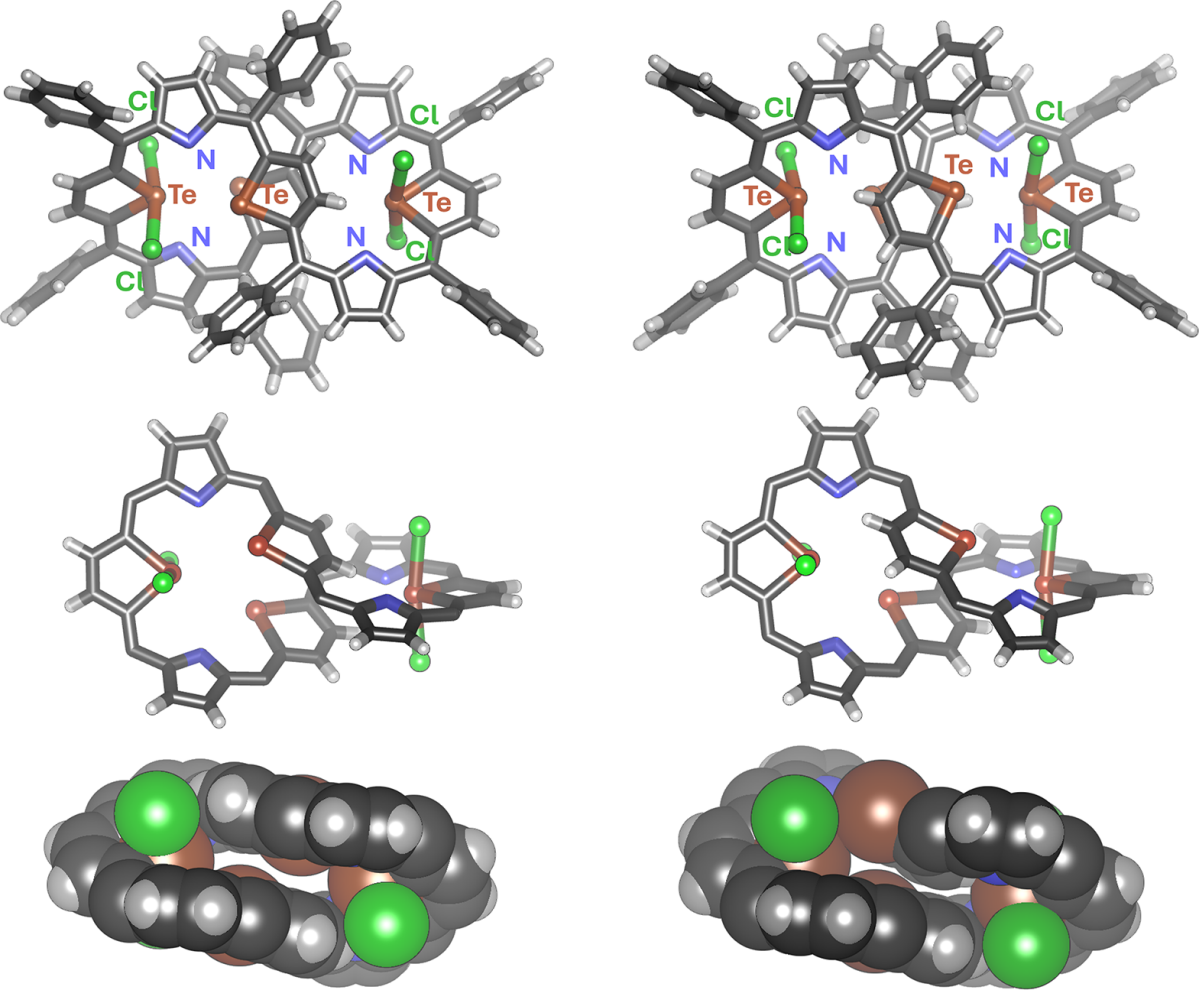
X-ray molecular structures of **3-Cl**_**4**_**-a** (left) and **3-Cl**_**4**_**-p** (right); *M,M* enantiomer shown; in the bottom projection (CPK) phenyl rings are omitted for clarity.

Crystallization of **3-Cl**_**4**_ produced two types of crystals as well, comprising macrocycles shaped according to the two variations of the **TeTe** layout, both with chlorinated tellurophenes at the figure-eight extremes and bare tellurophenes on the ribbon crossing. The conformer **3-Cl**_**4**_**-a** (for *a*ntiparallel) found in crystals grown from a *n*-hexane/chloroform solvent system (*C*2/*c*), showed close resemblance to the **3-TeTe** conformer, having the tellurophenes on the intersection directed antiparallelly (Figure 3 and Figure S27 in the Supporting Information). The other form detected in the second type of crystals, obtained from an acetonitrile/chloroform mixture (*P*), exhibited lower molecular symmetry, *C*_1_, with *p*arallel orientation of two central tellurophene rings (**3-Cl**_**4**_**-p**; Figure 3 and Figure S26 in the Supporting Information). The overall geometry, including small tellurophene···tellurophene distances (centroid···centroid: 4.07 Å for **3-Cl**_**4**_**-p** and 4.64 Å for **3-Cl**_**4**_**-a**) resembles that derived from the **3-TeTe** crystal structure. The DFT structures calculated for vacuum showed slightly more expanded 3D structures, with the respective distances of 5.06 and 5.45 Å. The positions of chlorine atoms (Cl49 and Cl50) in the X-ray structures of **3-Cl**_**4**_**-a** and **3-Cl**_**4**_**-p** were found to be not fully occupied, with the site occupancy factors of 0.85 and 0.80, respectively. This is due to the spontaneous reductive elimination occurring for **3-Cl**_**4**_ in solution during slow single crystal growth and is in accordance with the spectroscopically observed reactivity.

**Figure 4:**
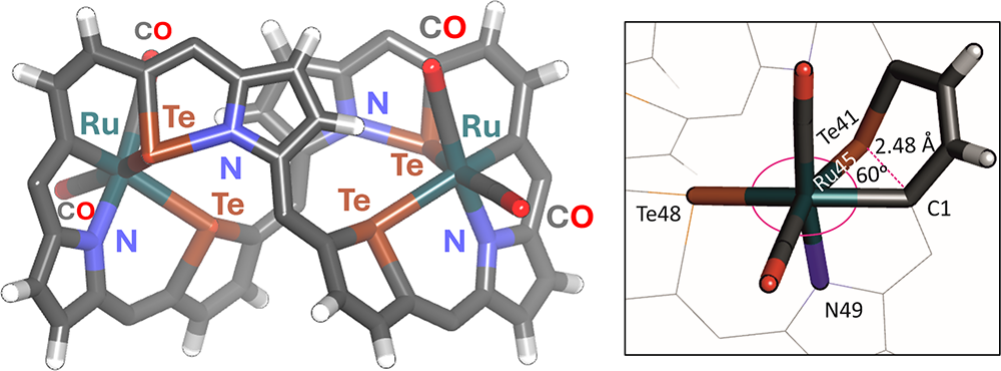
X-ray molecular structure of **4**; the aryl rings are omitted for the sake of clarity. Selected bond lengths around Ru in Å: Ru–Te41 2.6475(13), Ru–Te48 2.6595(12), Ru–N49 2.100(9), and Ru–C1 2.130(12).

The crystallization of **4**, obtained from an ethyl acetate/propan-1-ol mixture, did not give a diversity of crystal structures, as observed in the case of **3** and **3-Cl**_**4**_. The ruthenium binding markedly rigidified the tetratellura[36]octaphyrin structure, as evidenced by the ^1^H NMR studies described below. The crystal structure revealed near *C*_2_ molecular symmetry of **4**, which adopted the shape of a figure-eight with two pyrrole rings at the ribbon-crossing, similarly to **3-NN**, and two symmetrically arranged ruthenium(II) centers with identical octahedral environment (Te_2_NC_3_) within two ditelluraisophlorin-like helical pockets (Figure 4 and Figure S28 in the Supporting Information). The coordination sphere of each ruthenium(II) ion occupied by four donor atoms originating from the macrocyclic ligand (Te_2_NC) was completed with two carbonyl ligands. The chemical bond lengths around the metal center were in the range typical for ruthenium(II). The η^1^-coordination of the tellurophene-Te48 unit to ruthenium(II) exhibited a side-on geometry typically observed in metal complexes with telluraorganic ligands; however, the angle between the C–Te–C plane and the Te–Ru vector was relatively large (132°), as compared to the values reported in the literature for telluraorganic ligands coordinating to ruthenium (106–117°).^51^ The coordination mode of tellurophene rings in telluraporphyrinoids may be strongly influenced by the constraints enforced by the macrocycle, leading to atypical geometries. An acute angle between the C–Te–C plane and the Te–Pd vector, 88°, has been observed in a strongly folded carbazole-based ditelluraisophlorin palladium(II) complex, while in 21-metalla-23-telluraporphyrins, analogous values for Te–Pd, Te–Pt, and Te–Rh bonds are relatively large, 131–147°.^47,53,54^

The insertion of ruthenium(0) into the Te–C bond of the tellurophene ring of **3** resulted in the formation of a six-membered 1-ruthena-2-telluracyclohexadiene ring, RuTeC_4_, with a strongly folded geometry. This ring is characterized by an exceptionally small C1–Ru–Te41 angle (60°), which is indicative of a strained molecular structure. The geometry of the RuTeC_4_ unit was largely conserved from the original tellurophene ring of the substrate **3**, as reflected by a very short interatomic distance, 2.454(12) Å, between the trigonal C1 and Te41 in **4**. This distance exceeds the typical Te–C σ-bond length (2.0–2.2 Å);^51^ however, it still remained within “exceptionally long bonding distance”, previously found for the donor–acceptor C–Te interactions (2.34–2.53 Å).^55,56^ The proximity of the nitrogen N42, which is not coordinated by the central metal ion, and tellurium Te41, with a distance of 2.137(9) Å (or 2.123(10) Å for Te46–N47), suggests the presence of a covalent interaction. This is consistent with the range of 2.15–2.35 Å observed in telluraorganic compounds with trivalent tellurium.^51^ This interaction was found to account for the stability and rigidity of the helical structure. Hypervalent tellurium–nitrogen interactions have been observed within the cores of other telluraporphyrinoids, such as 21-carba-23-telluraporphyrin (Te–N 2.521(2) Å),^57^ where adjacent tellurophene and pyrrolenine units remain coplanar. The Te···N separations in the free tetratelluraoctaphyrin **3** were significantly larger than the above but still indicated the existence of interactions, which may be important for the overall conformation (the shortest in **3-NN** are Te43···N42 2.631(4) Å, Te47···N46 2.731(5) Å; the shortest in **3-TeTe** are Te43···N42 2.749(13) Å, Te47···N46 2.753(13) Å, while the sum of van der Waals radii is 3.65 Å^58^).

## NMR Studies and Dynamics

**Figure 5:**
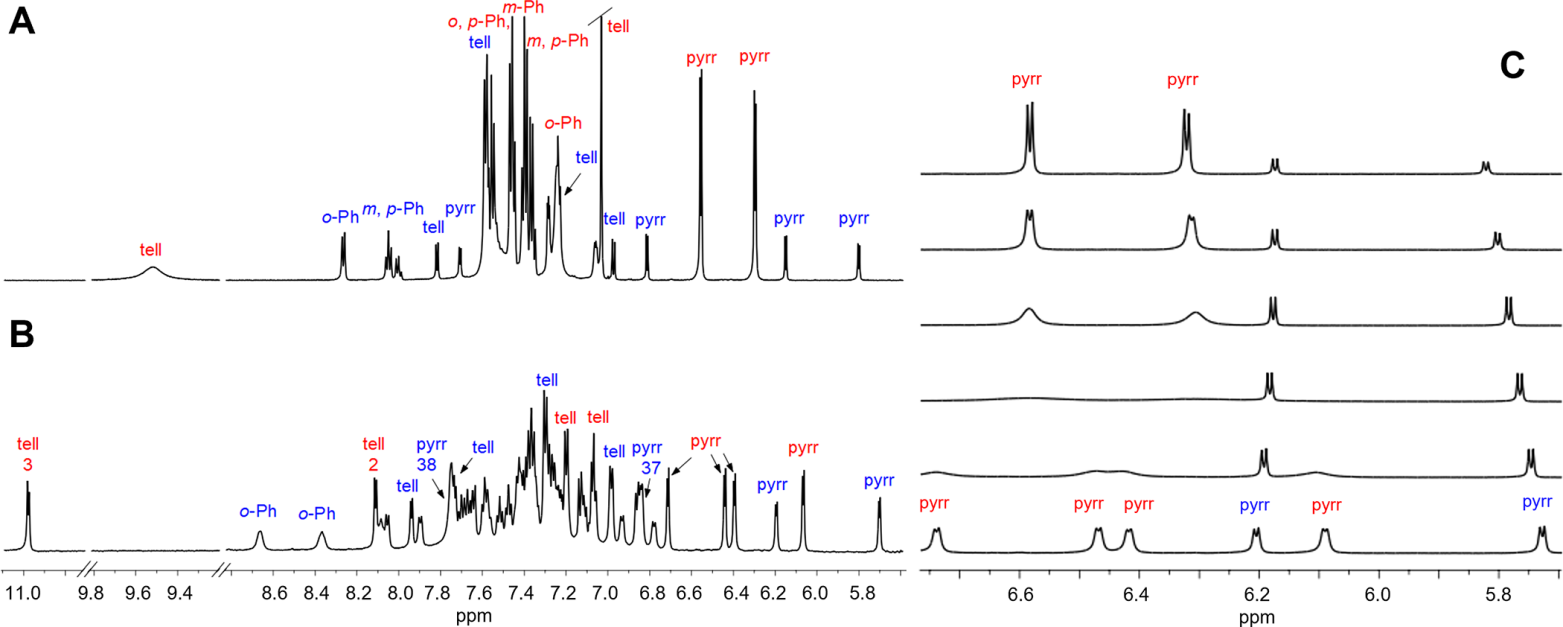
Selected region of ^1^H NMR spectra of **3** (CD_2_Cl_2_). (A) 300 K, (B) 182 K. (C) Variable temperature studies for the most informative region (from the top: 300, 279, 258, 235, 213, and 192 K). Signals of **3-TeTe** are marked in red, and those of **3-NN** are marked in blue.

**Scheme 3:**
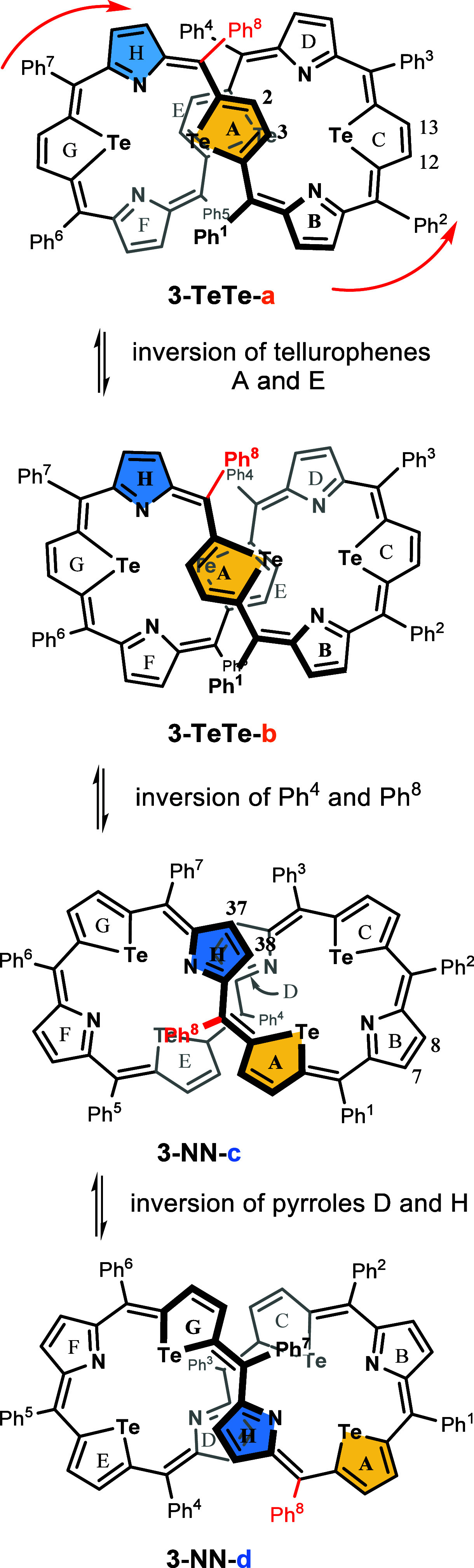
Dynamic Behavior of **3** Two selected heterocyclic rings and one phenyl ring are colored to emphasize the sequential motion of the molecule. Only the *M,M* enantiomer is shown. The red arrows indicate the conveyor-belt movement of the chain.

The ^1^H NMR spectrum recorded for the CD_2_Cl_2_ solution of **3** at 300 K (Figure 5) clearly displayed two sets of signals of different intensities, corresponding to the existence of various crystal forms of the macrocycle in the solid state. The less intense set includes eight doublets assigned to β-pyrrole and β-tellurophene protons based on ^3^*J*_HH_ values (4.6 and 5.6–7.1 Hz, respectively), which is, in terms of symmetry, in accordance with the expected figure-eight molecular shape. The more intense signal set, with two doublets assigned to pyrrole rings (^3^*J* = 4.6 Hz) and two tellurophene singlets, implied a higher molecular symmetry than any of the forms present in the solid state. The NOESY spectrum (300 K, CD_2_Cl_2_) provided evidence of an exchange of β-tellurophene protons between and within the forms; the same occurred for β-pyrrole peaks (Figure S5 in the Supporting Information). These observations, together with severe broadening of the tellurophene signal at 9.53 ppm, indicated the dynamic exchange between the two forms of **3** and the exchange of proton positions within each species. The variable temperature ^1^H NMR studies conducted within the range of 182–350 K in CD_2_Cl_2_ and C_6_D_6_ (Figure 5C and Figures S2–S4 in the Supporting Information) and supported by 2D spectra, provided insight into the dynamic behavior of molecule **3** in solution. The NMR studies allowed us to conclude that solution structures possess the main characteristics of **3-NN** and **3-TeTe**, found in the solid state. The dynamic behavior accounting for the spectral changes was described by the reaction sequence presented in Scheme 3.

Upon cooling, the dynamic processes slowed down, allowing to register at 182 K (CD_2_Cl_2_) a spectrum consistent with the presence of two different forms of *C*_2_ symmetry in solution. The assignment of two sets of signals to the structures **3-NN** and **3-TeTe** was based on the characteristic AX system of β-protons with a large chemical shift span, which was attributed to β-protons of the heterocyclic rings at the figure-eight crossing. In both structures of **3**, as well as in similar molecules reported in the literature, such as octatolyltetrathiaoctaphyrins^15^ or octaaryloctaphyrins,^22,59^ this central heterocycle is flanked by two phenyl rings with alternating orientations (Figure 2; Scheme 3), resulting in the shielding of only one β-proton by the aryl. The contribution of a residual paratropic ring current of the macrocycle to this differentiation may also be of importance. According to the popular annulene model of porphyrinoid aromaticity, the macrocycle **3** can be treated as a system with [4*n*] π-electrons (i.e., 36) on the conjugation pathway. The figure-eight geometry, equivalent to a 360°-twisted system, is regarded to obey the Hückel rule for [4*n*] π-conjugated rings,^9,16,60,61^ although advanced calculations of the current–density pathways show that the aromaticity in figure-eights is much more complicated.^62^ Thus, in **3-TeTe**, the proton H2 (see the structure of **3-TeTe-a** in Scheme 3) on the central tellurophene ring A is shielded by the nearby Ph^8^ ring, while the proton H3 is unaffected by the adjacent Ph^1^, which is oriented toward the tellurium atom. In addition, H3 is positioned directly above the center of the semicavity, formed by the BCDE rings, which is characterized by a Nucleus Independent Chemical Shift (NICS) value of +4.0, while H2 is pointing to the side. Therefore, the set of signals comprising an AX system of two remote doublets (δ 10.99 (H3) and 8.11 (H2) at 182 K) with the ^3^*J*_HH_ value typical for tellurophene rings (5.3 Hz) is attributed to the **3-TeTe** form, with tellurophene rings (A, E) at the crossing. The other set of signals, attributed to the **3-NN** form, displays the diagnostic AX system at δ 7.75 (H38) and 6.85 (H37; see **3-NN-c** in Scheme 3), with the coupling constant of ^3^*J*_HH_ = 4.5 Hz (235 K), indicating that the pyrrole rings are situated at the molecule crossing (rings D, H). The proximal semicavity, formed by ABCD rings, characterized by NICS = +6.1, is situated relatively far from H38, since the **3-NN** form has a large intersection pitch. This signal assignment is consistent with the assignment for the sulfur analogue.^15^

In a solution of **3** at room temperature, the two forms remain in the state of equilibrium, **3-TeTe** ⇌ **3-NN**, due to the exchange evidenced by relevant EXSY signals on a NOESY map. At 300 K the **3-TeTe** form prevails; however, the equilibrium constant defined as the molar ratio *K* = [**3-NN**]:[**3-TeTe**] depends strongly on the solvent. These ratios determined from integral intensities at 300 K are 1:9 in CDCl_3_, 1:2.8 in CD_2_Cl_2_, and 1:1.3 in C_6_D_6_. With increasing temperature the equilibrium **3-TeTe** ⇌ **3-NN** shifts to the left, with the *K* reaching the lowest observed ratio, 1:12, at 330 K in CDCl_3_. The solubility of the octaphyrin **3** in acetonitrile was so low that a good quality ^1^H NMR spectrum could not be obtained, but an addition of deuterated acetonitrile to a chloroform solution shifted the equilibrium to the left (from 1:9 in pure CDCl_3_ (300 K) to 1:1.5 for CD_3_CN/CDCl_3_, 1:0.7). Decreasing the temperature shifts the equilibrium toward **3-NN**, resulting in an increase in the *K*. However, below 260 K, the interconversion slows down significantly and does not allow the equilibrium to be reached. In CDCl_3_ at 270 K, relevant EXSY correlations were no longer detected on the NOESY map. Furthermore, the ratio of the two forms determined from the integral intensities of the spectra recorded at 182 K strongly depended on the sample cooling rate, demonstrating that no equilibrium was reached at this temperature. As the temperature dropped, the solubility of **3** decreased, causing the compound to precipitate and the concentration of **3-NN** selectively to decrease. The question of the thermodynamic stability of the conformers was raised, and the standard thermodynamic parameters, Δ*H*° and Δ*S*°, were calculated according to the van’t Hoff equation based on the data from the temperature dependence of the *K* value for the temperature ranges, where the equilibrium was achieved (Figure S7, Table S1 in the Supporting Information). The values of Δ*H*° and Δ*S*° of the reaction **3-TeTe** ⇌ **3-NN** range from −1.8(1) kcal/mol and 10.5(4) cal/mol·K, respectively, calculated for chloroform solution (260–330 K), through −2.6(2) kcal/mol and 10.6(5) cal/mol·K for dichloromethane (260–300 K), to −4.7(3) kcal/mol and 16.0(9) cal/mol·K for benzene (300–350 K). The DFT calculations performed for **3-NN** and **3-TeTe** molecules in vacuum and in the presence of the above solvents treated as a Polarizable Continuum Model (PCM) did not correctly reproduce either the relative energy or the strong dependence on the solvent (Table S2 in the Supporting Information). Interactions between solvents and tetratellura[36]octaphyrin are likely more specific than those predicted by the PCM model due to the presence of cavities of different sizes in two forms of **3**. Such specific interactions of **3-NN** with the acetonitrile molecule, as detected in the solid state and reproduced by the DFT calculations, may be important in solution. Moreover, the significantly narrower cavity in the **3-TeTe** molecule is unlikely to allow similar intercalation of a solvent molecule. We presume that specific noncovalent interactions strongly influence the equilibrium constant values in different solvents.

**Figure 6:**
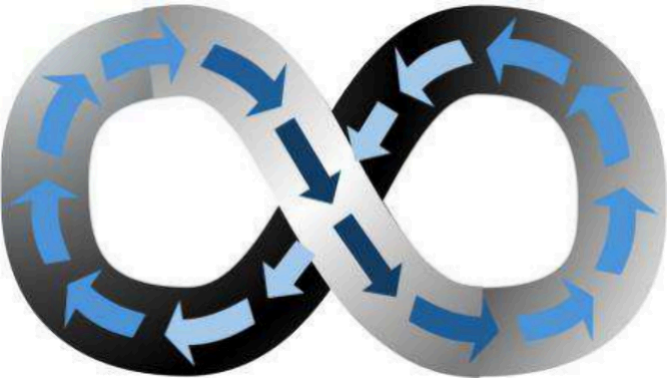
Conveyor-belt movement of an octaphyrin.

The dynamic behavior of the tetratellura[36]octaphyrin presented in Scheme 3 has been separated into three subsequent steps that can be detected spectroscopically. The isomerization reaction discussed above, depicted as the reaction second step, **3-TeTe-b** ⇌ **3-NN-c**, is slow on the ^1^H NMR time scale in the whole applied temperature range (182–350 K; Figures S2–S4 in the Supporting Information). This molecular motion consists of Ph^8^ and Ph^4^ phenyl rings flip and a displacement of heterocyclic rings along the figure-eight ribbon, also observed for the thiophene analogue of **3** and described as a conveyor-belt movement (Figure 6).^15^ The dynamic process, which accounts for the averaging of tellurophene and pyrrole signals of the **3-TeTe** form at room temperature, corresponds to the relatively fast isodynamic interconversion between two identical forms, **3-TeTe-a** ⇌ **3-TeTe-b**. The movement involves inversion of two central tellurophene rings (A and E) and again a displacement along the macrocyclic chain. The coalescence temperatures, equal to 270 K for tellurophene A and E signals and 240 K for pyrrole signals in CDCl_3_, stand for the free activation enthalpy, Δ*G*^≠^, of approximately 11 kcal/mol. The second isodynamic equilibrium, **3-NN-c** ⇌ **3-NN-d**, is slow on the ^1^H NMR time scale over the entire 182–350 K range and involves a turnover of the two middle pyrroles D and H, apart from the conveyor-belt movement. Scheme 3, which presents the dynamic behavior of enantiomer *M,M*, does not include a possible interconversion of the enantiomers, which has not been studied by means of NMR. Attempts to separate the enantiomers of **3** at room temperature using HPLC with a chiral stationary phase showed that racemization is fast, with a rate constant *k* = 5(1) min^–1^, corresponding to an enantiomer half-life of less than 0.2 min. The presence of an intense EXSY signal on a NOESY map (C_6_D_6_ solution of **3** at 350 K, Figure S6 in the Supporting Information) connecting C (G) and A (E) tellurophene protons may indicate their direct exchange which would operate in the case of the stretching–compressing mechanism of the enantiomers’ interconversion.^24^ For **3-TeTe**, such a mechanism would involve pulling the A and E tellurophene rings in opposite directions along a line defined by the centroids of A and E, so that these two rings reach the extremes of an inverted figure-eight. An alternative movement responsible for this EXSY correlation would require a multistep “conveyor-belt-like” motion.

**Scheme 4:**
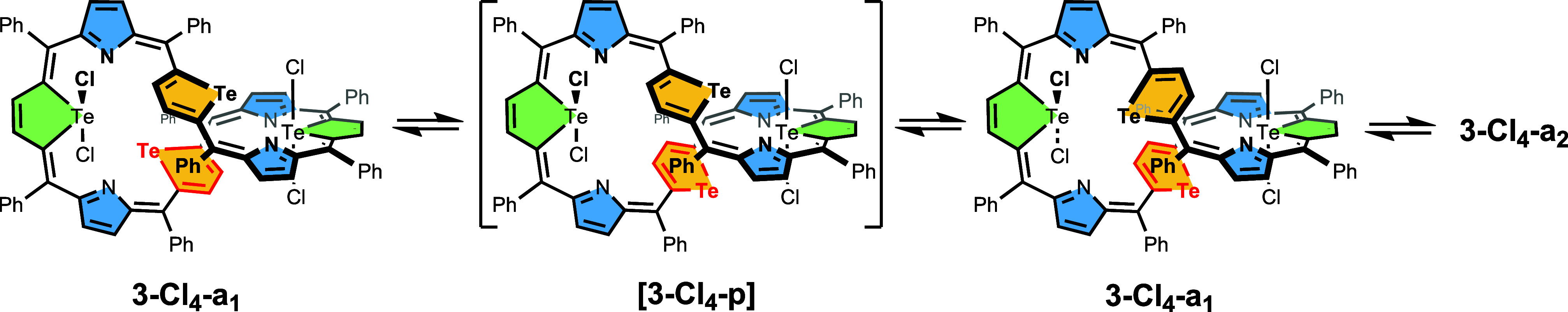
Dynamic Processes in Solution of **3-Cl**_**4**_

The ^1^H NMR spectra of **3-Cl**_**4**_ (Figures S12–S14 in the Supporting Information) also indicated the presence of two interconverting forms in solution, both of which displayed spectral patterns similar to that of **3-TeTe**. The variable temperature measurements gave evidence of the dynamic behavior of **3-Cl**_**4**_ in solution and the interconvertibility of the two forms. As the temperature decreased, the most downfield broad signal, observed at 9.24 ppm at 300 K (CD_2_Cl_2_), split into two very broad signals, found at 10.35 and around 8.0 ppm at 235 K, which subsequently, at 192 K, separated into two pairs of doublets of different intensity (1:2), belonging to two conformers of **3-Cl**_**4**_ (Figure S13 in the Supporting Information). These two AX spin systems were assigned to β-tellurophene protons at the molecule crossing (C and G rings in **3-Cl**_**4**_**-a**, Scheme 4) based on the observation that the large chemical shift spans observed for each of the AX systems were diagnostic for the **TeTe** structure. The solid-state studies of **3-Cl**_**4**_ also evidenced the existence of two forms, namely with antiparallel and parallel tellurophene rings at the figure-of-eight center, differing in the molecular symmetry: *C*_2_ for **3-Cl**_**4**_**-a** and *C*_1_ for **3-Cl**_**4**_**-p**. Accordingly, for **3-Cl**_**4**_**-a** we anticipated the presence of two β-tellurophene signals in the downfield region, whereas for **3-Cl**_**4**_**-p** we expected to observe four such signals. However, both forms observed by ^1^H NMR had the same number of signals consistent with *C*_2_ molecular symmetry. The observed inconsistency could not be explained by a fast on the ^1^H NMR time scale motion of the molecule **3-Cl**_**4**_**-p**, since such a motion would have to average the resonance positions of the protons at the figure-eight crossing, characterized by a large chemical resonance frequency difference (Δδ = 2.3 ppm). Thus, the two *C*_2_ forms observed in solution most likely represent two species analogous to **3-Cl**_**4**_**-a** observed in the solid state, differing in a subtle way in their conformations, which may be denoted as **3-Cl**_**4**_**-a**_**1**_ and **3-Cl**_**4**_**-a**_**2**_. The form **3-Cl**_**4**_**-p**, documented only in the solid state, can provide a model of an intermediate between the two symmetric **3-Cl**_**4**_**-a** forms interconverting via central tellurophene ring rotation. According to DFT calculations, the symmetric **3-Cl**_**4**_**-a** form is 3.7 kcal/mol more stable than that of **3-Cl**_**4**_**-p**.

The ^1^H NMR characteristics of **4**, with narrow β-proton lines, and their independence from temperature are consistent with a relatively rigid macrocyclic skeleton, with only the phenyl rings undergoing rotation (Figures S18–S19 in the Supporting Information). The distinctive feature of the spectrum is the presence of an AX spin system with a large coupling constant (^3^*J*_HH_ = 9.3 Hz), which is diagnostic of the six-membered RuTeC_4_ unit. A similar ^3^*J* value (10.8 Hz) has been considered a spectroscopic evidence for the presence of a RhTeC_4_ unit in the course of 21-rhoda-23-telluraporphyrin formation.^47^ For metalatelluracycles containing osmium, ruthenium, and iron, the coupling constants of similar values (9.7–11.1 Hz)^44^ have been reported. The chemical shifts of the β-protons of **4** were located within a narrow range between 6.24 and 7.09 ppm; therefore, we considered the compound to be a nonaromatic macrocycle. The X-ray structure analysis allowed us to indicate the spots interrupting the π-orbitals overlap along the expected conjugation path, that is, large dihedral angles equal to ∠60° (between planes defined by C19–C20–C21 and C20–C21–C22) and ∠56° (planes C1–C2–C3 and C40–C1–C2).

## Conclusions

We have demonstrated that tetratellura[36]octaphyrin, **3**, the largest expanded telluraporphyrin to date, can be readily obtained from a one-pot synthesis from pyrrole and a tellurophene-containing diol. This macrocycle provides a valuable opportunity for studies on the coordination chemistry, anion-binding properties, and postsynthetic modifications of the expanded heteroporphyrin. The well-known reactivity of tellurophene as a porphyrinoid building block, prone to transformations to several other units, for example metallacyclopentadiene, makes **3** a reactive starting point for a variety of novel porphyrinoids, including metallaporphyrinoids and vacataporphyrinoids. The observed reactivity of **3** was generally tellurium-centered, while the macrocyclic perimeter proved to be robust.

The tetratelluraoctaphyrin **3** exists in two figure-eight-shaped conformations in the solid state and in solution, exhibiting notable flexibility in the latter. The conformation with two tellurophene units at the figure-of-eight crossing was stabilized by the oxidative addition of chlorine to two tellurium atoms. On the other hand, the geometry with two pyrrole rings in the center of the molecule was fixed during the metalation of **3** with ruthenium, resulting in the formation of **4** with a reduced macrocyclic skeleton, which can be regarded as an expanded telluraisophlorin. The rigid chiral structure of **4** contrasts with the substantial flexibility of tetratelluraoctaphyrin **3** in solution.

The reactivity of tetratellura[36]octaphyrin toward triruthenium dodecacarbonyl showed ruthenium(0) to be capable of tellurium–carbon bond activation. Two of the four tellurophene rings of **3** transformed into strongly folded 1-ruthena-2-telluracyclohexadiene units. The organometallic product **4** revealed the carbaporphyrinoid nature of tetratellura[36]octaphyrin and represents a rare mode of metal binding by porphyrinoids. Metalation of **3** with triruthenium dodecacarbonyl represents the first example of an expanded porphyrinoid reactivity leading to a metallaporphyrinoid and encourages further studies in this field.

## Experimental Section

### Synthesis of 5,10,15,20,25,30,35,40-Octaphenyl-41,43,45,47-tetratellura-[36]octaphyrin(1.1.1.1.1.1.1.1) **3**

Pyrrole (90 μL, 1.3 mmol), 2,5-bis(phenylhydroxymethyltellurophene) (0.50 g, 1.3 mmol), and CH_2_Cl_2_ (500 mL) were placed in a 1 L flask with a reflux condenser. Nitrogen was bubbled through the solution for 15 min and then methanesulfonic acid (50 μL, 0.7 mmol) was added, and the mixture was stirred for 1 h under nitrogen in the dark. Chloranil (0.93 g, 3.9 mmol) was added, and the solution was refluxed for 30 min. The reaction mixture was filtered through basic alumina to remove tar products (basic alumina, Brockmann activity grade III). The products were eluted with CH_2_Cl_2_, evaporated, and subjected to column chromatography (CH_2_Cl_2_, basic alumina, Brockmann activity grade I). The first fraction comprised two green compounds, **2** and **3**. The mixture was separated using either (1) size-exclusion column chromatography (toluene) or (2) thin-layer chromatography on silicagel (CH_2_Cl_2_/*n*-hexane, 1:1, v/v), yielding the forest green 5,10,15,20-tetraphenyl-21,23-ditelluraporphyrin, **2**, and the desired sea green 5,10,15,20,25,30,35,40-octaphenyl-21,23,25,27-tetratelluraoctaphyrin(1.1.1.1.1.1.1.1), **3**. Yield 7% (37 mg) for (1) purification method; 5% (27 mg) for (2) method.

Note: Additional spectroscopic data and charts with abbreviations are provided in the Supporting Information.

****3**^1^H NMR (600 MHz, CD_2_Cl_2,_ 300 K)** δ the more abundant conformer, **3-TeTe** (75%): 9.53 (br.s, 4H, tell), 7.58 (br.d, ^3^*J*_HH_ = 7.3 Hz, 8H, *o*-Ph_i_), 7.55 (tt, ^3^*J*_HH_ = 7.6 Hz, ^4^*J*_HH_ = 1.3 Hz, 4H, *p*-Ph_i_), 7.45 (t, ^3^*J*_HH_ = 7.6 Hz, 8H, *m*-Ph_i_), 7.38 (m, 2H, *m*-Ph_o_), 7.36 (br.m, 2H, *p*-Ph_o_), 7.24 (m, 4H, *o*-Ph_o_), 7.03 (s, 4H, tell), 6.55 (d, ^3^*J*_HH_ = 4.6 Hz, 4H, pyrr), 6.28 (d, ^3^*J*_HH_ = 4.6 Hz, 4H, pyrr); the less abundant conformer, **3-NN** (not all signals are visible at 300 K) (25%): 8.27 (d, ^3^*J*_HH_ = 7.3 Hz, 4H, *o*-Ph_i_), 8.05 (t, ^3^*J*_HH_ = 7.6 Hz, 4H, *m*-Ph_i_), 8.0 (d, ^3^*J*_HH_ = 7.5 Hz, 2H, *p*-Ph_i_), 7.82 (d, ^3^*J*_HH_ = 5.6 Hz, 2H, tell), 7.71 (d, ^3^*J*_HH_ = 4.6 Hz, 2H, pyrr), 7.53 (d, ^3^*J*_HH_ = 5.6 Hz, 2H, tell), 7.60–7.27 (m, 26H, Ph), 7.23 (d, ^3^*J*_HH_ = 6.9 Hz, 2H, tell), 7.05 (m, 4H, Ph), 6.97 (d, ^3^*J*_HH_ = 6.9 Hz, 2H, tell), 6.80 (d, ^3^*J*_HH_ = 4.6 Hz, 2H, pyrr), 6.14 (d, ^3^*J*_HH_ = 4.6 Hz, 2H, pyrr), 5.79 (d, ^3^*J*_HH_ = 4.6 Hz, 2H, pyrr). ^**1**^**H NMR (600 MHz, CD_2_Cl_2,_ 182 K)** δ 10.99 (d, ^3^*J*_HH_ = 5.3 Hz, 2H, tell_TeTe_), 8.67 (br.s, 2H, *o*-Ph), 8.37 (br.s, 2H, *o*-Ph), 8.11 (d, ^3^*J*_HH_ = 5.3 Hz, 2H, tell_TeTe_), 8.08 (m, 4H, 2 × Ph), 7.93 (d, ^3^*J*_HH_ = 5.4 Hz, 2H, tell_NN_), 7.90 (br.d, 2H, Ph), 7.74 (br.s, 2H, pyrr_NN_), 7.73 (d, ^3^*J*_HH_ = 5.4 Hz, 2H, tell_NN_), 7.70–7.26 (m, Ph_NN_,Ph_TeTe_), 7.23 (br.d, ^3^*J*_HH_ = 6.9 Hz, 2H, tell_NN_), 7.19 (d, ^3^*J*_HH_ = 6.9 Hz, 2H, tell_TeTe_), 7.11 (br.d, 2H, Ph), 7.06 (m, 6H, 2 × Ph, tell_TeTe_) 6.98 (br.d, ^3^*J*_HH_ = 6.9 Hz, 2H, tell_NN_), 6.92 (br.d, 2H, Ph), 6.85 (m, 6H, Ph, pyrr_NN,_ tell_TeTe_), 6.77 (br.d, 2H, Ph), 6.70 (d, ^3^*J*_HH_ = 4.5 Hz, 2H, pyrr_TeTe_), 6.43 (d, ^3^*J*_HH_ = 4.5 Hz, 2H, pyrr_TeTe_), 6.38 (d, ^3^*J*_HH_ = 4.2 Hz, 2H, pyrr_TeTe_), 6.18 (br.d, 2H, pyrr_NN_), 6.05 (d, ^3^*J*_HH_ = 4.2 Hz, 2H, pyrr_TeTe_), 5.69 (br.d, 2H, pyrr_NN_). ^13^**C NMR (150 MHz, CD_2_Cl_2_, 182 K)** δ 174.0 (α-pyrr_NN_), 171.7 (α-pyrr_TeTe_), 169.9 (α-pyrr_TeTe_), 162.4 (α-tell_TeTe_/*meso*_TeTe_), 159.3 (α-pyrr_NN_), 157.6 (Ph), 155.5 (α-pyrr_TeTe_), 152.3 (α-pyrr_TeTe_), 150.8 (Ph), 150.3 (α-tell_TeTe_/*meso*_TeTe_), 149.1 148.5 (α-tell_NN_), 147.2 (β-tell_TeTe_), 143.4 (α-tell_TeTe_), 143.0 (β-tell_NN_), 143.1 (β-pyrr_NN_/β-tell_NN_), 142.9 (Ph), 142.2 (β-tell_NN_), 141.8 (β-tell_NN_), 141.3 (Ph), 138.2 137.9, 137.7, 137.2, 137.0 (β-pyrr_TeTe_), 136.8 (β-tell_TeTe_), 136.3, 135.9 (β-pyrr_TeTe_), 134.3 (β-pyrr_NN_), 132.5, 133.9 (β-pyrr_NN_/β-tell_NN_), 131.5, 131.1 (β-tell_TeTe_), 130.7 (β-tell_TeTe_), 129.9, 129.8 (β-pyrr_TeTe_), 129.2, 129.0 (β-pyrr_TeTe_), 128.5, 128.3, 128.1, 128.0 (β-pyrr_NN_), 127.6, 127.5, 127.3 (β-pyrr_NN_), 127.1, 126.7. **UV–vis (CH_2_Cl_2_) λ_max_[nm] (logε)** = 415 (4.5), 675 (4.5). **HRMS (ESI)***m*/*z* = 1680.0713, calc. for [C_88_H_56_N_4_^128^Te_4_]^+^, [M]^+^: 1680.0795; *m*/*z* = 840.0349 calc. for [C_88_H_56_N_4_^128^Te_4_]^2+^, [M]^2+^: 840.0354. **Crystal data** for compound **3-NN**: C_88_H_56_N_4_Te_4_·3.99(C_2_H_3_N)·0.3(CHCl_3_), *M* = 1879.79, monoclinic, *P*2_1_/*c*, *a* = 24.172(4) Å, *b* = 18.142(3) Å, *c* = 19.690(3) Å, β = 97.36(2)°, *V* = 8563(2) Å^3^, *Z* = 4, *D*_c_ = 1.458 g·cm^–3^, *T* = 100(2) K, *R* = 0.078, *wR* = 0.244 10693 reflections with *I* > 2σ(*I*)) for 1084 parameters, CCDC 2376409. **Crystal data** for compound **3-TeTe**: C_88_H_56_N_4_Te_4_·0.9(CHCl_3_), *M* = 1794.06, monoclinic, *C*2/*c*, *a* = 29.676(2) Å, *b* = 13.112(6) Å, *c* = 37.779(3) Å, β = 98.62(1)°, *V* = 14534(7) Å^3^, *Z* = 8, *D*_c_ = 1.640 g·cm^–3^, *T* = 100(2) K, *R* = 0.087, *wR* = 0.257 (5969 reflections with *I* > 2σ(*I*)) for 898 parameters, CCDC 2376410.

### Synthesis of **3-Cl**_**4**_

The reaction was carried out in an NMR tube under an ^1^H NMR control. Gaseous chlorine was added by a gastight syringe in 25 μL portions to the solution of **3** (5.0 mg, 4.7 × 10^–3^ mmol) in chloroform-*d* (0.6 mL) until **3** reacted completely to yield **3-Cl**_**4**_. Another portion of chlorine gas was added when no more changes on the ^1^H NMR spectrum were observed for 1–2 h; in practice, the sample was left to react overnight after each batch, so the reaction was carried out for several days, typically four. The yield was practically quantitative; the sample was used without further purification.

Note: If the chlorine portions were added at 15 min intervals, a mixture of products was formed. The product **3-Cl**_**4**_ is sensitive to water and methanol and decomposes during column chromatography; when stored in chloroform-*d* for several hours at room temperature, it undergoes spontaneous reductive elimination to **3**. Despite numerous attempts, we were unable to obtain a ^13^C NMR spectrum of **3-Cl**_**4**_ due to severe broadening of signals at room temperature and the compound’s very low solubility at low temperatures.

**^1^H NMR (600 MHz, CD_2_Cl_2_, 300 K)** δ 9.24 (br.s, 2H, tell), 7.42 (m, 40 H, Ph), 7.03 (s, 2H, tell), 6.80 (d, ^3^*J*_HH_ = 4.6 Hz, 2H, pyrr), 6.44 (d, ^3^*J*_HH_ = 4.6 Hz, 2H, pyrr); ^**1**^H NMR (600 MHz, CD_2_Cl_2,_ 182 K); δ the more abundant conformer (68%) 10.35 (d, ^3^*J*_HH_ = 5.1 Hz, 2H, tell), 7.89 (d, ^3^*J*_HH_ = 5.1 Hz, 2H, tell), 7.73–7.03 (m, 44H, tell, Ph), 6.90 (d, ^3^*J*_HH_ = 4.5 Hz, 2H, pyrr), 6.90 (d, ^3^*J*_HH_ = 4.5 Hz, 2H, pyrr), 6.64 (d, ^3^*J*_HH_ = 4.5 Hz, 2H, pyrr), 6.62 (d, ^3^*J*_HH_ = 4.5 Hz, 2H, pyrr); the less abundant conformer (not all signals are visible) (32%) 10.34 (d, ^3^*J*_HH_ = 5.0 Hz, 2H, tell), 8.33 (d, ^3^*J*_HH_ = 5.0 Hz, 2H, tell), 7.73–7.03 (m, 44H, tell, Ph), 6.84 (br.m, 4H, 2 × pyrr), 6.56 (d, ^3^*J*_HH_ = 4.3 Hz, 2H, pyrr), 6.37 (br.d, 2H, pyrr). **UV–vis (CH**_**2**_**Cl**_**2**_**) λ**_**max**_**[nm] (logε)** = 405 (4.4), 622 (4.4). **HRMS (ESI)***m*/*z* = 1777.0654 calc. for [C_90_H_62_N_4_O_2_^128^Te_4_^35^Cl]^+^, [M – 3Cl + 2OCH_3_]^+^: 1777.0761; *m*/*z* = 1711.0806 calc. for [C_89_H_59_N_4_O^128^Te_4_]^+^, [M – 4Cl + OCH_3_]^+^: 1711.0897, *m*/*z* = 871.0512, calc. for [C_90_H_62_N_4_O_2_Te_4_]^+^, [M – 4Cl + 2OCH_3_]^2+^: 871.0538. **Crystal data** for compound **3-Cl**_**4**_**-a**: C_88_H_56_N_4_Cl_3.40_Te_4_·C_6_H_14_·1.2(CHCl_3_)·0.4(CH_2_Cl_2_), *M* = 2063.68, monoclinic, *C*2/*c*, *a* = 35.746(1) Å, *b* = 14.049(4) Å, *c* = 18.438(5) Å, β = 91.19(2)°, *V* = 9257(4) Å^3^, *Z* = 4, *D*_c_ = 1.481 g·cm^–3^, *T* = 100(2) K, *R* = 0.058, *wR* = 0.130 (5694 reflections with *I* > 2σ(*I*)) for 644 parameters, CCDC 2376411. **Crystal data** for compound **3-Cl**_**4**_**-p**: C_88_H_56_N_4_Cl_3.60_Te_4_·0.9(CHCl_3_)·0.4(CH_2_Cl_2_)·1.5(C_2_H_3_N), *M* = 2001.67, triclinic, *P*, *a* = 10.587(8) Å, *b* = 19.263(16) Å, *c* = 20.915(17) Å, α = 99.61(2)°, β = 90.19(2)°, γ = 97.74(2)°, *V* = 4166(6) Å^3^, *Z* = 2, *D*_c_ = 1.596 g·cm^–3^, *T* = 100(2) K, *R* = 0.032, *wR* = 0.085 (13924 reflections with *I* > 2σ(*I*)) for 1213 parameters, CCDC 2376412.

### Reduction of **3-Cl**_**4**_ to **3**

The reaction was performed in a glass vial; 5 mg (2.8 × 10^–6^ mmol) of **3-Cl**_**4**_ was dissolved in CH_2_Cl_2_ (2 mL), and 50 mg (2.8 × 10^–4^ mmol) sodium dithionite solution in water (2 mL) was added. The mixture was stirred for 15 min. The organic layer containing the resulting product, **3**, was filtered through basic Al_2_O_3_. The solvent was evaporated. Yield 65% (3 mg).

### Synthesis of **4**

Tetratellura[36]octaphyrin **3** (10.0 mg, 6.0 × 10^–3^ mmol) and [Ru_3_(CO)_12_] (15.2 mg, 2.4 × 10^–2^ mmol) were dissolved in 20 mL of toluene. The solution was refluxed for 10 min. The solvent was removed using a rotary evaporator, and the product was purified by thin-layer chromatography (SiO_2_, CH_2_Cl_2_/*n*-hexane, 4:1, v/v). The first fraction contained a mixture of unknown side products with a yield of less than 1%. The second brown fraction was comprised of pure compound **4**. Yield 34% (4 mg). The use of the more hazardous benzene as the reaction solvent led to a substantial increase in yield of up to 70% (8 mg).

**^1^H NMR (600 MHz, CD_2_Cl_2_, 300 K)** δ 7.71 (d, ^3^*J*_HH_ = 7.4 Hz, 2H, *o*-Ph), 7.58 (td, ^3^*J*_HH_ = 7.4 Hz, ^4^*J*_HH_ = 1.8 Hz, 2H, *m*-Ph), 7.48 (tt, ^3^*J*_HH_ = 7.3 Hz, ^4^*J*_HH_ = 1.4 Hz, 2H, *p*-Ph) 7.47 (d, ^3^*J*_HH_ = 7.0 Hz, 4H, *o*-Ph), 7.43 (tt, ^3^*J*_HH_ = 7.4 Hz, ^4^*J*_HH_ = 1.2 Hz, 2H, *p*-Ph), 7.40 (s, 10H, Ph), 7.27 (br.m, 6H, *o*-Ph, *m*-Ph), 7.09 (d, ^3^*J*_HH_ = 9.3 Hz, 2H, RuTeC_4_), 7.07 (br.s, 4H, *o*-Ph), 6.94 (t, ^3^*J*_HH_ = 7.4 Hz, 2H, *m*-Ph), 6.87 (d, ^3^*J*_HH_ = 9.3 Hz, 2H, RuTeC_4_), 6.78 (d, ^3^*J*_HH_ = 3.6 Hz, 2H, pyrr), 6.74 (d, ^3^*J*_HH_ = 4.2 Hz, 2H, pyrr), 6.63 (d, ^3^*J*_HH_ = 7.0 Hz, 2H, tell), 6.40 (d, ^3^*J*_HH_ = 4.2 Hz, 2H, pyrr) 6.27 (d, ^3^*J*_HH_ = 3.6 Hz, 2H, pyrr), 6.24 (d, ^3^*J*_HH_ = 7.0 Hz, 2H, tell); **^**13**^C NMR (125 MHz, CD_2_Cl_2_, 300 K)** δ 201.0 (CO), 197.4 (CO), 167.1 (Ph), 155.7 (α-pyrr), 148.3 (α-pyrr), 147.5 (α-pyrr), 142.5 141.8 141.7 141.4 (β-RuTeC_4_), 141.3, 139.9 (β-tell), 138.8 138.5 137.5 133.2 (β-tell), 132.2 (Ph), 130.8 (*o*-Ph), 129.8 (Ph), 129.6 (α-RuTeC_4_), 129.2 (Ph), 129.0 (*m*-Ph), 128.8 128.7 128.6 (Ph), 128.5 (Ph), 128.3 (β-RuTeC_4_), 128.1 123.9 (β-pyrr), 123.1 (α-tell), 120.5 (β-pyrr), 119.9 (β-pyrr), 119.5 (α-tell), 119.2 (α-RuTeC_4_), 112.3 (β-pyrr). **UV–vis (CH**_**2**_**Cl**_**2**_**) [nm] λ**_**max**_**(logε)** = 295 (4.8), 381 (4.3), 476 nm (4.2), 544 (4.1), 647 (3.9). **HRMS (ESI)**, *m*/*z* = 1994.8578, calc. for [C_88_H_56_N_4_^128^Te_4_^101^Ru_2_]^+^_,_ [M]^+^: 1994.8620; *m*/*z* = 997.4290 calc. for [C_88_H_56_N_4_^128^Te_4_^101^Ru_2_]^2+^, [M]^2+^: 997.4307. **Crystal data** for compound **4:** C_92_H_56_N_4_O_4_Ru_2_Te4·(C_4_H_8_O_2_)·0.9(C_3_H_8_O), *M* = 2136.13, monoclinic, *P*2_1_/*c*, *a* = 20.286(1) Å, *b* = 24.515(1) Å, *c* = 19.946(1) Å, β = 113.60(1)°, *V* = 9089(10) Å^3^, *Z* = 4, *D*_c_ = 1.561 g·cm^–3^, *T* = 100(2) K, *R* = 0.077, *wR* = 0.237 (10188 reflections with *I* > 2σ(*I*)) for 1228 parameters, CCDC 2376413; **IR (KBr) v**_**CO**_**=** 1969 cm^–1^, 2001 cm^–1^, 2022 cm^–1^, 2059 cm^–1^.

## Supporting Information Available

Detailed spectral characterization, methods, and DFT-calculated structures coordinates (PDF)
