# Supplementary material for: Tetratellura[36]octaphyrin(1.1.1.1.1.1.1.1) Metalation: From Dynamic Behavior to Rigid Chiral Figure-of-Eight Molecule; Activation of the C–Te Bond by Ruthenium
Source: Inorg Chem. 2024 Nov 6;63(46):21788–800. doi: 10.1021/acs.inorgchem.4c03506 (PMC11577318; doi:10.1021/acs.inorgchem.4c03506)
Supplement: Supplementary file 1 — ic4c03506_si_001.pdf [file ic4c03506_si_001.pdf]

Supporting Information

Tetratellura[36]octaphyrin(1.1.1.1.1.1.1.1) metalation: from dynamic behavior to rigid chiral figure-of-eight molecule; activation of C-Te bond by ruthenium

*Paulina Krzyszowska, Emilia Ganczar, Piotr J. Chmielewski and Ewa Pacholska-Dudziak**

Department of Chemistry, University of Wroclaw, ul. Joliot-Curie 14, 50-383 Wroclaw, Poland

Corresponding Author (*)

Ewa Pacholska-Dudziak: ewa.dudziak@uwr.edu.pl

Table of Contents

Methods....4

Nuclear Magnetic Resonance....4

Mass spectrometry....4

UV-Vis spectroscopy....4

Electrochemistry....4

Circular dichroism spectroscopy....4

Infrared spectroscopy....4

Density Functional Theory Calculations....4

Figures and Tables....5

Figure S1. ^{1}H NMR spectrum of **3** (600 MHz, CD_2Cl_2, 300 K); * - H_2O and impurities. 5

Figure S2. Variable temperature ^{1}H NMR spectra (600 MHz, CD_2Cl_2, 192-300 K) of **3** (selected range). 6

Figure S3. Signals assignment; ^{1}H NMR spectra of **3**; 600 MHz, CD_2Cl_2, 300 K, 235 K, and 182 K (selected ranges). 7

Figure S4. ^{1}H NMR spectra (600 MHz, C_6D_6, 300-350 K) of **3** (selected range). 8

Figure S5. ^{1}H-^{1}H NOESY spectrum (600 MHz, CD_2Cl_2, 300 K) of **3** (selected range). 9

Figure S6. ^{1}H-^{1}H NOESY spectrum (600 MHz, C_6D_6, 350 K) of **3** (selected range). 10

Figure S7. Van't Hoff plots for the reaction **3-TeTe** ⇌ **3-NN** in benzene-d_6 (A), dichloromethane-d_2 (B), and chloroform-d (C) , $\ln K = -\Delta H°/R + \Delta S°/RT$, where K = [**3-NN**]/[**3-TeTe**] was obtained from VT ^{1}H NMR spectra. ..11

Table S1. Thermodynamic parameters for the reaction **3-TeTe** ⇌ **3-NN**, calculated from Van't Hoff plots (Figure S7). 11

Table S2. Calculated (DFT) energy differences, $\Delta E = E_{3\text{-NN}} - E_{3\text{-TeTe}}$ for different solvents (Polarizable Continuum Model (PCM)). Zero point energy (ZPE) correction applied. 11

Figure S8. ^{13}C NMR spectrum (150 MHz, CD_2Cl_2, 300 K) of **3**; (top: the whole spectral range, bottom: the most informative region; * = signals of n-hexane). The low intensity of several signals in the ^{13}C NMR spectrum is a result of broadening caused by chemical exchange....12

Figure S9. ^{1}H-^{13}C HSQC (red correlations) and ^{1}H-^{13}C HMBC (black correlations) spectra of **3**; 600 MHz, CD_2Cl_2, 300 K (selected range). 13

Figure S10. Selected regions of HRMS ESI (+MS) spectrum of **3**: a) measured (top) and simulated (bottom) calc. for $C_{88}H_{56}N_4Te_4$, $[M]^+$, b) measured (top) and simulated (bottom) calc. for $C_{88}H_{56}N_4Te_4$, $[M]^{2+}$. 14

Figure S11. Cyclic (CV) and differential pulse (DP) voltammograms recorded for **3** in CH_2Cl_2 with $[Bu_4N]PF_6$ as supporting electrolyte. The horizontal arrows indicate directions of the potential sweep, the numbers are DP peak potentials in volts. The electrochemical HOMO-LUMO gap is 1.23 V. 15

Figure S12. ^{1}H NMR spectrum of **3-Cl₄** (600 MHz, CD_2Cl_2, 300 K); * = H_2O, ethyl acetate, n-hexane. Signals of traces of **3** are visible....16

Figure S13. Variable temperature ^{1}H NMR spectra (selected range) of **3-Cl₄**; 600 MHz, CD_2Cl_2, 300-192 K. 17

Figure S14. ^{1}H NMR spectrum (600 MHz, CD_2Cl_2, 182 K) of **3-Cl₄** (selected range). Two forms are denoted by different colors. 18

Figure S15. ^{1}H-^{1}H NOESY spectrum (600 MHz, CD_2Cl_2, 192 K) of **3-Cl₂** (selected range)....18

Figure S16. Selected regions of HRMS ESI (+MS) spectrum **3-Cl$_4$** obtained in CH_3OH: a): measured (top) and simulated (bottom) calc. for $C_{88}H_{56}N_4Te_4Cl(OCH_3)_2$, $[M\text{-}3Cl\text{+}2OCH_3]^+$, b): measured (top) and simulated (bottom) calc. for $C_{88}H_{56}N_4Te_4(OCH_3)_2$, $[M\text{-}4Cl\text{+}OCH_3]^+$, c) measured (top) and simulated (bottom) calc. for $C_{88}H_{56}N_4Te_4(OCH_3)_2$, $[M\text{-}4Cl\text{+}2OCH_3]^{2+}$. 19

Figure S17. ^1H NMR spectrum of **4** (600 MHz, CD_2Cl_2, 300 K); * = H_2O and impurities. 20

Figure S18. ^1H NMR spectrum (600 MHz, CD_2Cl_2, 300 K) of **4** (selected range). 20

Figure S19. Variable temperature ^1H NMR spectra (600 MHz, CD_2Cl_2, 192-300 K) of **4** (selected range). 21

Figure S20. ^{13}C NMR (150 MHz, CD_2Cl_2, 300 K) spectrum of **4**; (top: the whole spectral range, bottom: the most informative region; * = n-hexane and impurities). 22

Figure *S*21. Selected regions of HRMS ESI (+MS) spectrum of **4**: a) measured (top) and simulated (bottom) calc. for $C_{88}H_{56}N_4Te_4Ru_2$, $[M]^+$, b) measured (top) and simulated (bottom) calc. for $C_{88}H_{56}N_4Te_4Ru_2$, $[M]^{2+}$. 23

Figure S22. Diffuse reflectance infrared Fourier transform (DRIFT) spectrum of **4** (**4** was diluted in KBr) 24

Figure S23. TDDFT-predicted UV-Vis and ECD spectra **4** - *PP* enantiomer and TDDFT-predicted UV-Vis spectra of two conformers of **3**. 25

X-ray crystallography 26

Table S3. Crystal data for **3-NN**, **3-TeTe**, **3-Cl$_4$-a**, **3-Cl$_4$-p**, and **4**. 27

Figure S24. X-ray molecular structure of **3-NN**·CH_3CN. Displacement ellipsoids represent 50% probability. In the side view (bottom), the aryl rings are shown as wireframes for clarity. 28

Figure S25. X-ray molecular structure of **3-TeTe**. Displacement ellipsoids represent 50% probability. In the side view (bottom), the aryl rings are shown as wireframes for clarity. 29

Figure S26. X-ray molecular structure of **3-Cl$_4$-p**. Displacement ellipsoids represent 50% probability. In the side view (bottom), the aryl rings are shown as wireframes for clarity. 30

Figure S27. X-ray molecular structure of **3-Cl$_4$-a**. Displacement ellipsoids represent 50% probability. In the side view (bottom), the aryl rings are shown as wireframes for clarity. 31

Figure S28. X-ray molecular structure of **4**. Displacement ellipsoids represent 50% probability. In the side view (bottom), the aryl rings are shown as wireframes for clarity 32

Table S4. Cartesian coordinates of DFT optimized structures of **3-NN**, **3-TeTe**, $CH_3CN \supset$**3-NN**, **3-Cl$_4$-a, 3-Cl$_4$-p** (B3PW91/SDD for Ru and Te, and 6-31G(d,p) for the rest of atoms) 33

References 40

Methods

Nuclear Magnetic Resonance

NMR spectra were recorded on Bruker Avance III 500 MHz, and 600 MHz spectrometers, and JEOL JNM-ECZ500R 500 MHz spectrometer. Chemical shifts were reported in ppm with reference to residual protons and carbons of $CDCl_3$ (δ 7.24 ppm in ^1H NMR), CD_2Cl_2 (δ 5.32 ppm in ^1H NMR, δ 54.0 ppm in ^{13}C NMR), and benzene-d_6 (δ 7.16 ppm in ^1H NMR).

Mass spectrometry

Mass spectra were carried out on a Bruker qTOF compact spectrometer using electrospray ionization.

UV-Vis spectroscopy

UV-Vis spectra were recorded on a Varian Carry 60 and Jasco V-770 spectrophotometers using 1 cm path length optical glass/quartz cuvettes.

Electrochemistry

Electrochemical measurements were performed by means of Autolab PGSTAT100N (Metrohm) potentiostat/galvanostat system for dichloromethane solutions with a glassy carbon, a platinum wire, and AgCl/Ag as the working, auxiliary, and reference electrodes, respectively. Tetrabutylammonium hexafluorophosphate ($[Bu_4N]PF_6$) was used as a supporting electrolyte. The given potentials were referenced with the ferrocene/ferrocenium couple used as an internal standard.

Circular dichroism spectroscopy

Circular dichroic spectrum was recorded by means of Jasco 1500 spectropolarimeter equipped with a flow cell attached to the Hitachi-Merck LaChrom HPLC system allowing detection of the chiral fraction and CD spectra measurement in a stopped-flow technique. Enantiomer resolutions were performed using either Chirex 3010 or Chirex 3014 column (25 × 0.46 cm).

Infrared spectroscopy

Diffuse reflectance infrared Fourier transform (DRIFT) spectrum was collected on Nicolet iS50 FT-IR Spectrometer (Thermo Scientific) with a Praying Mantis DRIFT accessory. The spectrum was collected in 4000-400 cm^{-1} range with number of scans set to 64. The sample was prepared under air atmosphere by grinding in a mortar with KBr and then placed under N_2 purge for collection time.

Density Functional Theory Calculations

Density Functional Theory (DFT) calculations were performed using the Gaussian 16 program.[S2] The initial structures for the optimization of **3-NN,** $CH_3CN{\supset}$**3-NN**, **3-TeTe**, **3-Cl$_4$-a**, **3-Cl$_4$-p** and **4** were derived from the crystal data. The geometry optimizations were carried out in the unconstrained C_1 symmetry in vacuo with the B3PW91 functional and mixed basis set (SDD with associated effective core potential for Te and Ru atoms, 6-31G(d,p) for remaining atoms). The modeling of solvatation effect by polarizable continuum model (PCM)[S3] did not give satisfying results. The existence of a local energy minimum was verified by a normal mode frequency calculation with no negative frequencies observed. The optimized geometries were used for TDDFT (**4**) and NICS (**3-NN** and **3-TeTe**) calculations. The 60 lowest-energy excited states were considered for TDDFT calculations. All relative energies include the zero-point correction. For the acetonitrile complex $CH_3CN{\supset}$**3-NN**, couterpoise correction was applied. The nucleus-independent chemical shifts (NICS) values were calculated using the GIAO method (Gaussian 16, B3PW91) at centroids between two tellurium atoms of one semiporphyrin moiety for **3-TeTe** and **3-NN**.

Figures and Tables

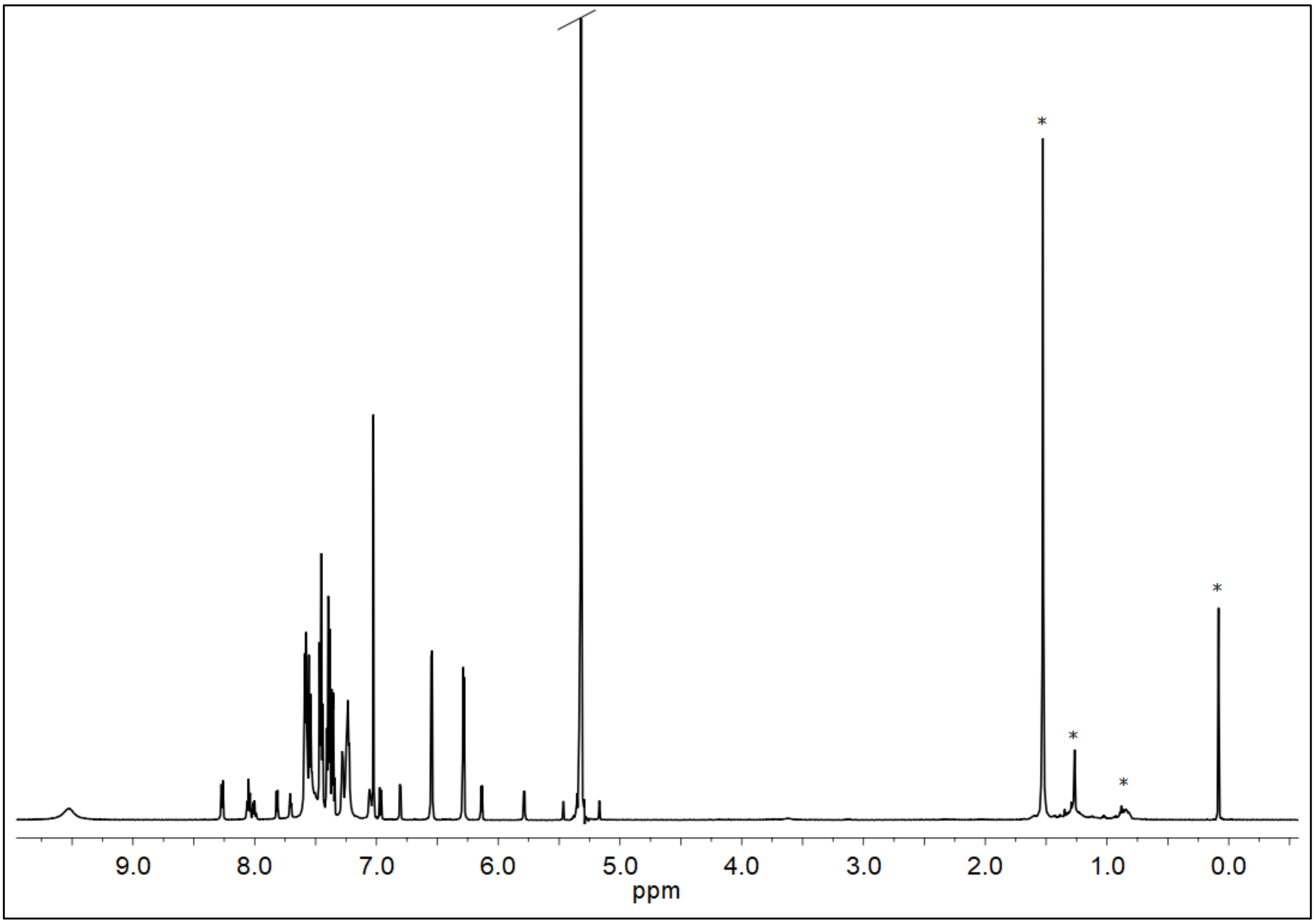

Figure S1. ^{1}H NMR spectrum of 3 (600 MHz, CD_2Cl_2, 300 K); * - H_2O and impurities.

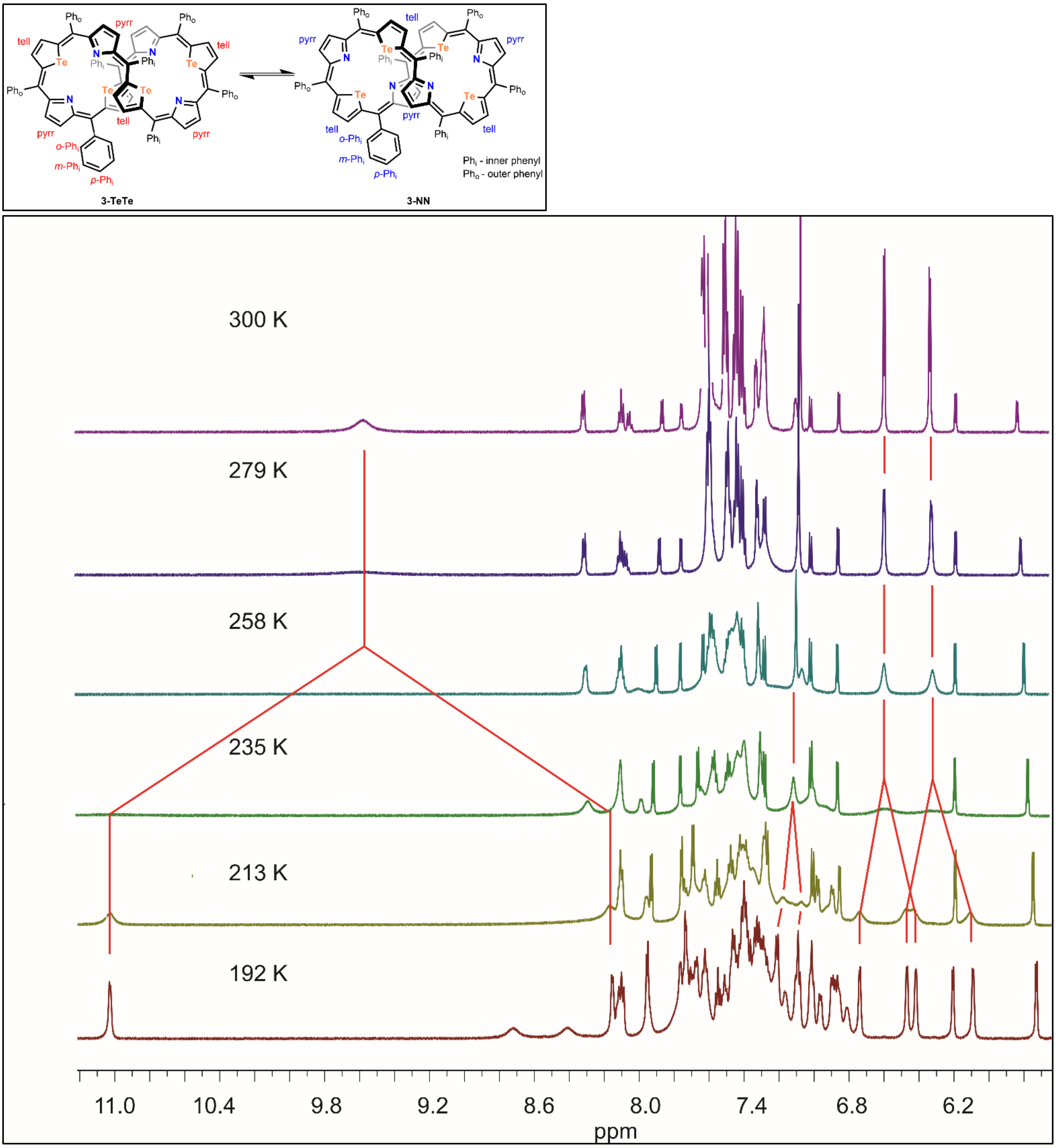

Figure S2. Variable temperature ^{1}H NMR spectra (600 MHz, CD_2Cl_2, 192-300 K) of 3 (selected range).

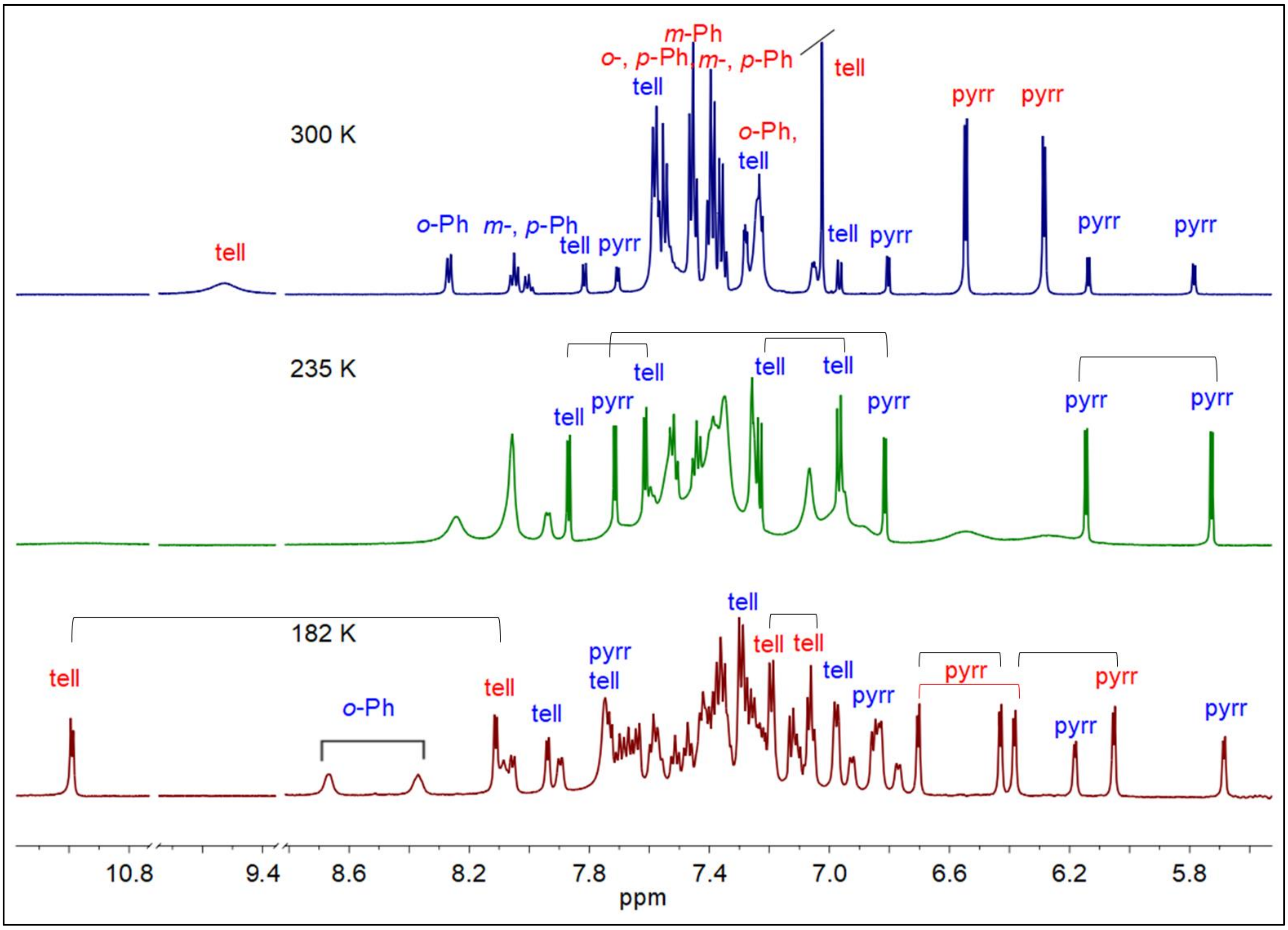

Figure S3. Signals assignment; ^{1}H NMR spectra of 3; 600 MHz, CD_2Cl_2, 300 K, 235 K, and 182 K (selected ranges). The blue captions denote conformer 3-NN, and the red captions denote conformer 3-TeTe.

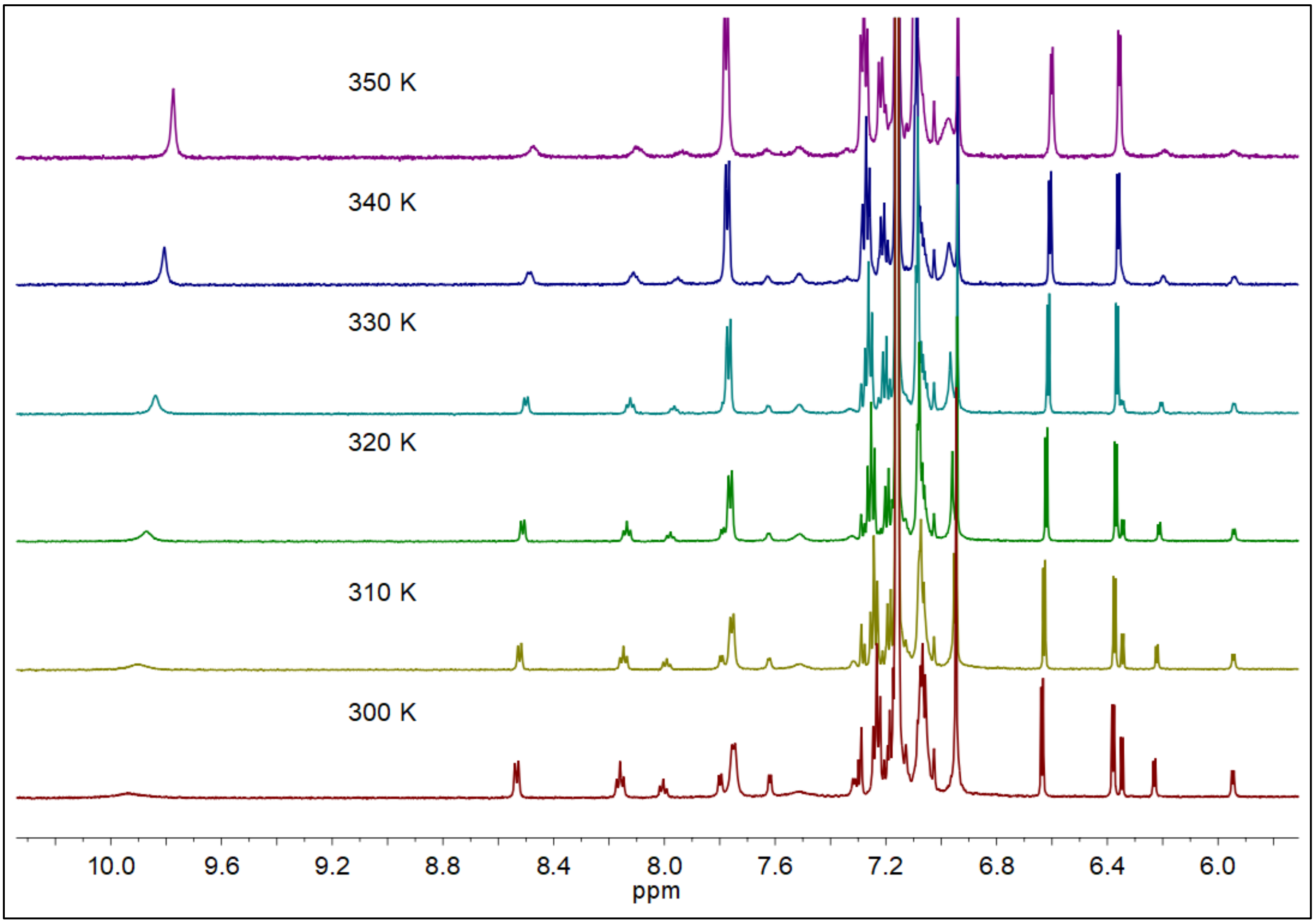

Figure S4. **^{1}H NMR spectra (600 MHz, C_6D_6, 300-350 K) of 3 (selected range).**

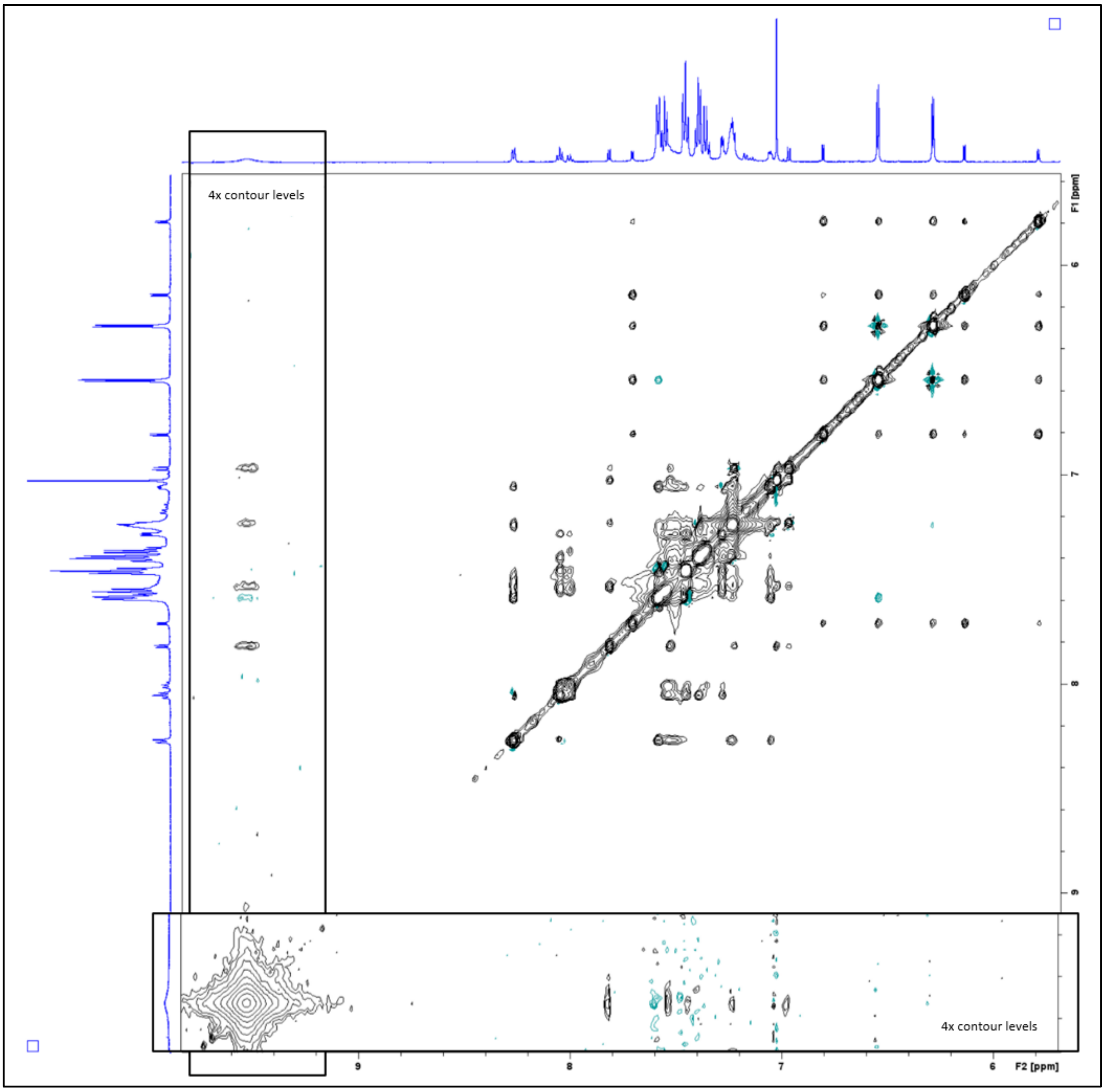

Figure S5. ^1H-^1H **NOESY spectrum (600 MHz, CD_2Cl_2, 300 K) of 3 (selected range).**

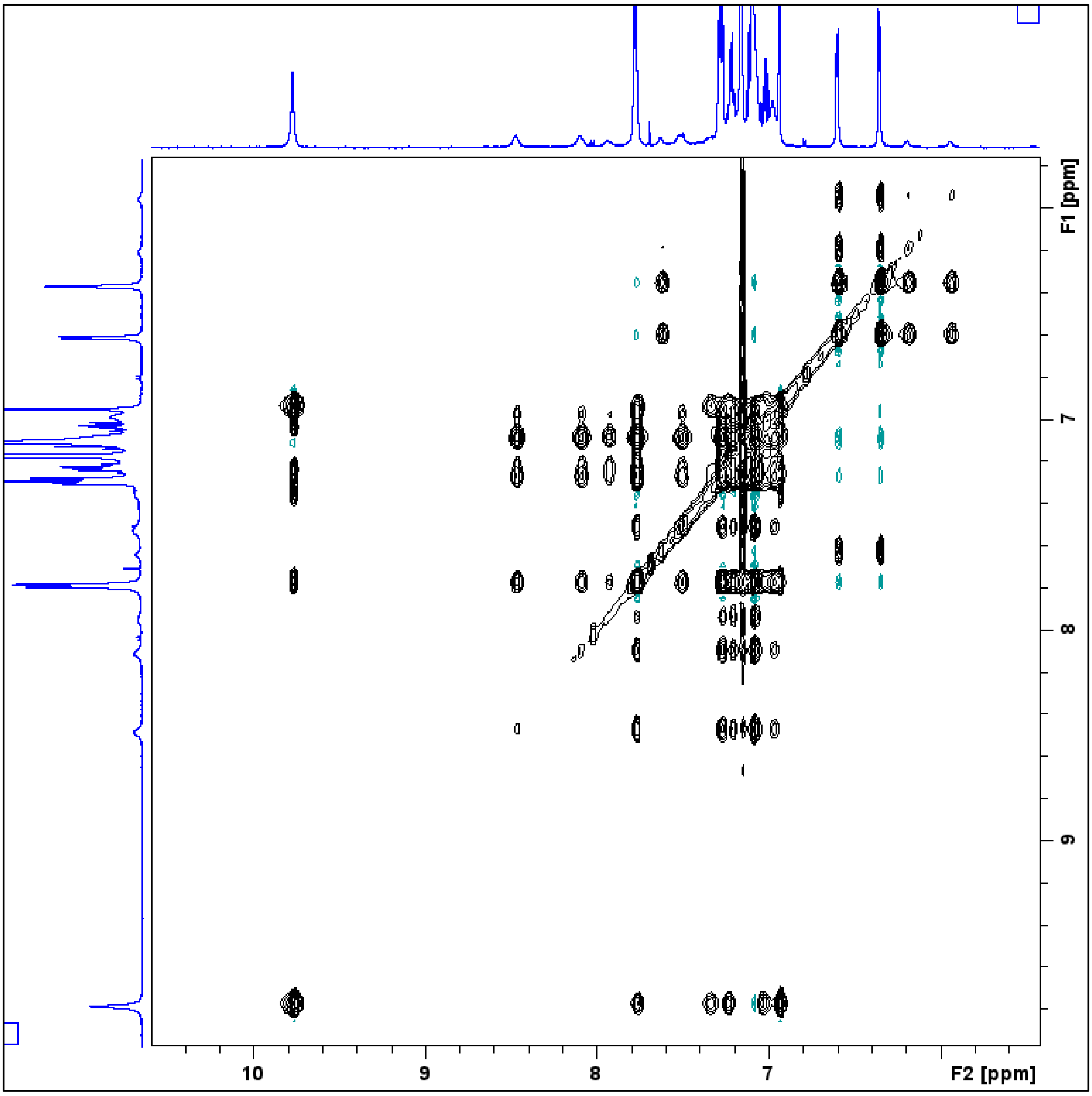

Figure S6. ^1H-^1H NOESY spectrum (600 MHz, C_6D_6, 350 K) of 3 (selected range).

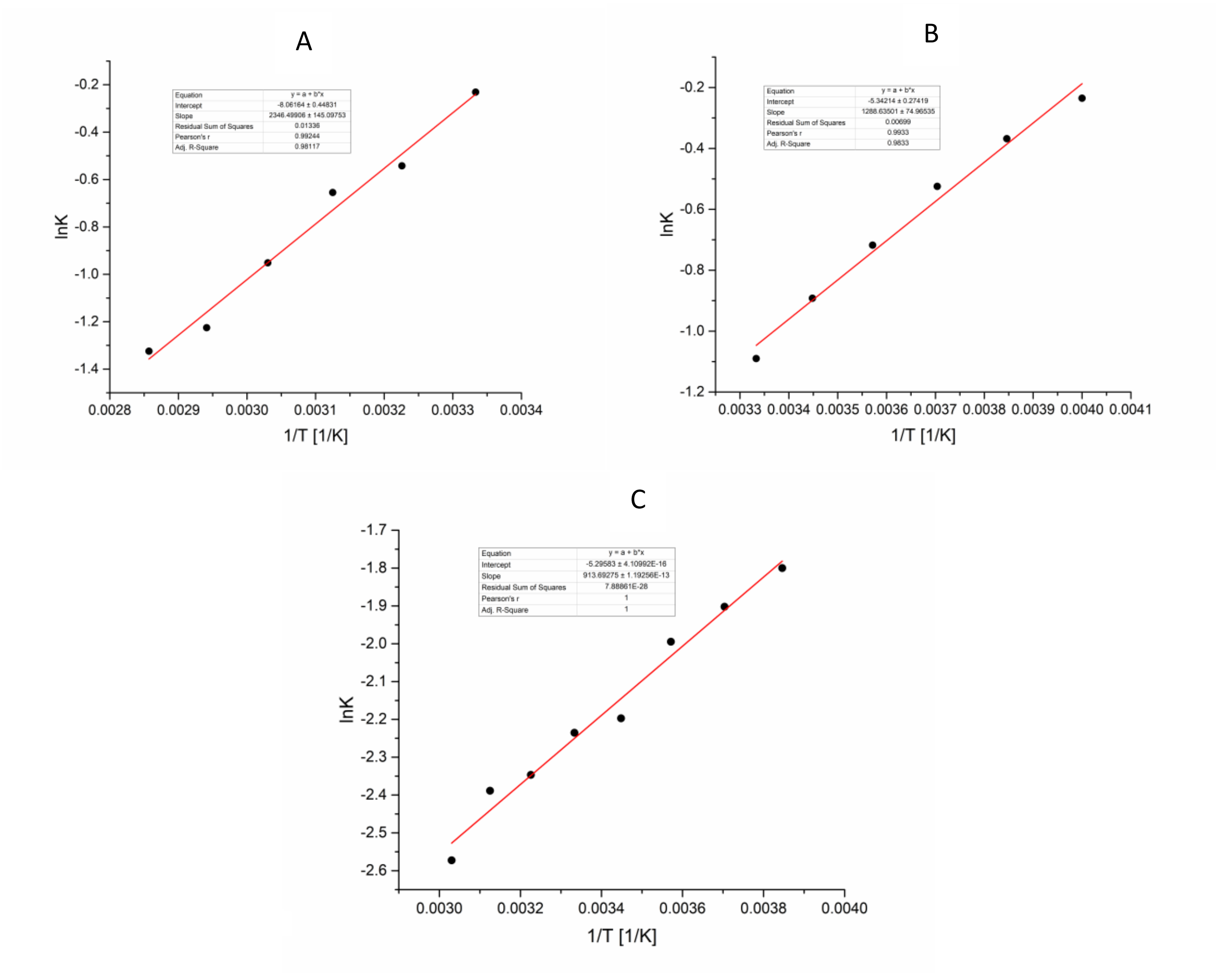

Figure S7. Van't Hoff plots for the reaction 3-TeTe ⇌ 3-NN in benzene-d$_6$ (A), dichloromethane-d$_2$ (B), and chloroform-d (C) , ln$K$ = $-\Delta H°/R + \Delta S°/RT$, where K = [3-NN]/[3-TeTe] was obtained from VT ^{1}H NMR spectra.

Table S1. Thermodynamic parameters for the reaction 3-TeTe ⇌ 3-NN, calculated from Van't Hoff plots (Figure S7).

	$CDCl_3$	CD_2Cl_2	C_6D_6
$\Delta H°$ (kcal/mol)	–1.8(1)	–2.6(2)	–4.7(3)
$\Delta S°$ (cal/mol·K)	10.5(4)	10.6(5)	16.0(9)

Table S2. Calculated (DFT) energy differences, $\Delta E = E_{3\text{-NN}} - E_{3\text{-TeTe}}$ for different solvents (Polarizable Continuum Model (PCM)). Zero point energy (ZPE) correction applied.

	DFT_{vacuum}	$DFT_{chloroform}$	$DFT_{dichlormeth.}$	$DFT_{benzene}$	$DFT_{acetonitrile}$
E_{ZPE} (E_{NN}-E_{TeTe}) kcal/mol	0.95	0.32	0.07	0.61	-0.31

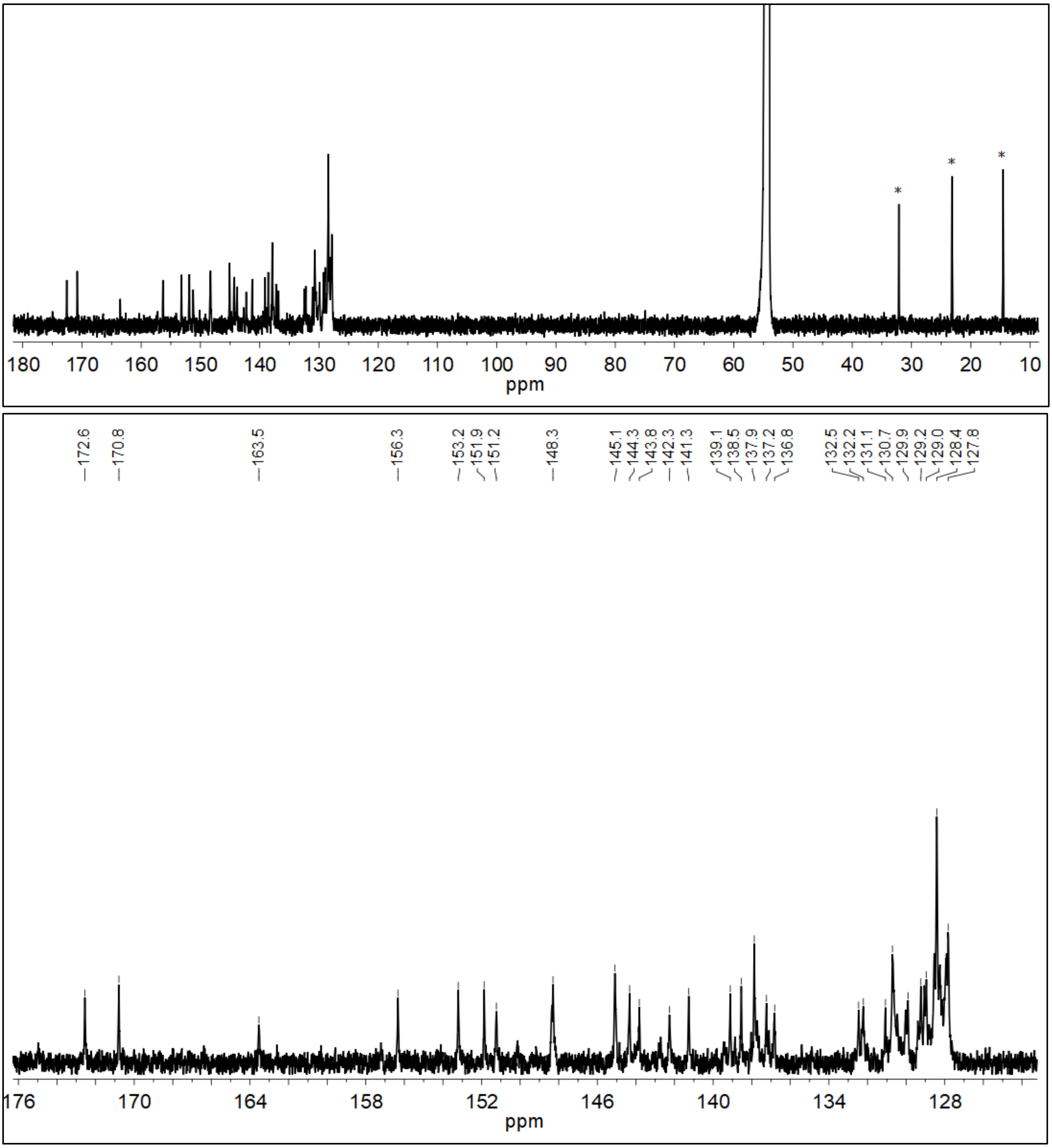

Figure S8. ^{13}C **NMR spectrum (150 MHz,** CD_2Cl_2**, 300 K) of 3; (top: the whole spectral range, bottom: the most informative region; * = signals of n-hexane). The low intensity of several signals in the** ^{13}C **NMR spectrum is a result of broadening caused by chemical exchange.**

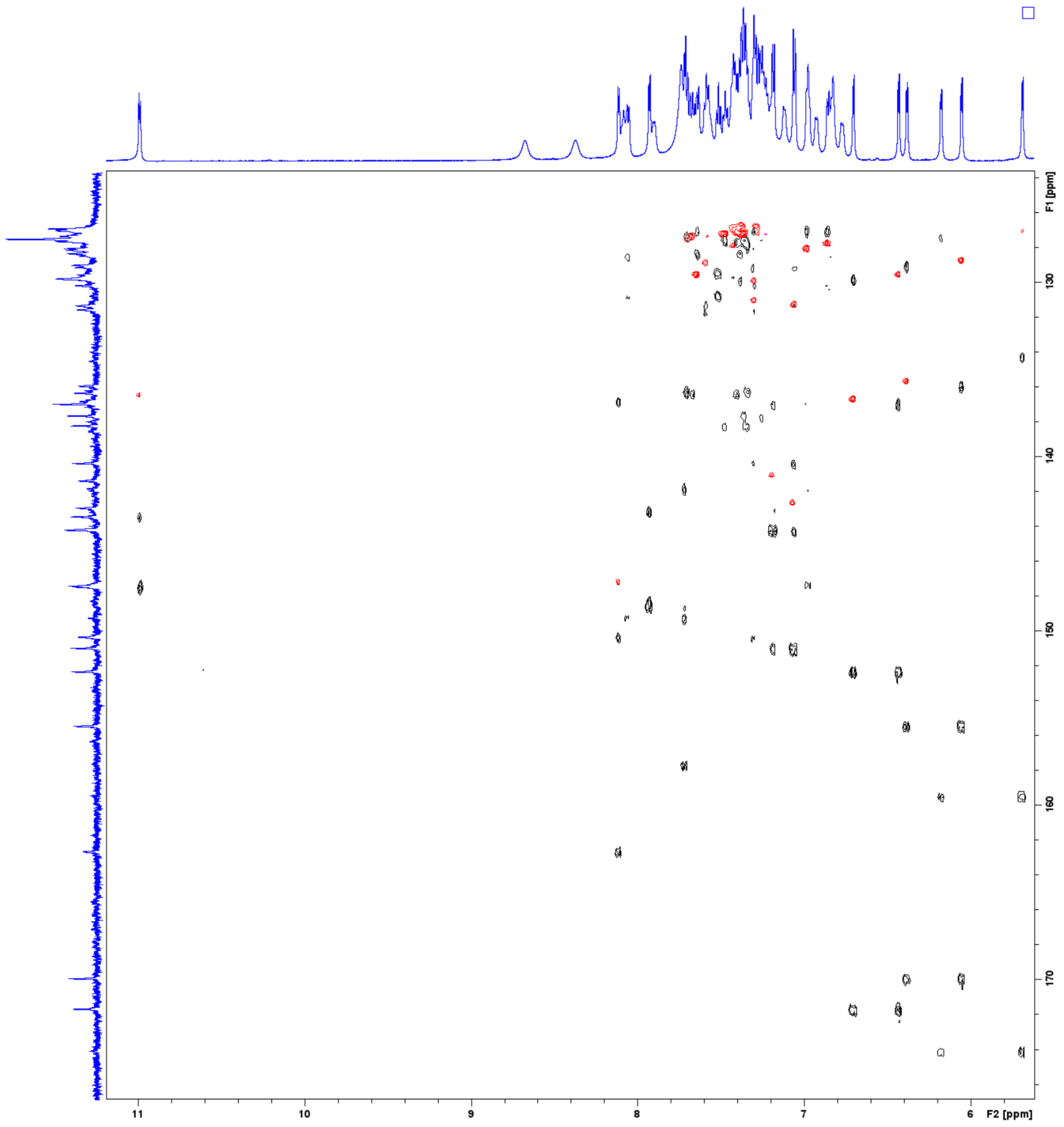

Figure S9. **^{1}H-^{13}C HSQC (red correlations) and ^{1}H-^{13}C HMBC (black correlations) spectra of 3; 600 MHz, CD_2Cl_2, 300 K (selected range).**

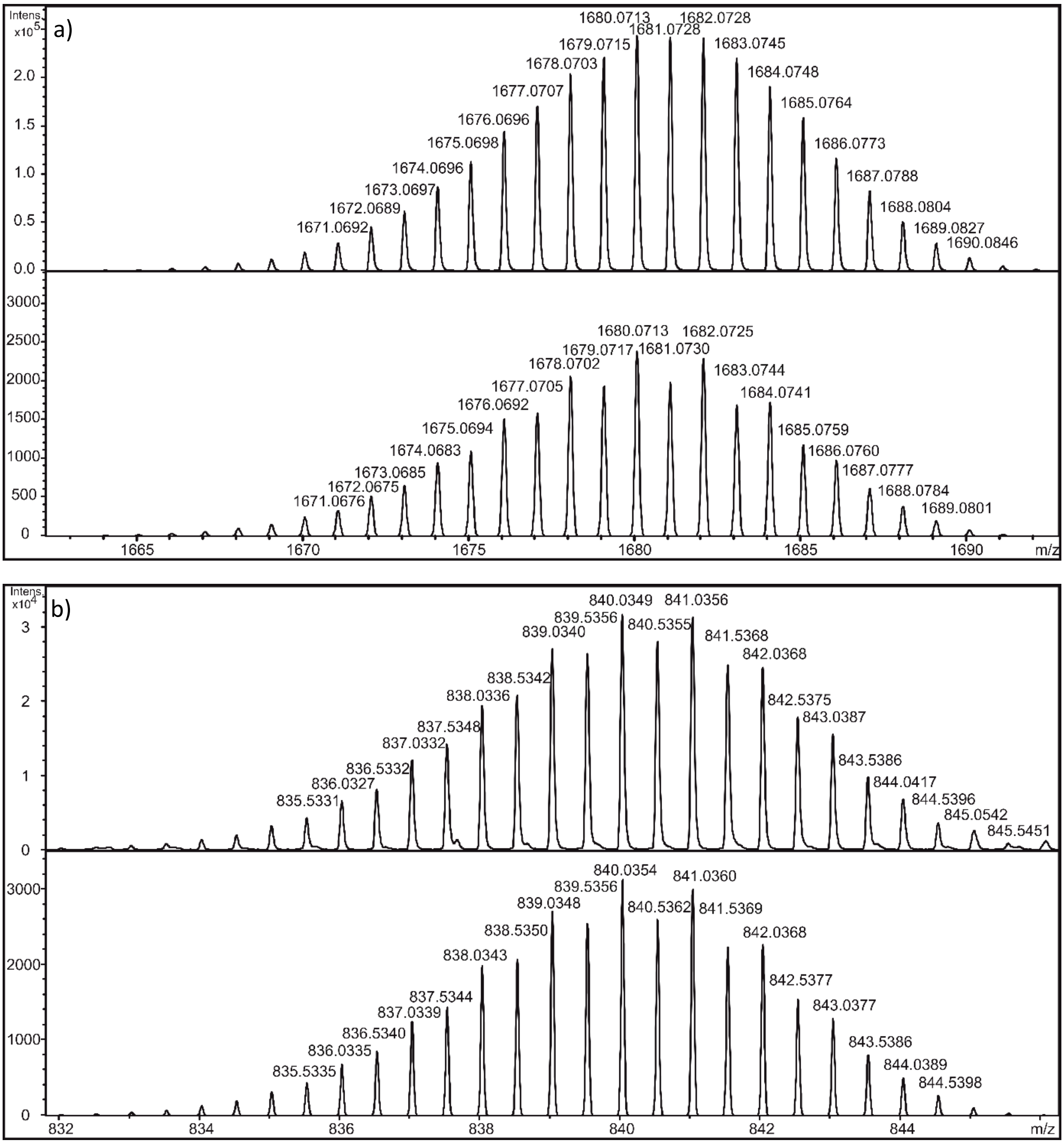

Figure S10. Selected regions of HRMS ESI (+MS) spectrum of 3: a) measured (top) and simulated (bottom) calc. for $C_{88}H_{56}N_4Te_4$, $[M]^+$, b) measured (top) and simulated (bottom) calc. for $C_{88}H_{56}N_4Te_4$, $[M]^{2+}$.

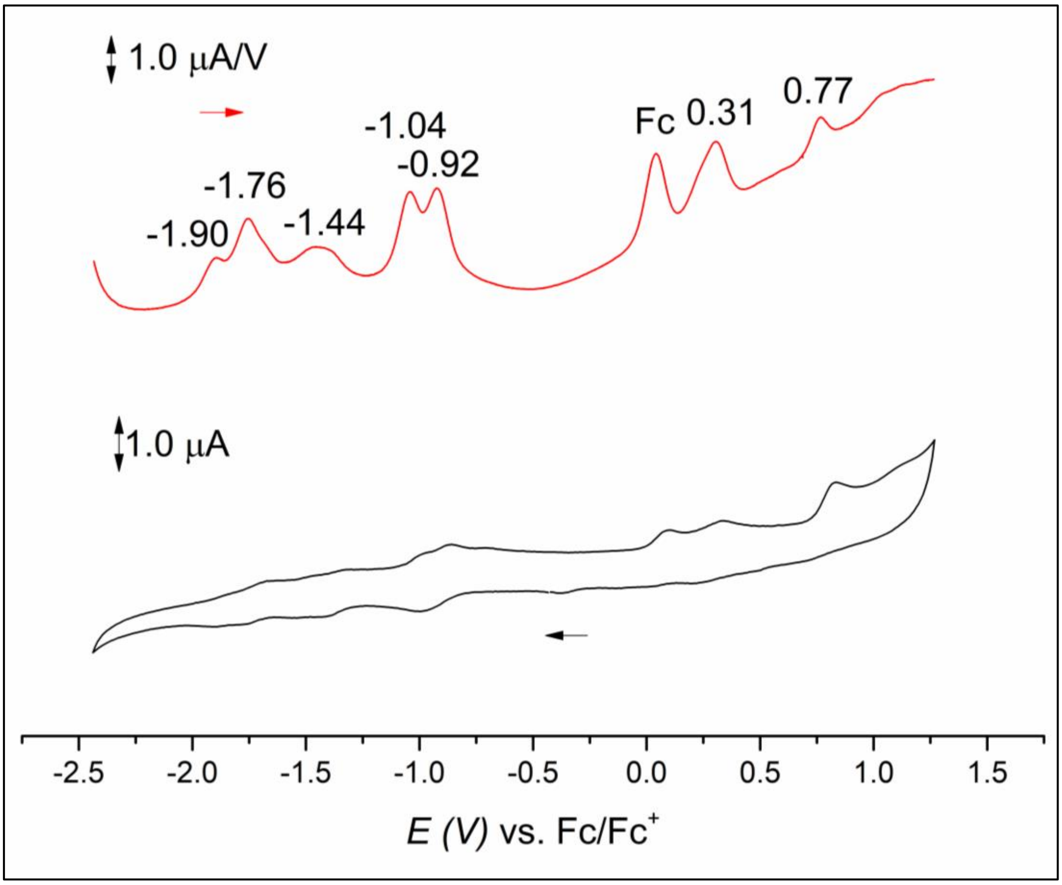

Figure S11. Cyclic (CV) and differential pulse (DP) voltammograms recorded for 3 in CH_2Cl_2 with $[Bu_4N]PF_6$ as supporting electrolyte. The horizontal arrows indicate directions of the potential sweep, the numbers are DP peak potentials in volts. The electrochemical HOMO-LUMO gap is 1.23 V.

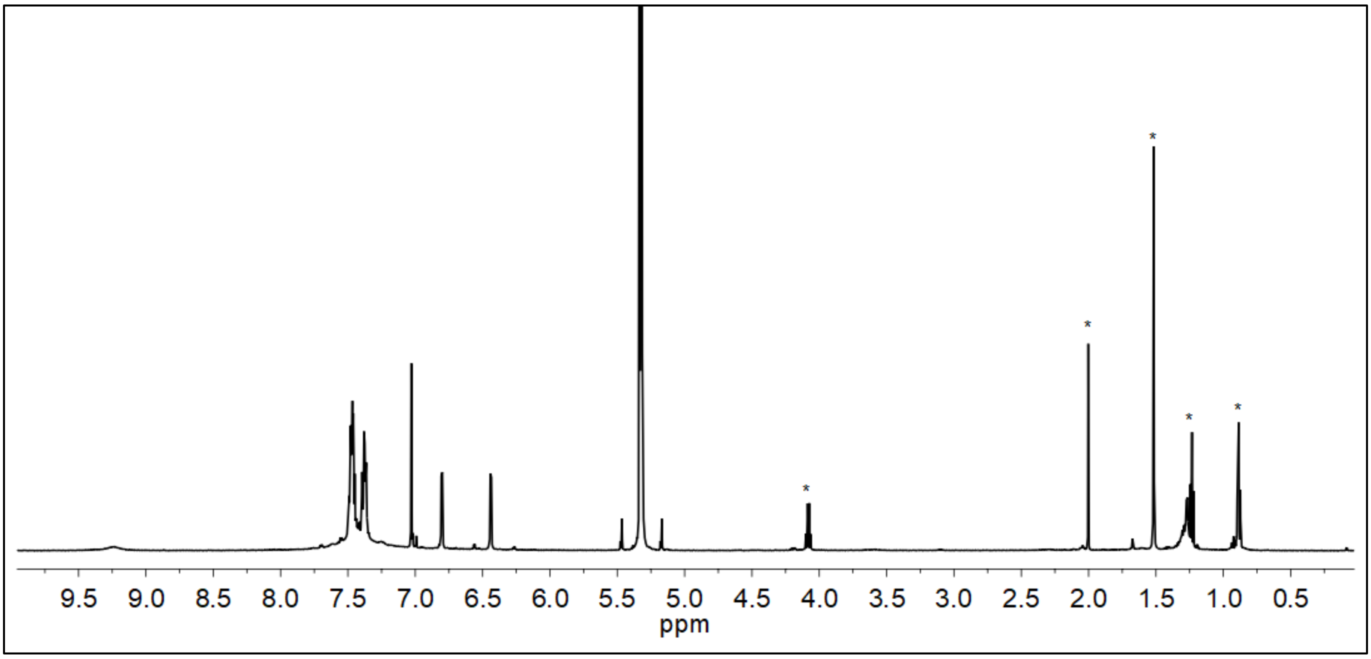

Figure S12. **^{1}H NMR spectrum of 3-Cl_4 (600 MHz, CD_2Cl_2, 300 K); * = H_2O, ethyl acetate, n-hexane. Signals of traces of 3 are visible.**

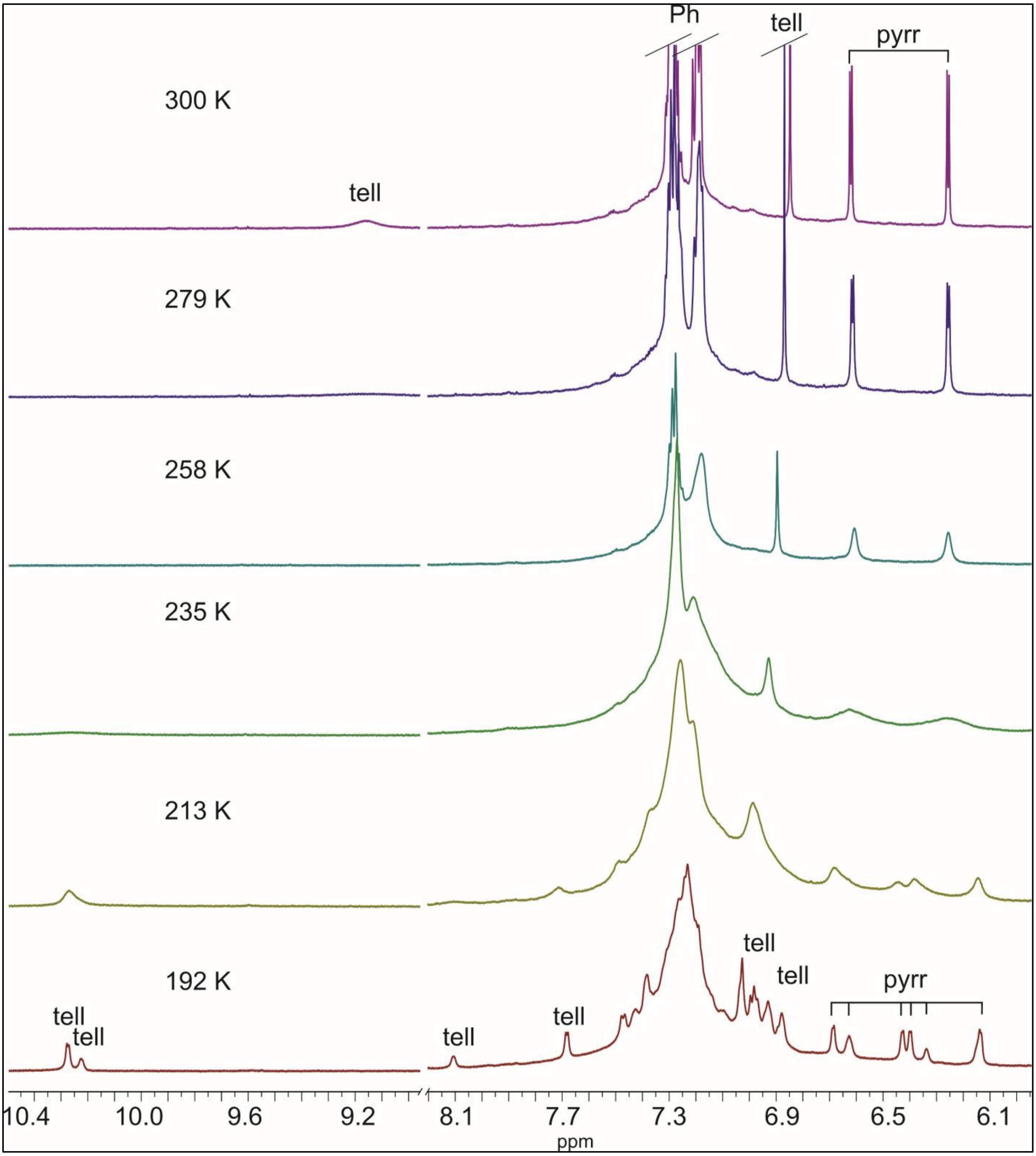

Figure S13. Variable temperature ^{1}H NMR spectra (selected range) of 3-Cl_4; 600 MHz, CD_2Cl_2, 300-192 K.

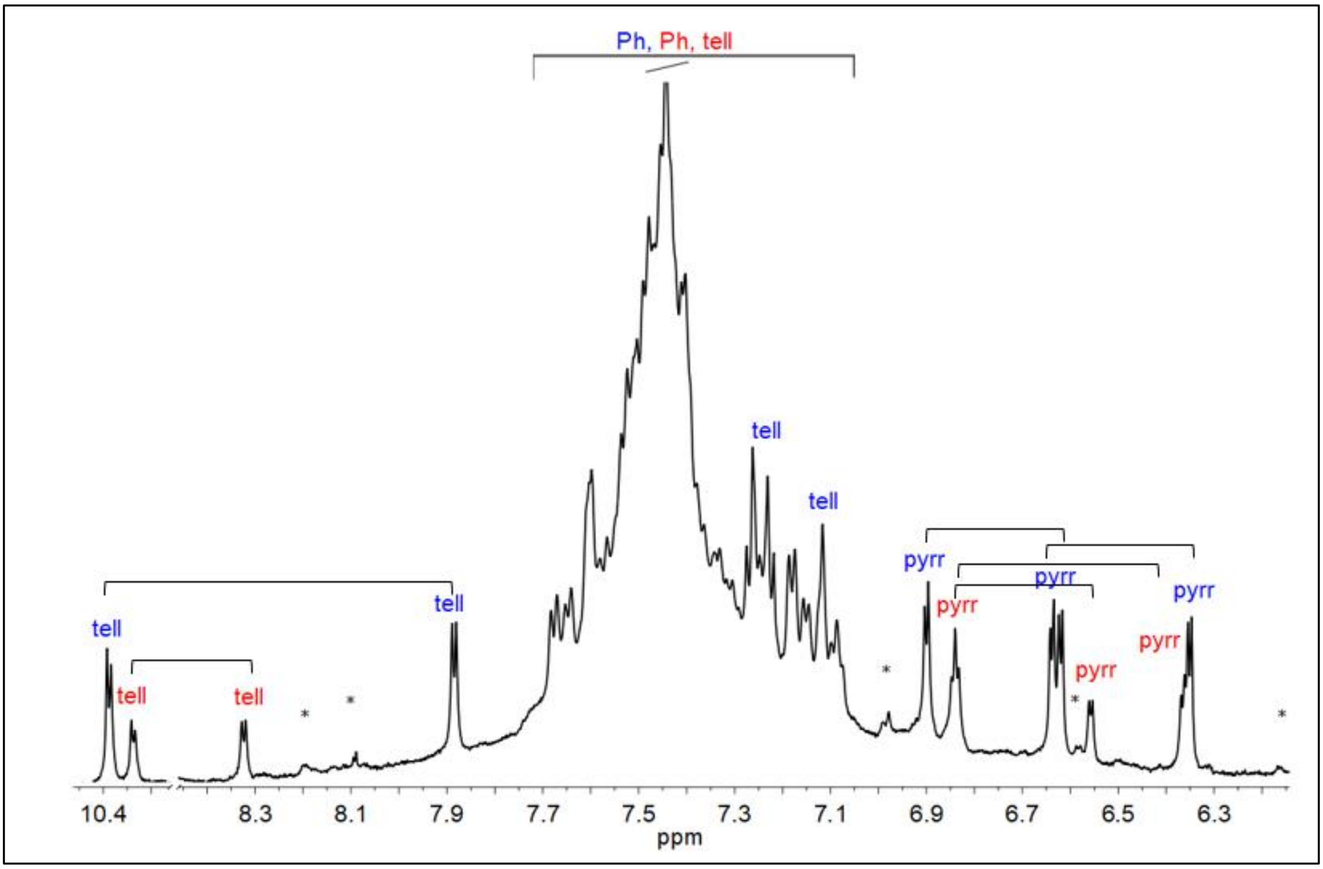

Figure S14. **^{1}H NMR spectrum (600 MHz, CD_2Cl_2, 182 K) of 3-Cl_4 (selected range). Two forms are denoted by different colors.**

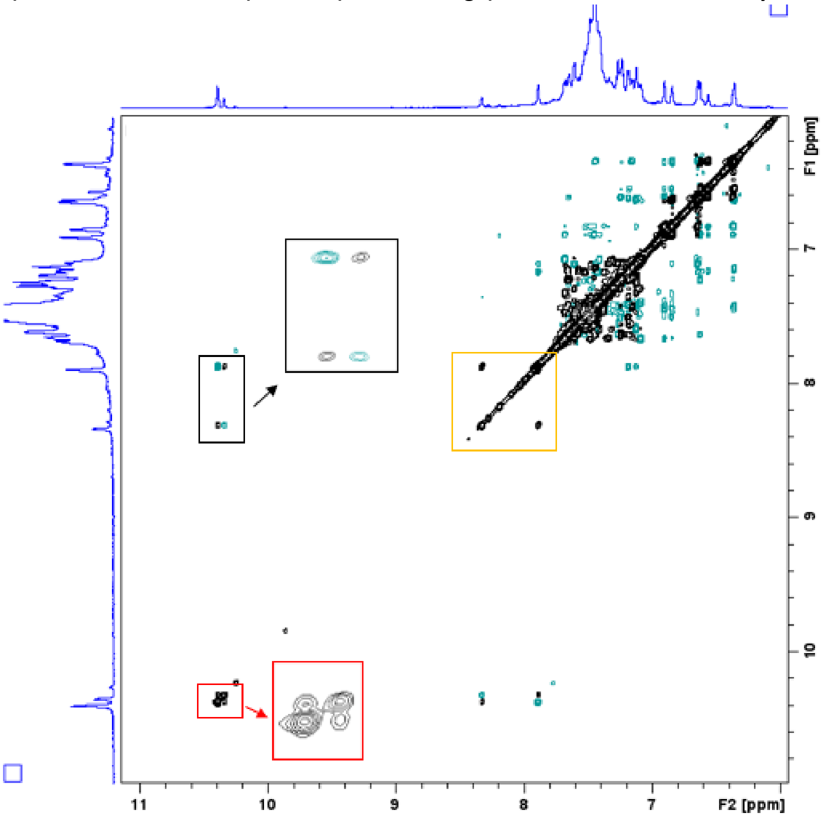

Figure S15. **^{1}H-^{1}H NOESY spectrum (600 MHz, CD_2Cl_2, 192 K) of 3-Cl_2 (selected range).**

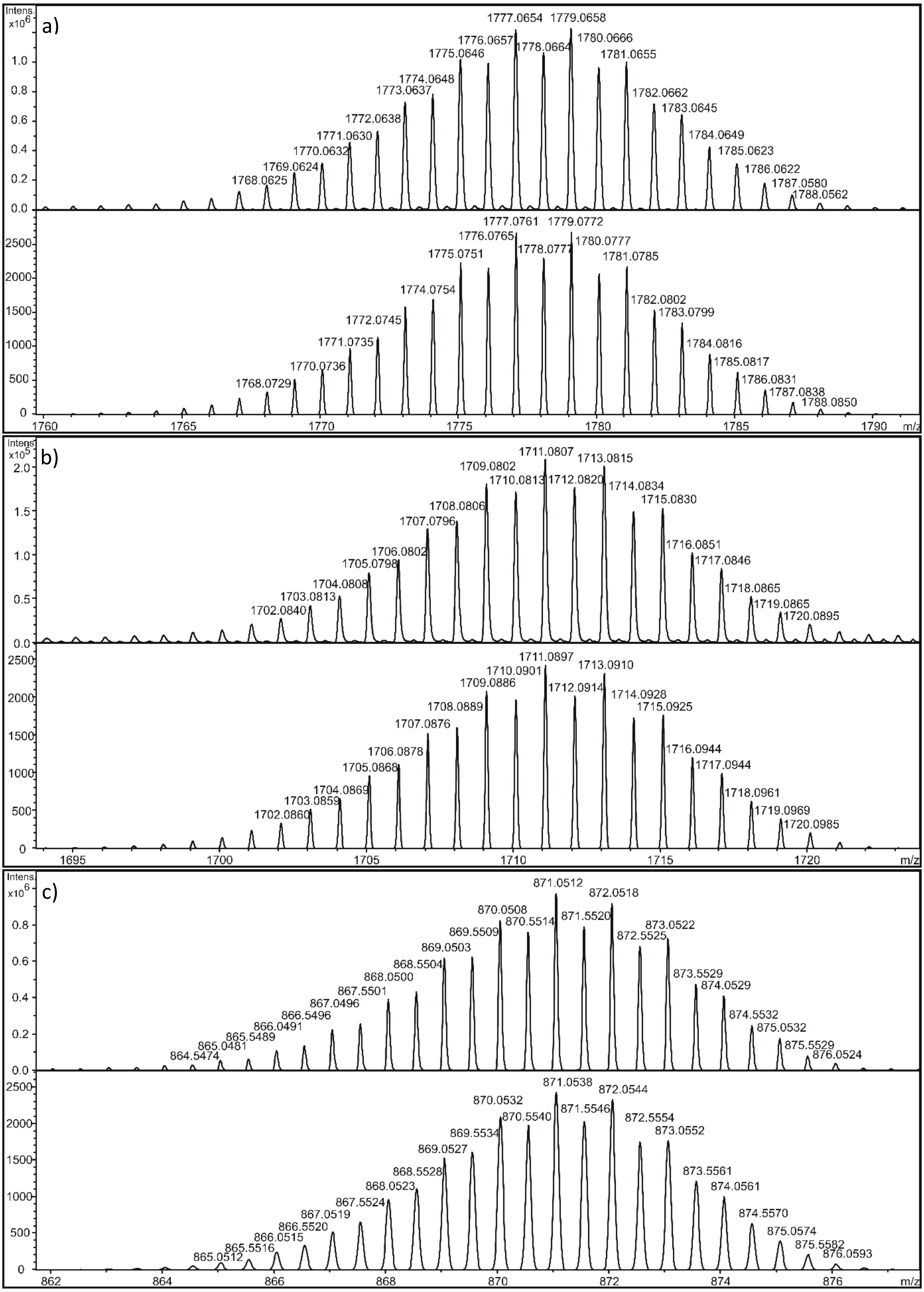

Figure S16. Selected regions of HRMS ESI (+MS) spectrum 3-Cl$_4$ obtained in CH_3OH: a): measured (top) and simulated (bottom) calc. for $C_{88}H_{56}N_4Te_4Cl(OCH_3)_2$, $[M\text{-}3Cl\text{+}2OCH_3]^+$, b): measured (top) and simulated (bottom) calc. for $C_{88}H_{56}N_4Te_4(OCH_3)_2$, $[M\text{-}4Cl\text{+}OCH_3]^+$, c) measured (top) and simulated (bottom) calc. for $C_{88}H_{56}N_4Te_4(OCH_3)_2$, $[M\text{-}4Cl\text{+}2OCH_3]^{2+}$.

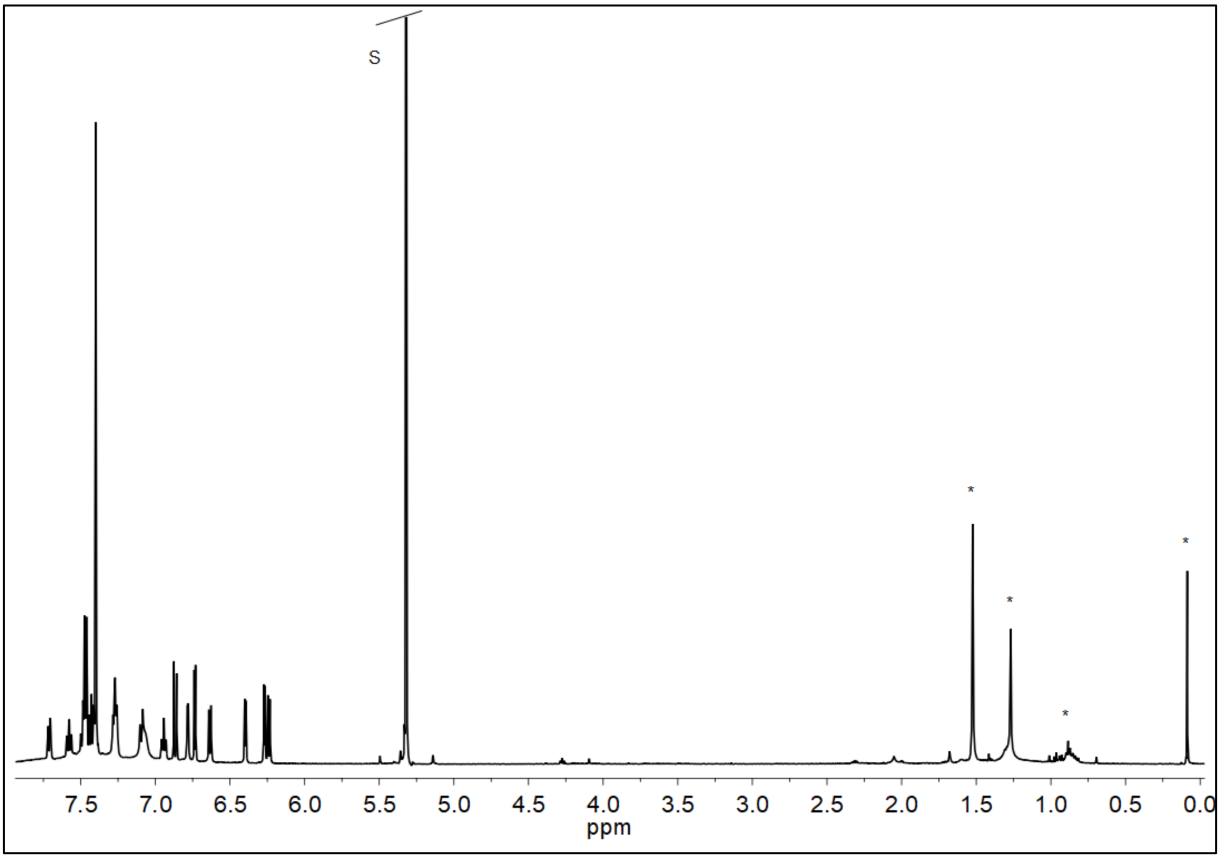

Figure S17. ^{1}H **NMR spectrum of 4 (600 MHz, CD_2Cl_2, 300 K); * = H_2O and impurities.**

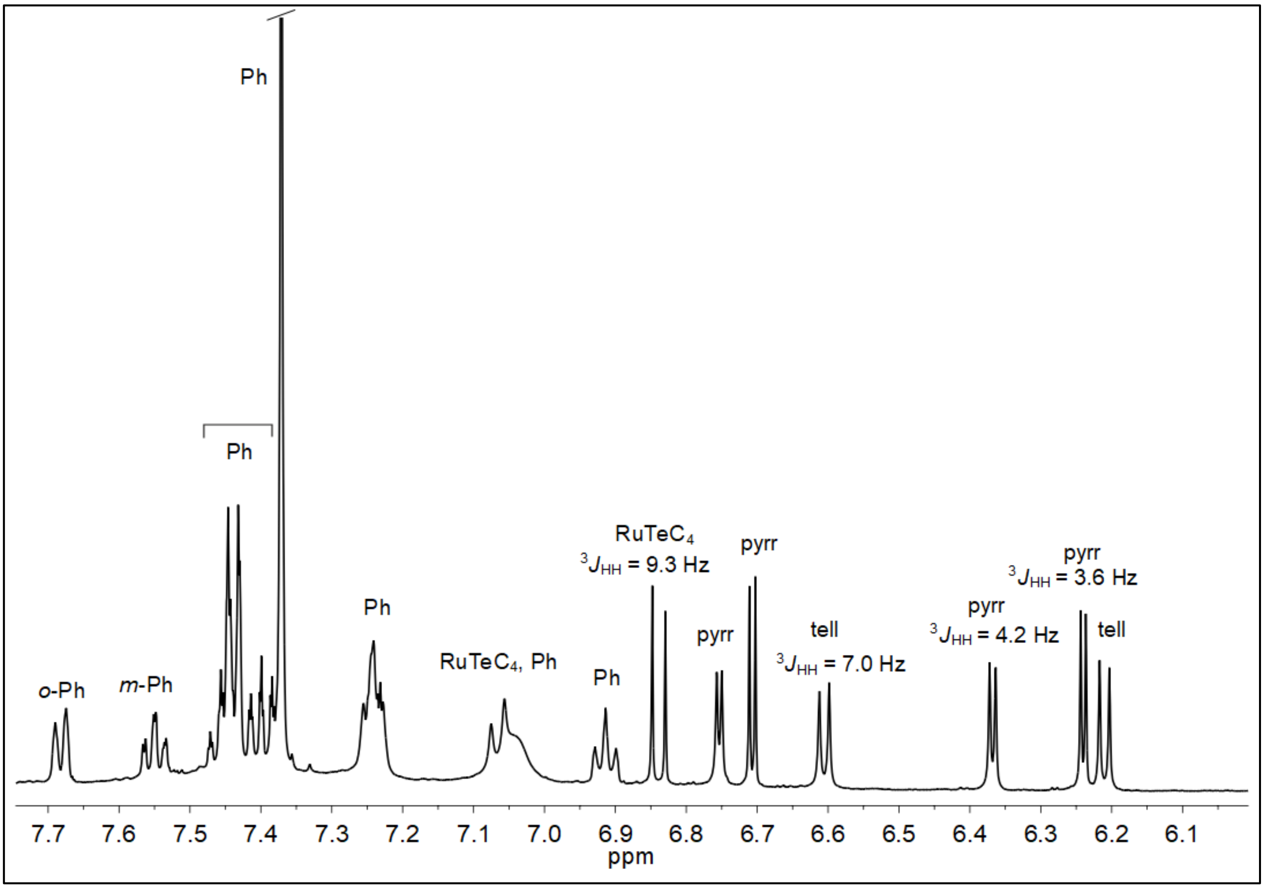

Figure S18. ^{1}H **NMR spectrum (600 MHz, CD_2Cl_2, 300 K) of 4 (selected range).**

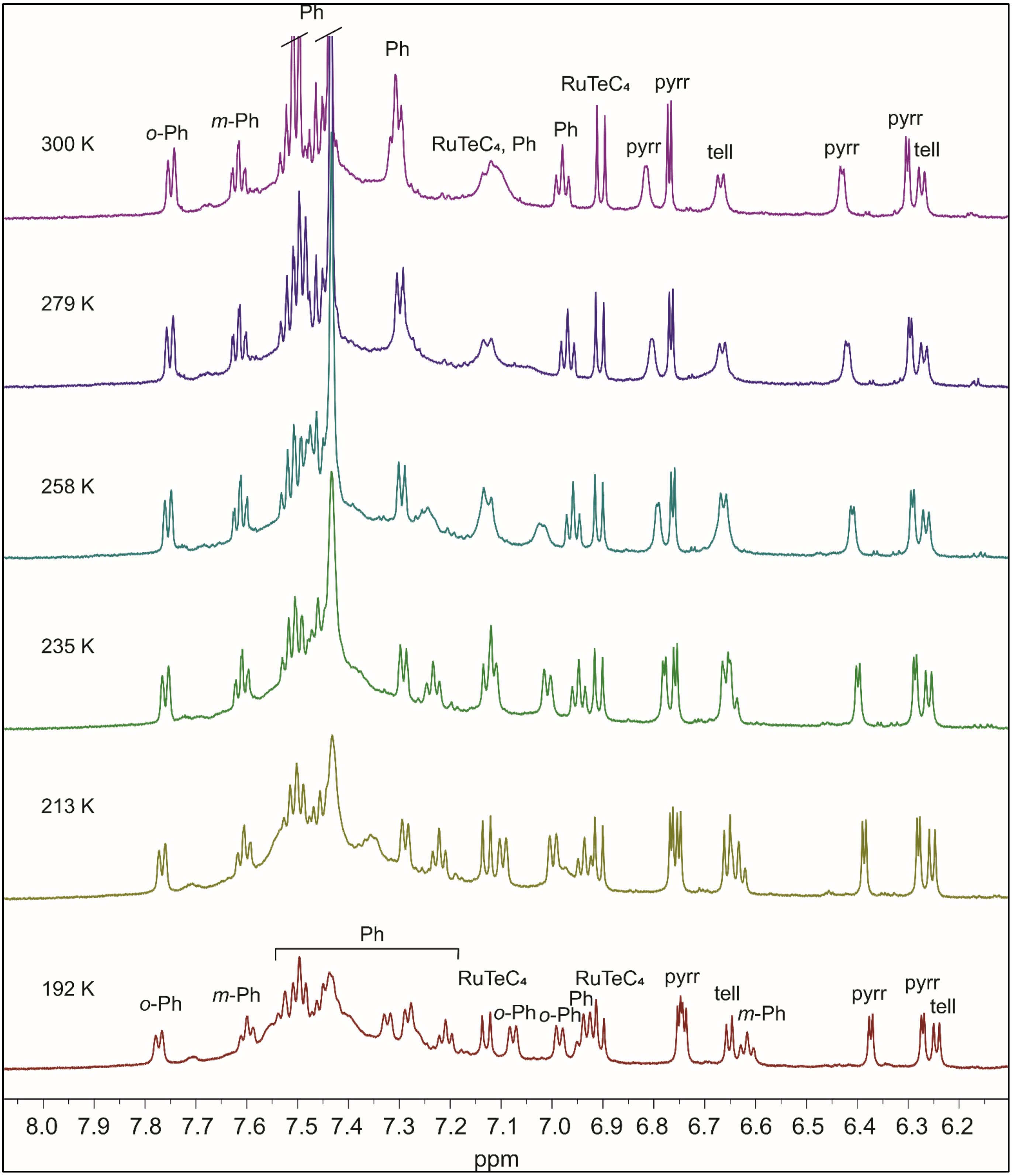

Figure S19. Variable temperature ^{1}H NMR spectra (600 MHz, CD_2Cl_2, 192-300 K) of 4 (selected range).

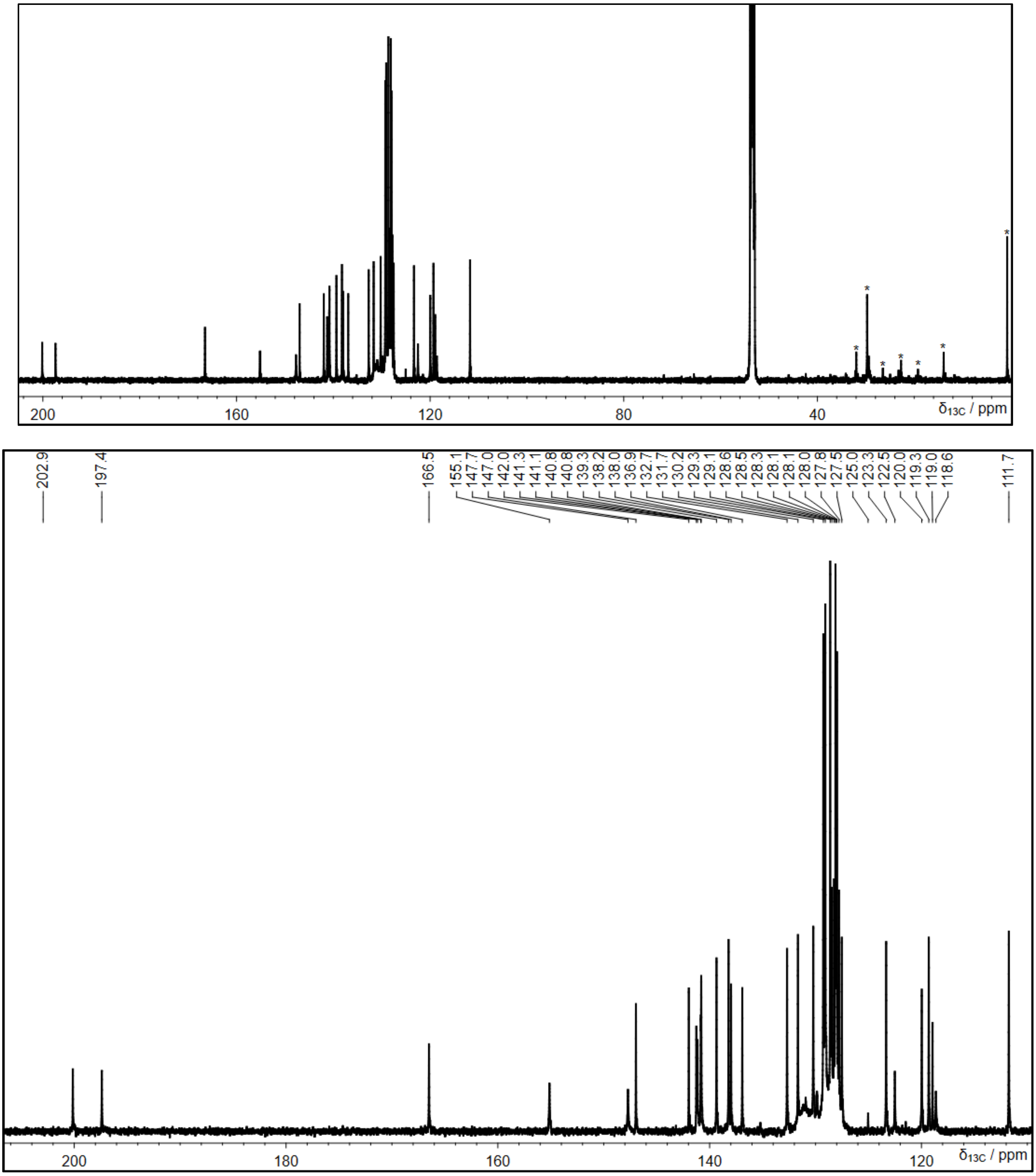

Figure S20. **^{13}C NMR (150 MHz, CD_2Cl_2, 300 K) spectrum of 4; (top: the whole spectral range, bottom: the most informative region; * = n-hexane and impurities).**

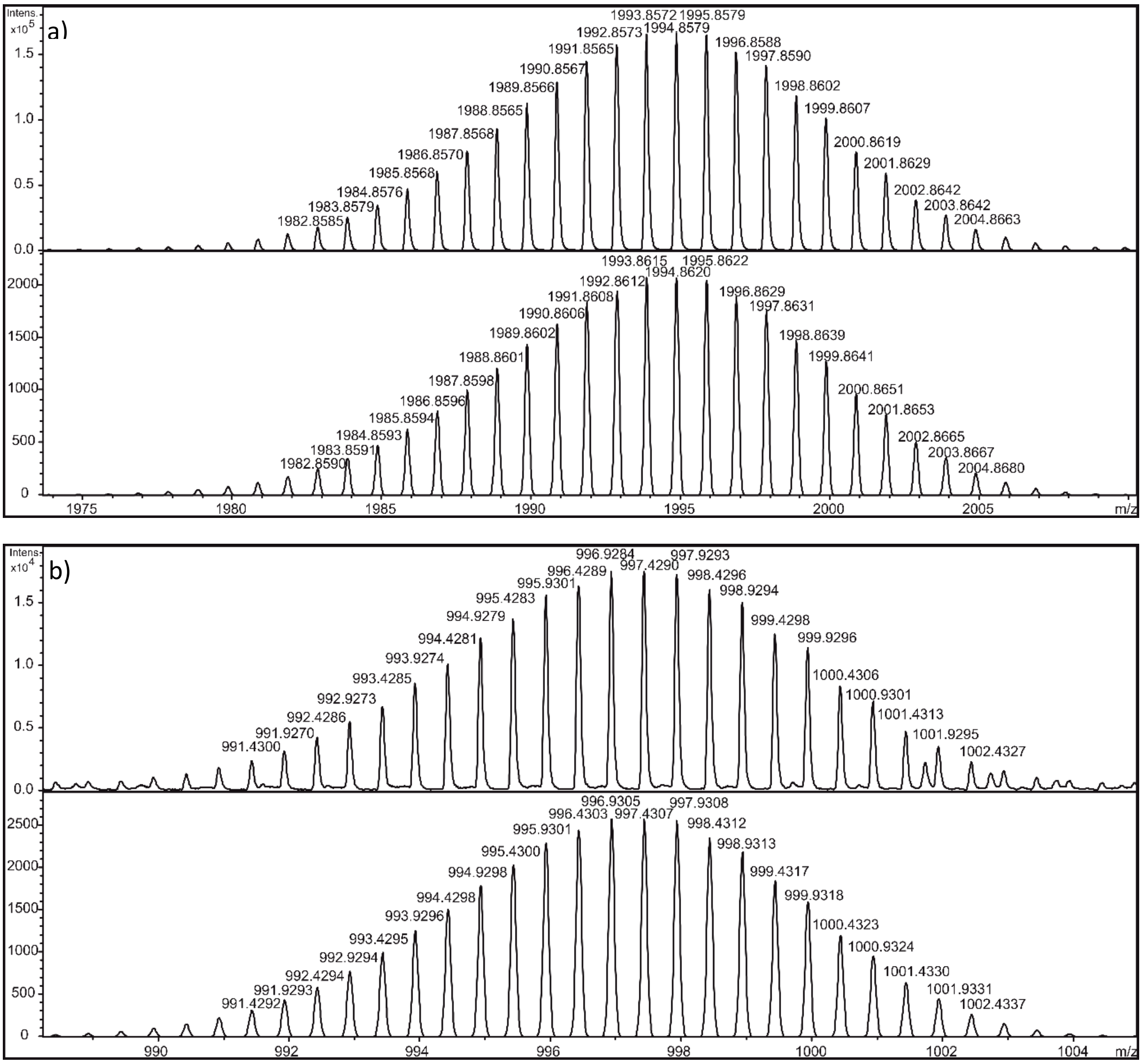

Figure S21. Selected regions of HRMS ESI (+MS) spectrum of 4: a) measured (top) and simulated (bottom) calc. for $C_{88}H_{56}N_4Te_4Ru_2$, $[M]^+$, b) measured (top) and simulated (bottom) calc. for $C_{88}H_{56}N_4Te_4Ru_2$, $[M]^{2+}$.

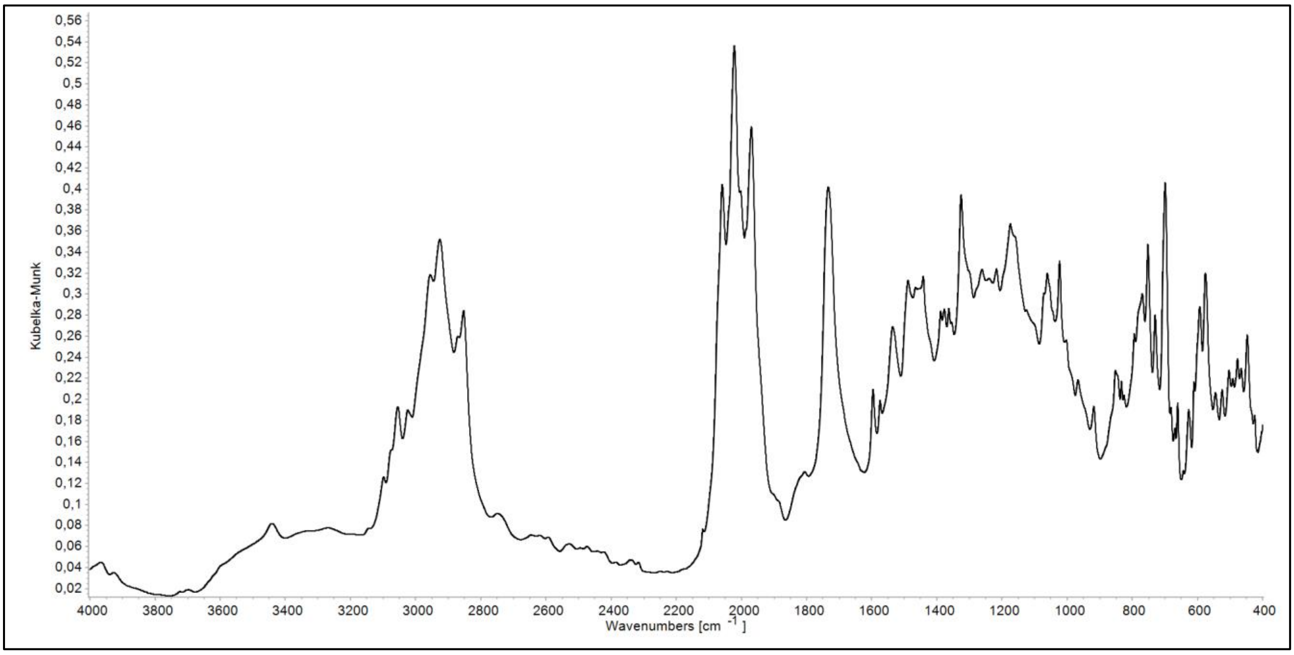

Figure S22. Diffuse reflectance infrared Fourier transform (DRIFT) spectrum of 4 (4 was diluted in KBr).

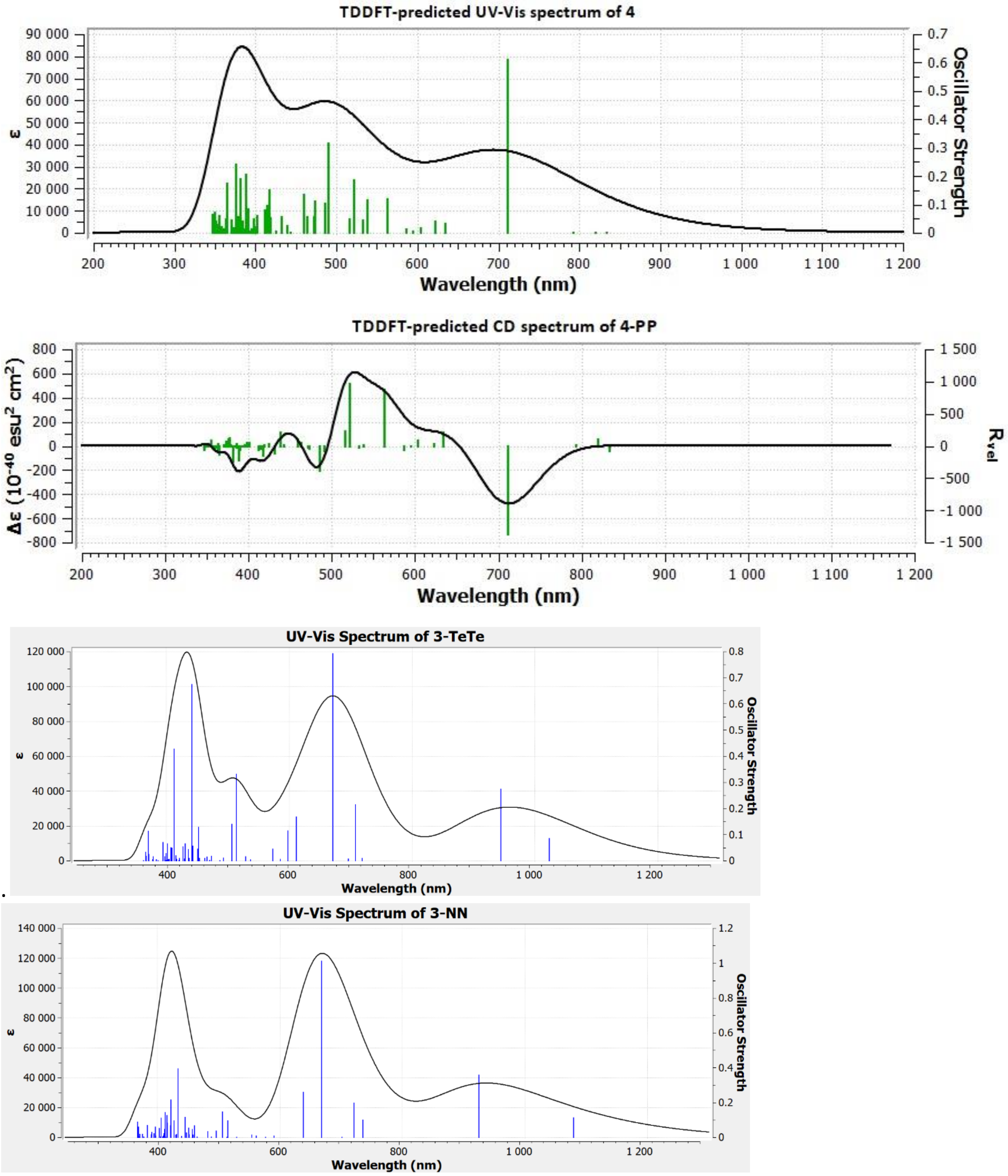

Figure S23. TDDFT-predicted UV-Vis and ECD spectra of 4 - *PP* enantiomer and TDDFT-predicted UV-Vis spectra of two conformers of 3.

X-ray crystallography

Single-crystal X-ray diffraction data were collected at 100 K on a Rigaku XtaLAB Synergy R, DW system (HyPix-Arc 150) (**3-NN**, **3-TeTe**, **3-Cl_4-p**, **4]**) κ-geometry four circle diffractometer using Cu *K*α radiation and on Agilent Technologies Xcalibur, Gemini ultra (ruby CCD detector) (**3-Cl_4-a**) κ-geometry four circle diffractometer using Mo *K*α radiation. Data reduction and analysis were carried out with the *CrysAlis* PRO programs[S4,S5]. Analytical absorption correction was applied to the data. The structures were solved by direct methods and refined with the full matrix least-squares technique using programs SHELXT[S6] and SHELXL-2014/7[S7]. Anisotropic displacement parameters were refined for all non-H atoms. H atoms were placed at calculated positions. The position of coordinated chlorine atoms (Cl49 and Cl50) in **3-Cl_4-a** and **3-Cl4-p** were found to be not fully occupied and were finally refined with site occupation factor SOF = 0.850 in **3-Cl_4-a** and SOF = 0.800 in **3-Cl_4-p**. Displacement ellipsoids clearly indicate the slightly disordered arrangement of one of the phenyl rings in **3-NN**, **3-Cl4-a** and **3-Cl4-p** and three of the phenyl rings in **4,** which were modelled by different (two) positions in the asymmetric unit. They were refined anisotropically with the site occupation factors (SOFs) = 0.628, 0.372 in **3-NN**, 0.524, 0.476 in **3-Cl-4-a**, 0.680, 0.320 in **3-Cl4-p** and 0.643, 0.357, 0.674, 0.326 and 0.706, 0.294 in **4** and SIMU or ISOR instructions were applied during the refinement process with SHELXL-2014 in **3-Cl4-p** and **3-Cl4-a** and **4**. Most of the solvent molecules in **3NN**, **3-TeTe**, **3-Cl4-a**, **3-Cl4-p** and **4** are highly disordered or partially occupied and some of the geometrical restraints (DFIX, SADI instructions in SHELXL-2014) and restraints on anisotropic displacement parameters (EADP, SIMU, ISOR) were applied in the refinement procedure if appropriate (see details in the CIF files). Details of the conditions for the data collection and the structures refinements are given in Table S3 and the crystallographic information files (CIFs) deposited with The Cambridge Crystallographic Data Centre (www.ccdc.cam.ac.uk/; deposition numbers 2376409-2376413) are provided as Supplementary Information.

Table S3. Crystal data for 3-NN, 3-TeTe, 3-Cl$_4$-a, 3-Cl$_4$-p, and 4.

	**3-NN**	**3-TeTe**	**3-Cl4-a**	**3-Cl4-p**	**4**
**Chemical formula**	$C_{88}H_{56}N_4Te_4$·3.98(C_2H_3N)·0.3($CHCl_3$)	$C_{88}H_{56}N_4Te_4$·0.9($CHCl_3$)	$C_{88}H_{56}Cl_{3.40}N_4Te_4$·$C_6H_{14}$·1.2($CHCl_3$)·0.4($CH_2Cl_2$)	$C_{93.10}H_{63.10}Cl_{6.40}N_{5.90}Te_4$	$C_{92}H_{56}N_4O_4Ru_2Te_4$·$C_4H_8O_2$·0.9($C_3H_8O$)
Mr	1879.79	1794.06	2063.68	2001.67	2136.13
**Crystal system, space group**	Monoclinic, $P2_1/c$	Monoclinic, $C2/c$	Monoclinic, $C2/c$	Triclinic, $P\bar{1}$	Monoclinic, $P2_1/c$
**Temperature (K)**	100	100	100	100	100
***a, b, c* (Å)**	24.172(4), 18.142(3), 19.690(3)	29.676(2), 13.112(6), 37.779(3)	35.746(1), 14.049(4), 18.438(5)	10.587(8), 19.263(16), 20.915(17)	20.286(1), 24.515(1), 19.946(1)
***α, β, γ* (°)**	90.00, 97.36(2), 90.00	90.00, 98.62(1), 90.00	90.00, 91.19(2), 90.00	99.61(2), 90.19(2), 97.74(2)	90.00, 113.60(1), 90.00
***V* (Å^3)**	8563 (2)	14534 (7)	9257(4)	4166(6)	9089(10)
Z	4	8	4	2	4
**Radiation type**	Cu *K*α	Cu *K*α	Mo *K*α	Cu *K*α	Cu *K*α
**μ (mm^{-1})**	11.29	13.91	1.52	13.22	13.06
**Crystal size (mm)**	0.22 × 0.10 × 0.01	0.07 × 0.01 × 0.01	0.24 × 0.16 × 0.12	0.17 × 0.10 × 0.04	0.32 × 0.05 × 0.03
***Dc* (g cm^{-3})**	1.458	1.640	1.481	1.596	1.561
**Diffractometer**	XtaLAB Synergy R, DW system, HyPix-Arc 150	XtaLAB Synergy R, DW system, HyPix-Arc 150	Xcalibur, Ruby, Gemini ultra		XtaLAB Synergy R, DW system, HyPix-Arc 150
**No. of measured, independent and observed [$I > 2\sigma(I)$] reflections**	56583, 16459, 10693	55441, 13077, 5969	17939, 8116, 5694	67041, 14803, 13924	56639, 15812, 10188
**R_{int}**	0.081	0.179	0.044	0.029	0.094
**$(\sin\vartheta/\lambda)_{max}$ (Å^{-1})**	0.621	0.615	0.595	0.597	0.592
**$R[F^2 > 2\sigma(F^2)]$, $wR(F^2)$, S**	0.078, 0.244, 1.06	0.087, 0.257, 0.98	0.058, 0.130, 1.06	0.032, 0.085, 1.03	0.077, 0.237, 1.05
**No. of reflections**	16459	13077	8116	14803	15812
**No. of parameters**	1084	898	644	1213	1228
**o. of restraints**	113	33	195	431	628
**$\Delta\rho_{max}$, $\Delta\rho_{min}$ (e Å^{-3})**	1.44, -1.57	1.46, -1.91	0.84, −1.03	0.83, −0.94	2.02, -1.16
**CCDC number**	2376409	2376410	2376411	2376412	2376413

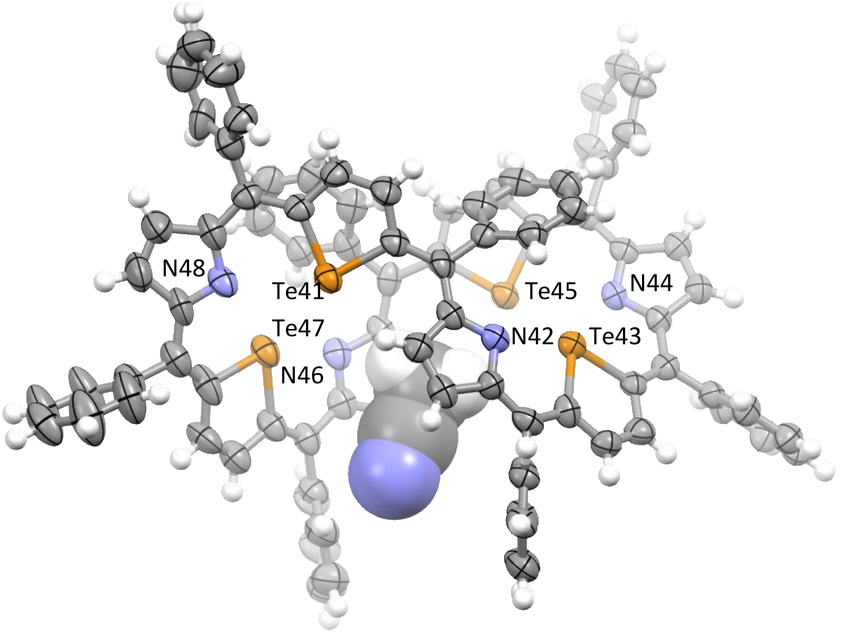

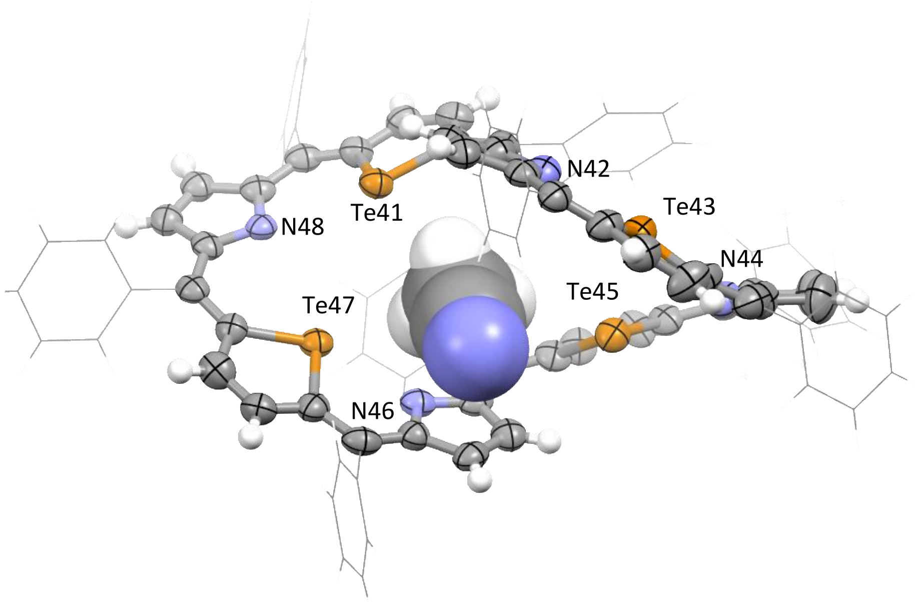

Figure S24. X-ray molecular structure of 3-NN·CH_3CN. Displacement ellipsoids represent 50% probability. In the side view (bottom), the aryl rings are shown as wireframes for clarity.

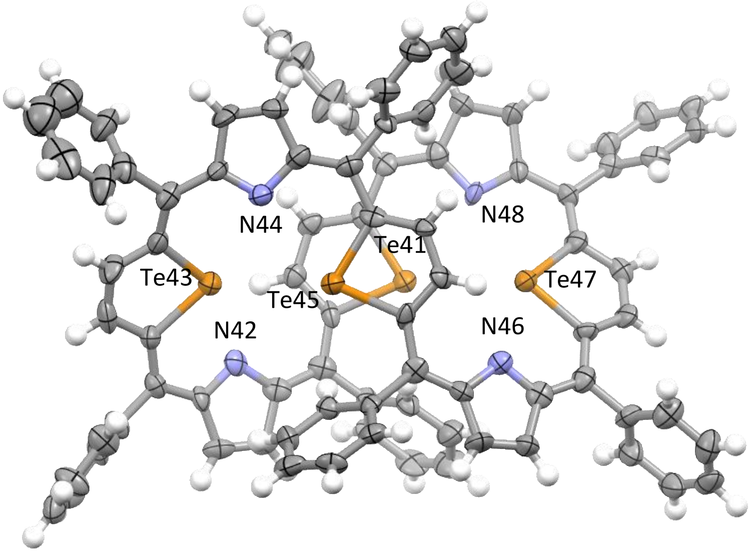

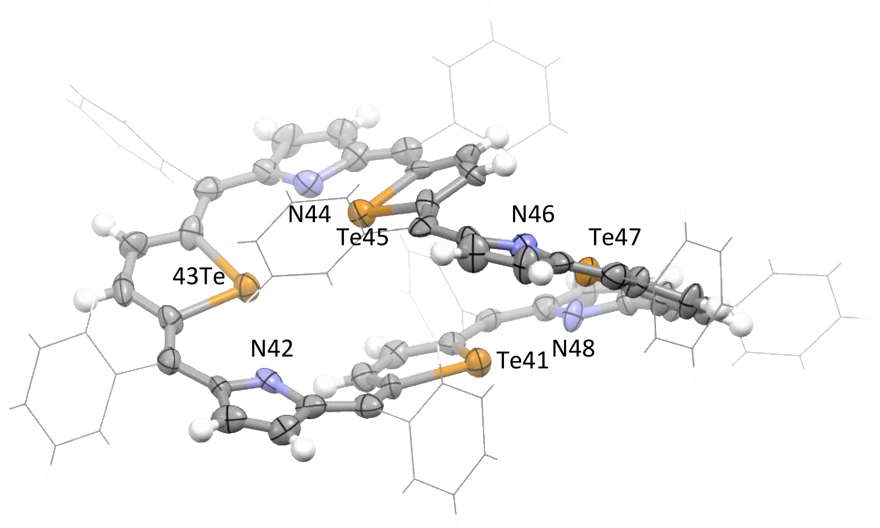

Figure S25. X-ray molecular structure of 3-TeTe. Displacement ellipsoids represent 50% probability. In the side view (bottom), the aryl rings are shown as wireframes for clarity.

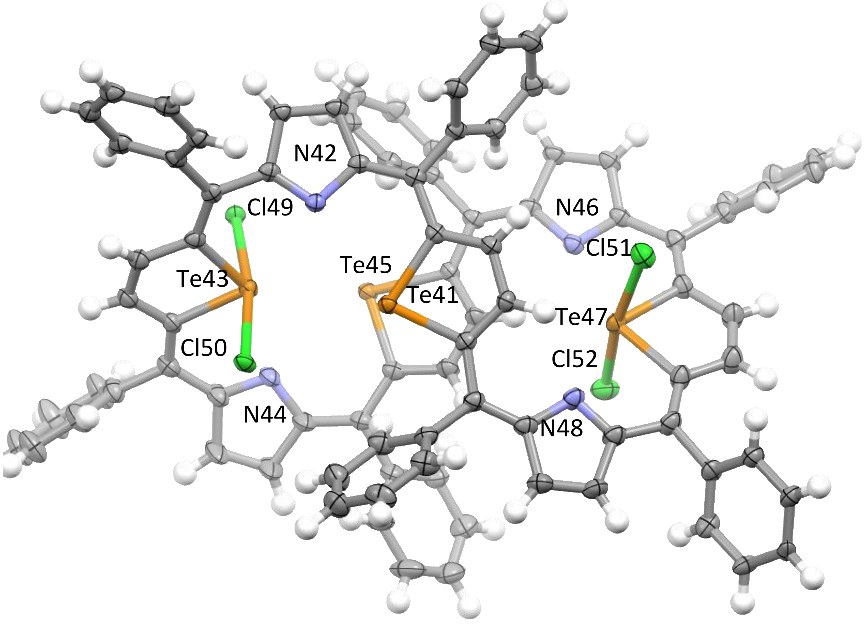

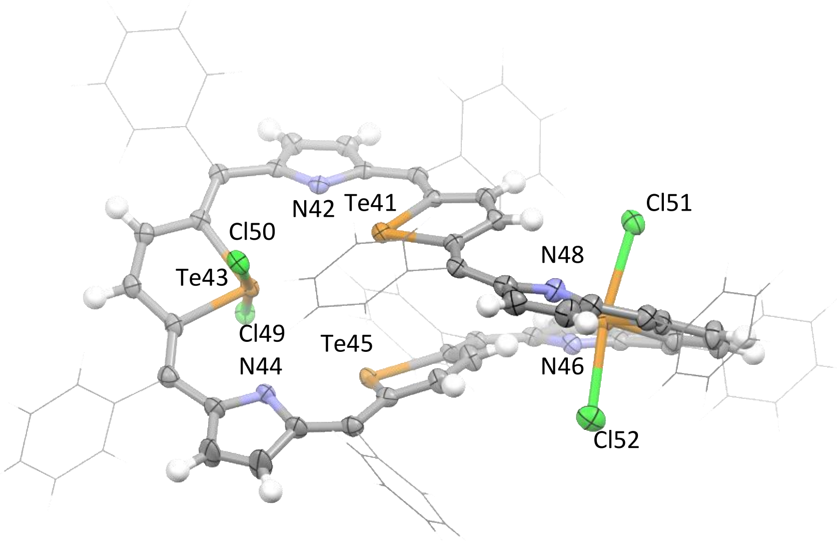

Figure S26. X-ray molecular structure of 3-Cl_4-p. Displacement ellipsoids represent 50% probability. In the side view (bottom), the aryl rings are shown as wireframes for clarity.

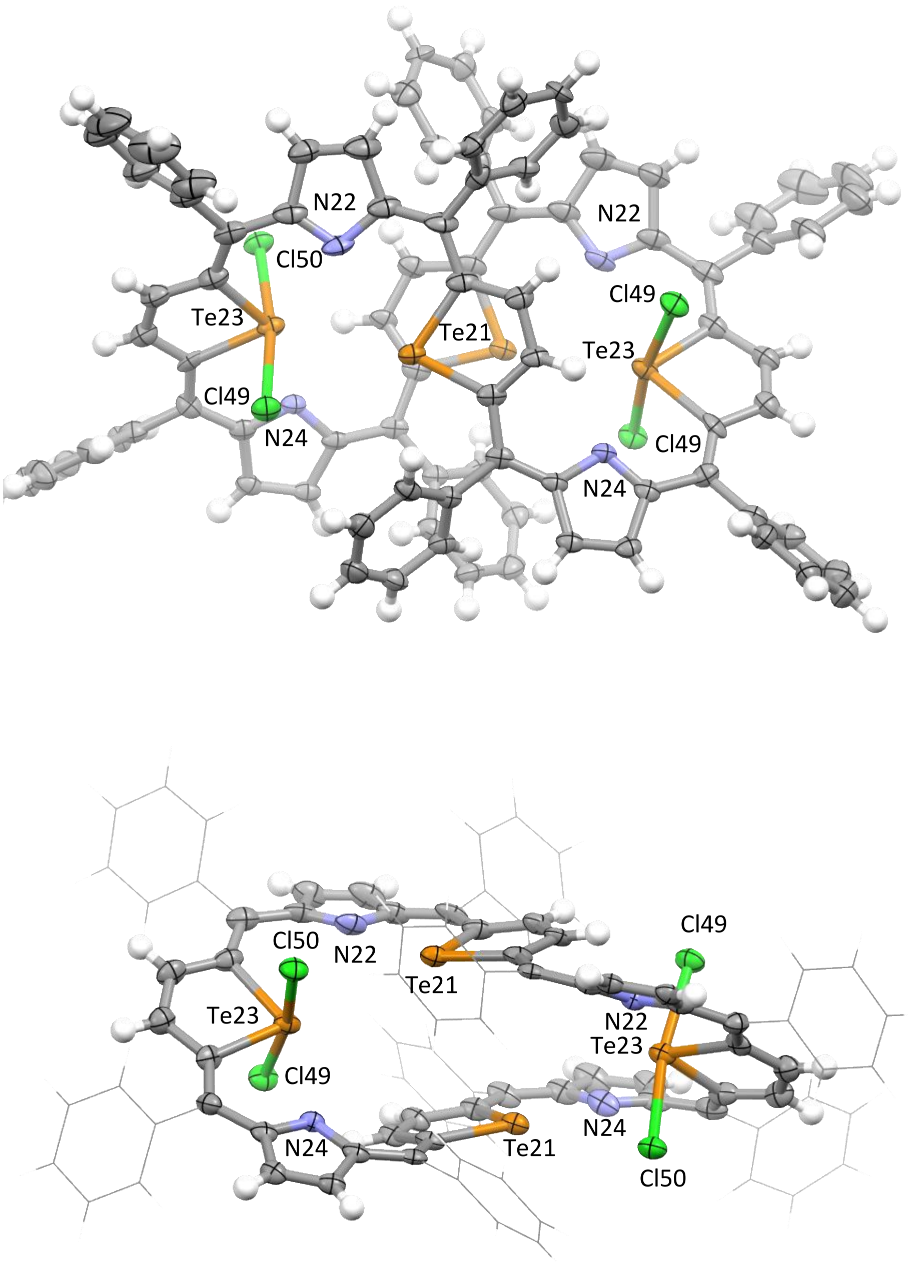

Figure S27. X-ray molecular structure of 3-Cl_4-a. Displacement ellipsoids represent 50% probability. In the side view (bottom), the aryl rings are shown as wireframes for clarity.

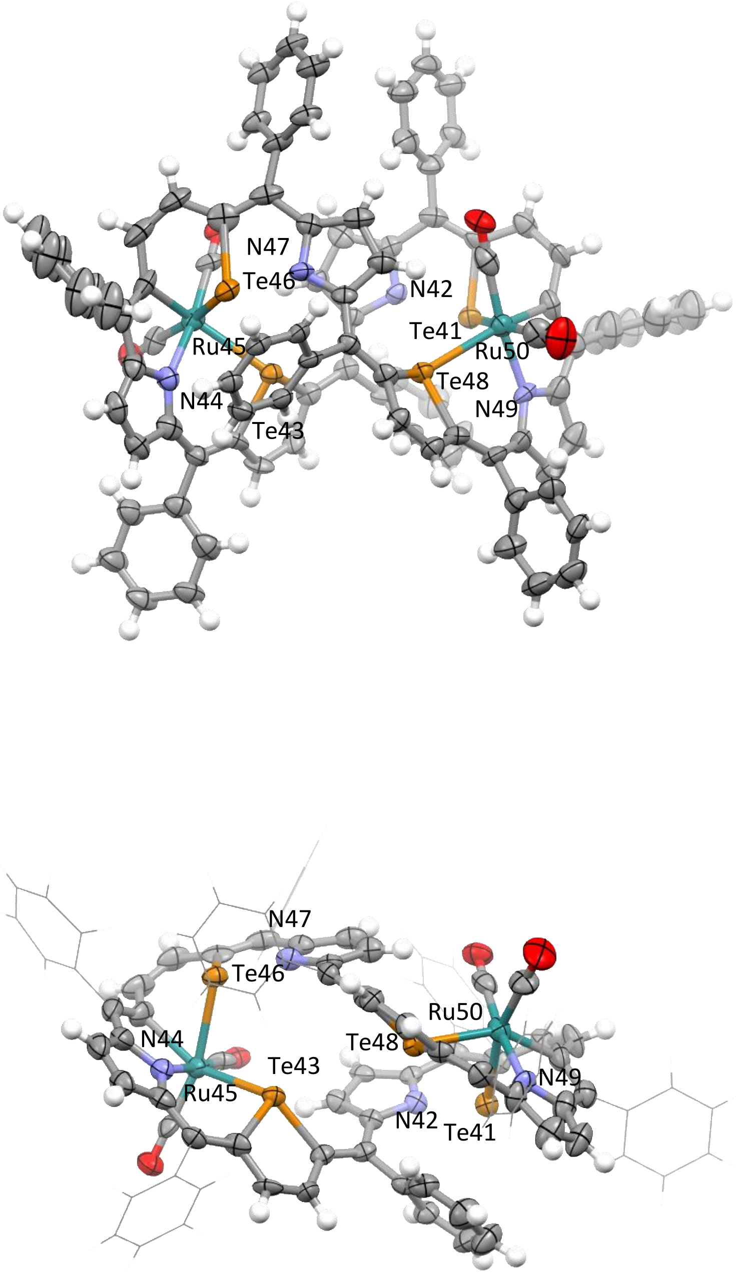

Figure S28. X-ray molecular structure of 4. Displacement ellipsoids represent 50% probability. In the side view (bottom), the aryl rings are shown as wireframes for clarity.

Table S4. Cartesian coordinates of DFT optimized structures of 3-NN, 3-TeTe, $CH_3CN \supset$3-NN, 3-Cl_4-a, 3-Cl_4-p (B3PW91/SDD for Ru and Te, and 6-31G(d,p) for the rest of atoms).

Coordinates for **3-TeTe**

Center Number	Atomic Number	Coordinates (Angstroms) X	Y	Z
------	-----------	--------------	------------	-----------
1	52	-4.083152	-0.171789	-0.572844
2	52	-1.408401	-0.081283	2.375875
3	52	4.083135	-0.169903	0.573713
4	52	1.409099	-0.080641	-2.373920
5	7	-3.178279	2.129872	1.689393
6	6	-5.614895	1.947528	1.210605
7	6	-2.305387	3.188645	1.915902
8	6	0.386234	1.732705	-2.527488
9	6	-0.608717	-4.293762	2.592285
10	6	-0.976166	3.017053	2.293268
11	6	-0.386814	1.732709	2.528831
12	7	3.176992	2.131769	-1.686459
13	7	-3.131379	-2.103365	-2.390667
14	6	-0.761619	-2.239749	-3.085799
15	6	1.373263	0.207662	3.170831
16	6	-0.125258	4.230270	2.503870
17	6	-0.366800	-3.160927	3.390948
18	6	0.369031	-3.162017	-3.386063
19	6	-6.864391	2.624682	1.676508
20	6	-4.381640	2.675680	1.485717
21	6	-5.627328	-1.511897	-1.090035
22	6	0.974931	3.017403	-2.292652
23	6	3.771386	-4.228703	3.030875
24	6	-0.473577	-0.832893	-2.964676
25	6	-5.451113	-2.639935	-1.868258
26	6	-5.670209	0.750059	0.530196
27	7	3.132463	-2.103059	2.390187
28	6	-2.443293	-4.128442	-3.305013
29	6	0.123779	4.230140	-2.505362
30	6	-1.674692	-5.148437	2.870977
31	6	0.609243	-4.294875	-2.586880
32	6	-3.014772	4.452283	1.787444
33	6	0.763468	-2.238840	3.088503
34	6	0.474835	-0.832106	2.966664
35	6	-4.316473	4.133978	1.554888
36	6	-6.570693	-3.599950	-2.106789
37	6	4.162953	-2.952933	2.436727
38	6	1.675865	-5.149470	-2.863254
39	6	3.012902	4.453979	-1.788676
40	6	2.444845	-4.128008	3.305252
41	6	0.924999	1.523429	2.958453
42	6	5.613860	1.950588	-1.209334
43	6	6.859257	-1.111062	0.463093
44	6	6.863470	2.628122	-1.674447
45	6	7.001638	-3.884365	3.407285
46	6	5.451166	-2.640496	1.864235
47	6	-0.925241	1.522408	-2.957781
48	6	-2.513435	-4.893229	3.957766
49	6	-2.036373	-2.780496	-2.914285
50	6	-3.770406	-4.228457	-3.033068
51	6	2.037907	-2.779771	2.915432
52	6	8.665165	-5.428391	2.567223
53	6	-8.666419	-5.424121	-2.580037
54	6	5.627042	-1.511530	1.087234
55	6	-7.920257	2.908718	0.792608
56	6	-7.000988	-3.879540	-3.415248
57	6	2.303855	3.189914	-1.914677
58	6	-8.039422	-4.780995	-3.649428
59	6	0.401667	5.126198	-3.549776
60	6	0.975052	4.484181	1.668645
61	6	6.570269	-3.601749	2.099823
62	6	2.288921	-3.775417	-4.752658
63	6	-1.227103	-2.911060	4.476378
64	6	-6.994969	3.023510	3.018953
65	6	-9.072523	3.556516	1.238812
66	6	-8.149057	3.665517	3.466032
67	6	1.231566	-2.912215	-4.469748
68	6	8.039637	-4.787064	3.638564
69	6	8.243130	-5.161728	1.263457
70	6	-4.162292	-2.952678	-2.439186
71	6	-6.879487	-0.010654	0.340433
72	6	4.314781	4.136428	-1.556028
73	6	6.994809	3.027871	-3.016538
74	6	7.918958	2.911390	-0.789841
75	6	5.669453	0.752642	-0.529618
76	6	6.878729	-0.008510	-0.341672
77	6	7.204763	-4.258759	1.032066
78	6	-0.974802	4.486676	-1.668800
79	6	4.380399	2.678261	-1.484430
80	6	-9.192720	3.934444	2.577488
81	6	2.516913	-4.894259	-3.948267
82	6	-1.372523	0.206293	-3.169786
83	6	1.480371	6.504133	2.902158
84	6	1.767872	5.614912	1.864508
85	6	-2.283784	-3.774331	4.761599
86	6	0.393201	6.257399	3.742301
87	6	-6.859880	-1.111779	-0.466221
88	6	-0.405003	5.129583	3.545020
89	6	-8.245496	-5.160422	-1.275307
90	6	-0.396799	6.253505	-3.748919
91	6	-7.206744	-4.258650	-1.041012
92	6	9.071575	3.559267	-1.234974
93	6	-1.482396	6.502851	-2.907497
94	6	8.149205	3.670090	-3.462543
95	6	9.192510	3.938158	-2.573311
96	6	-1.767996	5.616816	-1.866603
97	1	0.035594	-4.485114	1.740163
98	1	2.388257	0.006272	3.492275
99	1	4.420020	-5.077762	3.192980
100	1	-1.806048	-4.868583	-3.766990
101	1	-1.854109	-6.009978	2.234322
102	1	-0.036861	-4.486190	-1.736115
103	1	-2.577638	5.435732	1.882375
104	1	-5.151165	4.805639	1.411668
105	1	1.853976	-6.010986	-2.226203
106	1	2.575477	5.437117	-1.885552
107	1	1.807960	-4.867948	3.768024
108	1	1.588488	2.363783	3.132372
109	1	7.759317	-1.693642	0.631320
110	1	6.522038	-3.383206	4.243151
111	1	-1.589327	2.362155	-3.132270
112	1	-3.342700	-5.560153	4.175269
113	1	-4.419136	-5.077174	-3.196611
114	1	9.473111	-6.131723	2.747394
115	1	-9.474675	-6.126518	-2.762451
116	1	-7.824284	2.630291	-0.252408
117	1	-6.520196	-3.376992	-4.249595
118	1	-8.361555	-4.977838	-4.668210
119	1	1.240067	4.928792	-4.210851
120	1	1.202728	3.800412	0.857175
121	1	2.934555	-3.574049	-5.602566
122	1	-1.047837	-2.046402	5.107409
123	1	-6.186902	2.814886	3.713950
124	1	-9.873753	3.772285	0.537465
125	1	-8.233880	3.955121	4.509728

126	1	1.053637	-2.047563	-5.101165
127	1	8.362630	-4.986276	4.656612
128	1	8.717580	-5.662418	0.423988
129	1	-7.792381	0.285387	0.847123
130	1	5.149352	4.808583	-1.414455
131	1	6.187134	2.819804	-3.712156
132	1	7.822403	2.632162	0.254904
133	1	7.791369	0.288142	-0.848455
140	1	2.608831	5.794655	1.201344
141	1	-2.927604	-3.572969	5.612881
142	1	0.166188	6.940364	4.556059
143	1	-7.760101	-1.693697	-0.635929
144	1	-1.244576	4.934206	4.205194
145	1	-8.721166	-5.662467	-0.437341
146	1	-0.171246	6.934044	-4.565112
147	1	-6.871587	-4.067662	-0.025865
148	1	9.872493	3.774350	-0.533062
149	1	-2.102959	7.380660	-3.063230
150	1	8.234583	3.960459	-4.505982
151	1	10.090294	4.442009	-2.919824
152	1	-2.607739	5.798568	-1.202437
134	1	6.868618	-4.065512	0.017676
135	1	-1.200730	3.805363	-0.854834
136	1	-10.090244	4.438213	2.924795
137	1	3.346694	-5.561133	-4.163949
138	1	-2.387221	0.004118	-3.491610
139	1	2.100710	7.382365	3.056382

Coordinates for **3-NN**

Center	Atomic	Coordinates (Angstroms)		
Number	Number	X	Y	Z
------	-----------	--------------	------------	-----------
1	52	-2.871071	-0.756138	-1.927172
2	52	-3.847003	0.824018	1.625816
3	52	2.870104	-0.764004	1.923621
4	52	3.844606	0.830396	-1.623665
5	7	-5.499229	-0.769954	-0.678741
6	7	1.511748	0.792522	-3.109170
7	7	-1.513147	0.779963	3.111280
8	7	5.498772	-0.772565	0.677395
9	6	-5.068207	-2.964564	-1.677884
10	6	-1.342763	2.065338	3.431718
11	6	-7.793134	-0.952292	-0.475017
12	1	-8.807319	-0.654717	-0.249962
13	6	-5.634293	3.165471	1.423586
14	1	-6.447513	3.833190	1.157920
15	6	2.235852	-2.059147	-3.960983
16	1	2.118283	-1.364631	-4.785201
17	6	-4.611927	3.598224	2.218751
18	1	-4.606442	4.607078	2.619138
19	6	-0.084035	-1.197006	3.008690
20	6	-3.522157	2.708197	2.515993
21	6	-5.616563	1.812297	0.929787
22	6	1.265438	-2.084553	-2.943161
23	6	0.082934	-1.184502	-3.012953
24	6	2.803329	-3.576155	2.576251
25	1	3.071395	-4.620208	2.701337
26	6	-2.381574	3.044479	3.222688
27	6	1.341772	2.078764	-3.426084
28	6	3.720129	-2.666708	2.049689
29	6	-3.335169	-2.931520	3.905762
30	1	-4.071554	-2.904845	4.703698
31	6	-7.354980	-2.157495	-0.924143
32	1	-7.944542	-3.030163	-1.165162
33	6	0.025843	2.317462	3.872189
34	1	0.417473	3.263029	4.219842
35	6	-7.948366	1.961574	0.028077
36	6	2.539261	-3.835531	-1.830458
37	1	2.661661	-4.511999	-0.989803
38	6	-6.158464	-6.109307	-3.524156
39	1	-6.189817	-6.483784	-4.543348
40	6	3.331552	-2.917785	-3.920619
41	1	4.064744	-2.891681	-4.721506
42	6	-2.240452	-2.071773	3.949205
43	1	-2.126936	-1.375965	4.772848
44	6	-8.472563	2.132137	-1.265566
45	1	-7.917775	1.747692	-2.116522
46	6	5.069737	-2.969569	1.672004
47	6	-1.265714	-2.097775	2.935406
48	6	-0.026723	2.332507	-3.866006
49	1	-0.418001	3.279124	-4.211195
50	6	-9.681786	2.796978	-1.466195
51	1	-10.063716	2.926857	-2.474969
52	6	-2.153315	4.428715	3.732143
53	6	1.431658	-2.990713	-1.880809
54	1	0.697243	-3.012328	-1.082588
55	6	-6.607497	-0.113168	-0.313485
56	6	-6.654747	1.236961	0.226058
57	6	-0.281361	0.160312	3.266419
58	6	-2.533742	-3.851680	1.820288
59	1	-2.651919	-4.529839	0.980395
60	6	5.614586	1.815562	-0.923860
61	6	5.913913	-2.036491	1.076460
62	6	0.279884	0.173608	-3.266402
63	6	-5.603674	-4.328816	-1.962354
64	6	1.222417	-1.769746	2.785488
65	6	-5.638996	-4.840400	-3.271055
66	1	-5.272298	-4.229778	-4.090344
67	6	-2.133651	5.533871	2.864304
68	1	-2.280484	5.373699	1.800434
69	6	-9.897194	3.123010	0.915424
70	1	-10.453440	3.496923	1.770533
71	6	1.521008	-3.121694	2.937246
72	1	0.785315	-3.812601	3.337309
73	6	2.381014	3.056679	-3.214186
74	6	10.397847	3.293999	0.381717
75	6	0.691124	1.136854	3.747752
76	1	1.713029	0.932120	4.032467
77	6	-3.719102	-2.659641	-2.055524
78	6	7.792642	-0.952989	0.471827
79	1	8.806476	-0.654338	0.246647
80	6	3.489409	-3.805713	-2.853763
81	1	4.347436	-4.471383	-2.821286
82	6	-3.488163	-3.821069	2.839552
83	1	-4.345498	-4.487513	2.804555
84	6	2.153985	4.442103	-3.720903
85	6	-8.684728	2.462417	1.115732
86	1	-8.305364	2.317341	2.122656
87	6	3.521055	2.717625	-2.507957
88	6	-6.095991	-5.126240	-0.915442
89	1	-6.065782	-4.741745	0.099445
90	6	-5.913293	-2.033313	-1.080691
91	6	-6.641516	-6.894133	-2.474335
92	1	-7.042033	-7.884067	-2.672659
93	6	-1.930430	4.652706	5.101875
94	1	-1.944606	3.808778	5.785592
95	6	-6.607323	-6.399303	-1.169578
96	1	-6.977615	-7.004477	-0.346967
97	6	-1.696208	7.030134	4.715788
98	1	-1.520734	8.032814	5.094950
99	6	-0.692369	1.151741	-3.744870
100	1	-1.714285	0.948028	-4.030323
101	6	2.133870	5.545635	-2.851033
102	1	2.279520	5.383514	-1.787293
103	6	4.611081	3.606159	-2.207455
104	1	4.606086	4.616348	-2.604477
105	6	5.632871	3.170409	-1.413220
106	1	6.446158	3.837025	-1.145004
107	6	5.606737	-4.333517	1.955174
108	6	-1.909228	6.821750	3.351863
109	1	-1.892944	7.661928	2.663182
110	6	7.946919	1.961520	-0.023641
111	6	1.698730	7.045507	-4.700184
112	1	1.523946	8.048950	-5.077639
113	6	-10.398729	3.295472	-0.376123
114	1	-11.342404	3.810441	-0.531843
115	6	1.910385	6.834499	-3.336453
116	1	1.893727	7.673403	-2.646234
117	6	-1.706320	5.940338	5.589015

118	1	-1.543381	6.092622	6.652365
119	6	-1.427018	-3.005981	1.873886
120	1	-0.689223	-3.029070	1.078927
121	6	-1.222707	-1.758921	-2.790704
122	6	6.652982	1.237598	-0.222628
123	6	9.681647	2.793148	1.471196
124	6	-2.801394	-3.567415	-2.583487
125	1	-3.068370	-4.611614	-2.709756
126	6	-1.519693	-3.111089	-2.944127
127	1	-0.783115	-3.800605	-3.344970
128	6	1.932478	4.668736	-5.090431
129	1	1.946970	3.826056	-5.775690
130	6	6.647719	-6.897975	2.465329
131	1	7.049451	-7.887557	2.662944
132	6	6.606365	-0.114173	0.312963
133	6	8.682480	2.464819	-1.110705
134	6	1.709292	5.957334	-5.575418
135	1	1.547389	6.111618	-6.638637
136	6	7.355608	-2.159345	0.918849
137	1	7.945901	-3.032134	1.157621
138	6	9.895250	3.124675	-0.909829
139	6	8.472138	2.128981	1.269980
140	6	6.100763	-5.129292	0.907829
141	1	6.070878	-4.743880	-0.106711
142	6	6.613660	-6.401916	1.161046
143	1	6.985294	-7.005759	0.338065
144	6	6.163030	-6.114779	3.515608
145	1	6.194291	-6.490168	4.534467
146	6	5.642044	-4.846318	3.263424
147	1	5.274169	-4.237004	4.083159
148	1	11.341749	3.808414	0.537887
149	1	10.064381	2.920651	2.479966
150	1	7.917927	1.742682	2.120475
151	1	8.302187	2.322336	-2.117647
152	1	10.450868	3.500515	-1.764502

Coordinates for **$CH_3CN\supset$3-NN**

Center Number	Symbol	Coordinates (Angstroms) X	Y	Z
------	-----------	--------------	------------	-----------
1	C	3.4907730	2.8231460	1.7467400
2	C	2.4981540	3.7135900	2.1547440
3	H	2.6855270	4.7833880	2.1594840
4	C	1.2528450	3.2017160	2.5493530
5	H	0.4537320	3.8646440	2.8684070
6	C	1.0750890	1.8183160	2.5455420
7	C	-0.1703680	1.1731600	2.8394980
8	C	-0.2788330	-0.1806820	3.1569370
9	C	0.7406470	-1.1212810	3.6015020
10	H	1.7506480	-0.8756730	3.9001090
11	C	0.1409600	-2.3450130	3.6578630
12	H	0.5804600	-3.2892390	3.9500160
13	C	-1.2357970	-2.1461510	3.2300400
14	C	-2.2124870	-3.1353010	2.8890660
15	C	-3.3487550	-2.7400130	2.2017770
16	C	-4.3795410	-3.6130480	1.7270830
17	H	-4.3295650	-4.6741370	1.9526240
18	C	-5.3950920	-3.0966630	0.9724690
19	H	-6.1621310	-3.7430730	0.5551330
20	C	-5.4104220	-1.6873070	0.7092050
21	C	-6.4473340	-1.0284400	0.0832260
22	C	-6.3749480	0.3736740	-0.2760130
23	C	-7.5237740	1.2688350	-0.3230910
24	H	-8.5472300	0.9809770	-0.1250880
25	C	-7.0389740	2.5015500	-0.6357390
26	H	-7.5908080	3.4213940	-0.7717610
27	C	-5.6086580	2.3362600	-0.8177140
28	C	-4.7096710	3.2685050	-1.3228160
29	C	-3.3893520	2.8981580	-1.7239060
30	C	-2.3831190	3.7780420	-2.1197140
31	H	-2.5519850	4.8508680	-2.1073220
32	C	-1.1473300	3.2508520	-2.5256260
33	H	-0.3374130	3.9050520	-2.8354820
34	C	-0.9935690	1.8654360	-2.5445510
35	C	0.2416780	1.2035050	-2.8492080
36	C	0.3218290	-0.1479480	-3.1794370
37	C	-0.7199210	-1.0646020	-3.6178490
38	H	-1.7249620	-0.7970900	-3.9131300
39	C	-0.1572150	-2.3041470	-3.6586150
40	H	-0.6304890	-3.2415620	-3.9145960
41	C	1.2226530	-2.1396650	-3.2306700
42	C	2.1637170	-3.1538200	-2.8623570
43	C	3.3114230	-2.7855990	-2.1797500
44	C	4.3191810	-3.6838730	-1.6998080
45	H	4.2383660	-4.7451240	-1.9178820
46	C	5.3561360	-3.1884070	-0.9594850
47	H	6.1107200	-3.8515120	-0.5450360
48	C	5.4121640	-1.7773390	-0.7093060
49	C	6.4678980	-1.1425480	-0.0896410
50	C	6.4266210	0.2602640	0.2722630
51	C	7.5911730	1.1336990	0.3205730
52	H	8.6083860	0.8291140	0.1154630
53	C	7.1302580	2.3728250	0.6459760
54	H	7.6995690	3.2812740	0.7865490
55	C	5.6983490	2.2325180	0.8325320
56	C	4.8162550	3.1754330	1.3491320
57	Te	2.7952370	0.8622310	1.7820820
58	N	-1.4719330	-0.8524680	2.9974140
59	Te	-3.7062890	-0.7524790	1.6243980
60	N	-5.2473690	1.0265270	-0.5720050
61	Te	-2.7309190	0.9252090	-1.7966780
62	N	1.5003990	-0.8505530	-3.0136070
63	Te	3.7325090	-0.8009590	-1.6324570
64	N	5.3124740	0.9320360	0.5776650
65	C	-1.4217650	1.9602450	2.7510950
66	C	-1.7160210	2.7564190	1.6334120
67	H	-1.0265060	2.7837510	0.7945610
68	C	-2.9018440	3.4838060	1.5816630
69	H	-3.1187560	4.0910130	0.7104710
70	C	-3.8099020	3.4214620	2.6367070
71	H	-4.7358910	3.9876570	2.5932890
72	C	-3.5287370	2.6319230	3.7517500
73	H	-4.2343130	2.5771480	4.5756170
74	C	-2.3446370	1.9080180	3.8081670
75	H	-2.1234540	1.2834660	4.6672520
76	C	-1.9140520	-4.5563540	3.1896860
77	C	-1.6621900	-4.9567080	4.5102610
78	H	-1.7166970	-4.2182260	5.3059310
79	C	-1.3576360	-6.2836980	4.8033130
80	H	-1.1716740	-6.5802210	5.8321360
81	C	-1.2973330	-7.2296530	3.7797520
82	H	-1.0598100	-8.2651800	4.0079060
83	C	-1.5373540	-6.8385650	2.4626050
84	H	-1.4780320	-7.5667180	1.6580850
85	C	-1.8406640	-5.5118620	2.1650760
86	H	-1.9897460	-5.1958740	1.1366040
87	C	-7.7375890	-1.7018140	-0.2079420
88	C	-8.3141740	-1.6001990	-1.4841710
89	H	-7.7922920	-1.0407660	-2.2556960
90	C	-9.5296140	-2.2164830	-1.7668940
91	H	-9.9555330	-2.1364970	-2.7632970
92	C	-10.1971400	-2.9374220	-0.7762870
93	H	-11.1471260	-3.4164840	-0.9963330
94	C	-9.6420510	-3.0349420	0.4995110
95	H	-10.1626220	-3.5830610	1.2800500
96	C	-8.4247840	-2.4210180	0.7822720
97	H	-7.9998860	-2.4789570	1.7802050
98	C	-5.1402930	4.6798060	-1.4568200
99	C	-5.6572750	5.3623230	-0.3456590
100	H	-5.7283350	4.8395310	0.6030850
101	C	-6.0658500	6.6889940	-0.4550550
102	H	-6.4562670	7.2062100	0.4168640
103	C	-5.9698470	7.3512920	-1.6781810
104	H	-6.2892130	8.3859910	-1.7645760
105	C	-5.4630630	6.6799190	-2.7917600
106	H	-5.3960210	7.1876870	-3.7498200
107	C	-5.0471860	5.3564250	-2.6826820
108	H	-4.6609900	4.8283800	-3.5495080
109	C	1.5078320	1.9646120	-2.7488350
110	C	1.8143110	2.7450230	-1.6232530
111	H	1.1214120	2.7806360	-0.7876160

112	C	3.0162420	3.4442570	-1.5590950
113	H	3.2422170	4.0391720	-0.6816710
114	C	3.9288310	3.3691370	-2.6095300
115	H	4.8677350	3.9128070	-2.5563120
116	C	3.6356110	2.5954720	-3.7325180
117	H	4.3442730	2.5309390	-4.5530300
118	C	2.4350840	1.8999770	-3.8014160
119	H	2.2040000	1.2881320	-4.6670300
120	C	1.8152150	-4.5702040	-3.1259130
121	C	1.5701180	-5.0066890	-4.4354670
122	H	1.6662950	-4.2990270	-5.2546120
123	C	1.2163570	-6.3298950	-4.6869360
124	H	1.0346860	-6.6549460	-5.7077660
125	C	1.0979840	-7.2354240	-3.6325970
126	H	0.8187210	-8.2669270	-3.8285750
127	C	1.3298100	-6.8087820	-2.3253570
128	H	1.2205940	-7.5033300	-1.4970790
129	C	1.6825930	-5.4860030	-2.0729320
130	H	1.8314150	-5.1425510	-1.0537070
131	C	7.7430650	-1.8458360	0.1963440
132	C	8.4036020	-2.5889520	-0.7945630
133	H	7.9695860	-2.6417210	-1.7888710
134	C	9.6068230	-3.2324240	-0.5175410
135	H	10.1068510	-3.7983970	-1.2987590
136	C	10.1748150	-3.1411480	0.7530550
137	H	11.1140840	-3.6428830	0.9684700
138	C	9.5343790	-2.3966320	1.7441260
139	H	9.9708100	-2.3206980	2.7362990
140	C	8.3328790	-1.7510640	1.4671860
141	H	7.8327000	-1.1725400	2.2390400
142	C	5.2712040	4.5775070	1.4977050
143	C	5.7987010	5.2630580	0.3933800
144	H	5.8594420	4.7497970	-0.5612460
145	C	6.2300310	6.5812420	0.5167720
146	H	6.6281980	7.1012340	-0.3499630
147	C	6.1467000	7.2316910	1.7471840
148	H	6.4838250	8.2597640	1.8444230
149	C	5.6296220	6.5570390	2.8540000
150	H	5.5721940	7.0554380	3.8175810
151	C	5.1910130	5.2421230	2.7310070
152	H	4.7968810	4.7113020	3.5925570
153	C	-0.0449870	-1.5815710	0.0529660
154	H	0.6357010	-1.2454570	-0.7321980
155	H	0.5377680	-1.8215890	0.9459210
156	H	-0.7403260	-0.7769010	0.3051990
157	C	-0.7839850	-2.7548190	-0.3782990
158	N	-1.3692350	-3.6992590	-0.7138210

Coordinates for **3-Cl$_2$-a**

Center Number	Atomic Number	Coordinates (Angstroms) X	Y	Z
------	-----------	--------------	------------	-----------
1	52	21.601207	12.017230	6.425665
2	52	19.787159	11.894307	2.851784
3	17	22.153544	13.842534	4.656701
4	17	21.461781	10.300894	8.364858
5	7	20.122775	13.783432	7.947710
6	6	16.547365	14.945362	7.633868
7	6	18.166293	11.531043	7.970629
8	1	19.025817	11.698662	8.610444
9	6	22.914354	13.177684	7.637016
10	6	17.403513	12.592473	7.493028
11	7	21.359885	9.708002	4.021000
12	6	17.666164	13.979918	7.735273
13	6	18.932025	14.476000	8.068856
14	6	24.255038	12.724005	7.446513
15	1	25.056029	13.214598	7.990829
16	6	20.519451	15.821202	8.941911
17	1	21.077081	16.623882	9.403851
18	6	21.069282	14.578998	8.445387
19	6	17.784896	10.230698	1.583808
20	1	17.227563	9.378068	1.210167
21	6	22.474833	14.233796	8.380623
22	6	19.454307	8.796058	2.768822
23	6	15.331689	14.708851	8.298002
24	1	15.221419	13.816521	8.906745
25	6	15.632312	17.023932	6.774000
26	1	15.744461	17.915916	6.164323
27	6	24.273397	16.070850	11.162890
28	1	24.212359	16.165707	12.243547
29	6	23.438609	11.028478	5.916344
30	6	20.611360	8.647744	3.527352
31	6	22.445859	9.156646	4.565239
32	6	18.911841	10.064106	2.391318
33	6	23.443663	15.106814	9.097660
34	6	14.288591	15.625216	8.209969
35	1	13.358592	15.433248	8.736752
36	6	14.434058	16.781011	7.444297
37	1	13.612381	17.486781	7.363542
38	6	18.771859	7.568220	2.266738
39	6	19.183565	15.767294	8.681481
40	1	18.430790	16.502375	8.929121
41	6	24.429190	15.811041	8.391948
42	1	24.471946	15.720626	7.309975
43	6	23.542370	9.858148	5.219799
44	6	24.516096	11.702356	6.582100
45	1	25.535862	11.377233	6.402351
46	6	22.424299	7.705539	4.459148
47	1	23.182883	7.037522	4.842359
48	6	16.683855	16.118514	6.872088
49	1	17.612951	16.295720	6.338678
50	6	25.253310	16.765189	10.453529
51	1	25.954881	17.406885	10.979315
52	6	17.471716	7.248515	2.682199
53	1	16.958034	7.893768	3.388194
54	6	19.417721	6.724962	1.351257
55	1	20.417492	6.977362	1.009633
56	6	17.489663	5.270461	1.294111
57	1	16.991463	4.382397	0.915493
58	6	24.855651	9.148215	5.173514
59	6	23.370849	15.250470	10.490452
60	1	22.610993	14.705248	11.043532
61	6	21.259218	7.387650	3.839199
62	1	20.876511	6.404852	3.603348
63	6	25.326882	16.635396	9.067340
64	1	26.081438	17.181255	8.507618
65	6	25.488382	8.725899	6.351398
66	1	25.010950	8.921100	7.307778
67	6	25.458264	8.863499	3.939894
68	1	24.970677	9.183280	3.023040
69	6	16.839030	6.104583	2.202208
70	1	15.832642	5.872651	2.537725
71	6	26.676780	8.191315	3.887087
72	1	27.138624	7.988139	2.924807
73	6	26.702172	8.043704	6.295117
74	1	27.177312	7.714764	7.215261
75	6	27.301367	7.778991	5.064198
76	1	28.249293	7.249723	5.021728
77	6	18.779253	5.584782	0.868288
78	1	19.289632	4.945341	0.153334
79	52	13.951998	12.017198	2.791520
80	52	15.766070	11.894341	6.365595
81	17	13.399670	13.842671	4.560316
82	17	14.091324	10.300704	0.852462
83	7	15.430474	13.783370	1.269528
84	6	19.005838	14.945382	1.583628
85	6	17.387030	11.531047	1.246599
86	1	16.527550	11.698682	0.606730
87	6	12.638884	13.177600	1.580102
88	6	18.149745	12.592467	1.724321
89	7	14.193298	9.708078	5.196346
90	6	17.887070	13.979914	1.482126
91	6	16.621216	14.475967	1.148464
92	6	11.298193	12.723944	1.770617
93	1	10.497212	13.214516	1.226267
94	6	15.033816	15.821126	0.275295
95	1	14.476199	16.623793	-0.186683
96	6	14.483980	14.578908	0.771778
97	6	17.768416	10.230703	7.633393
98	1	18.325782	9.378062	8.006962
99	6	13.078429	14.233683	0.836468

100	6	16.098907	8.796098	6.448468
101	6	20.221572	14.708906	0.919591
102	1	20.331911	13.816585	0.310846
103	6	19.920779	17.023959	2.443602
104	1	19.808561	17.915931	3.053283
105	6	11.279962	16.070672	-1.945904
106	1	11.341041	16.165507	-3.026560
107	6	12.114583	11.028530	3.300935
108	6	14.941820	8.647808	5.689983
109	6	13.107303	9.156738	4.652129
110	6	16.641409	10.064132	6.825965
111	6	12.109623	15.106684	0.119379
112	6	21.264645	15.625290	1.007723
113	1	22.194692	15.433347	0.481015
114	6	21.119092	16.781071	1.773399
115	1	21.940748	17.486857	1.854232
116	6	16.781362	7.568243	6.950499
117	6	16.369684	15.767257	0.535830
118	1	17.122456	16.502362	0.288251
119	6	11.124065	15.810920	0.825037
120	1	11.081266	15.720526	1.907011
121	6	12.010802	9.858246	3.997554
122	6	11.037113	11.702366	2.635109
123	1	10.017341	11.377271	2.814874
124	6	13.128830	7.705634	4.758255
125	1	12.370222	7.037626	4.375075
126	6	18.869262	16.118520	2.345416
127	1	17.940120	16.295698	2.878755
128	6	10.300019	16.765021	-1.236595
129	1	9.598465	17.406703	-1.762420
130	6	18.081492	7.248541	6.534991
131	1	18.595158	7.893810	5.828998
132	6	16.135523	6.724965	7.865977
133	1	15.135763	6.977362	8.207637
134	6	18.063573	5.270450	7.923027
135	1	18.561778	4.382373	8.301607
136	6	10.697507	9.148342	4.043873
137	6	12.182488	15.250310	-1.273414
138	1	12.942368	14.705080	-1.826453
139	6	14.293923	7.387727	5.378172
140	1	14.676616	6.404923	5.614023
141	6	10.226395	16.635257	0.149594
142	1	9.471813	17.181123	0.709276
143	6	10.064756	8.726007	2.866007
144	1	10.542182	8.921171	1.909617
145	6	10.094897	8.863678	5.277506
146	1	10.582498	9.183476	6.194347
147	6	18.714184	6.104592	7.014933
148	1	19.720561	5.872663	6.679381
149	6	8.876367	8.191523	5.330343
150	1	8.414526	7.988387	6.292633
151	6	8.850950	8.043841	2.922318
152	1	8.375796	7.714887	2.002187
153	6	8.251760	7.779178	4.153250
154	1	7.303823	7.249934	4.195744
155	6	16.773997	5.584768	8.348897
156	1	16.263634	4.945312	9.063848

Coordinates for **3-Cl_2-p**

Center Number	Atomic Number	Coordinates (Angstroms) X	Y	Z
------	-----------	----------------	-------------	-----------
1	Te	-4.2540130	-0.0295460	0.0369340
2	Te	-0.9827290	-0.0102020	2.4464330
3	Te	-1.3350490	0.0957310	-2.6342960
4	Te	4.6101180	-0.0745860	0.0675420
5	Cl	5.0173710	1.7753620	1.8843180
6	Cl	4.6293800	-1.9657290	-1.7545180
7	Cl	-4.2486980	-1.7901510	1.9661340
8	Cl	-4.6484220	1.7019660	-1.8891190
9	N	-2.9817200	2.2779730	1.9318840
10	N	3.5256690	1.7030450	-2.0595760
11	N	3.7639570	-1.8131610	2.1624180
12	N	-3.4918230	-2.1956270	-1.9091590
13	C	-0.5006620	-1.8039920	-2.3840040
14	C	0.6891640	0.6691430	-2.4952160
15	C	-4.1667590	2.8942260	1.9111470
16	C	1.3574700	1.6947520	2.4858400
17	H	2.0205550	2.5519850	2.4966710
18	C	-5.9796140	-1.0687600	-0.6619360
19	C	2.4407850	2.4810690	-2.4568840
20	C	-0.0198090	1.8407940	2.3026030
21	C	-6.6232030	3.0478660	2.3362030
22	C	0.2159950	4.3276170	2.1391330
23	C	-2.0324740	3.2998680	1.9837710
24	C	-5.9360860	-2.1480130	-1.4964030
25	C	-6.6787090	3.3277460	3.7109390
26	H	-5.8966440	2.9505940	4.3634140
27	C	0.8892640	-1.7132590	-2.2594980
28	H	1.5030280	-2.5963310	-2.1198330
29	C	1.4969470	-0.4525320	-2.3223790
30	H	2.5724890	-0.3558720	-2.2824060
31	C	0.0987830	3.0347280	-3.0555540
32	C	7.0829770	2.8703450	-2.2728340
33	C	-2.6922060	4.5952920	1.9476810
34	H	-2.1979010	5.5551370	1.9454410
35	C	-0.6604770	3.1095210	2.1267680
36	C	-4.0266980	4.3442640	1.9436170
37	H	-4.8400670	5.0547340	1.9216680
38	C	1.0132750	-0.6754160	2.6560780
39	C	-0.4194090	-4.2886070	-2.6498770
40	C	1.1294370	2.0310550	-2.6335980
41	C	-7.0105090	0.5037200	0.8735000
42	H	-7.8771170	0.8993410	1.3911480
43	C	-5.4756620	2.2591800	1.7824090
44	C	1.3949130	-2.0483310	2.8449300
45	C	-7.6347110	3.5468130	1.5001400
46	H	-7.5840550	3.3553340	0.4323680
47	C	2.6877620	-2.5404810	2.6636770
48	C	6.1637500	0.9526480	-0.9882000
49	C	-1.2168760	-3.0438560	-2.4354630
50	C	1.8859910	0.4091540	2.6663420
51	H	2.9444870	0.2708130	2.8411990
52	C	-5.7207700	1.0924000	1.1182520
53	C	-8.6834810	4.2995020	2.0306520
54	H	-9.4551800	4.6857360	1.3709210
55	C	0.3344500	-3.0054020	3.2952280
56	C	4.2486740	3.8360860	-2.5762380
57	H	4.9370970	4.6593850	-2.7018230
58	C	5.9394030	2.0478560	-1.7785120
59	C	-7.2153840	-2.8409910	-1.8588500
60	C	0.1792210	5.2211010	3.2206150
61	H	-0.4892510	5.0231100	4.0531040
62	C	4.5980260	2.4903240	-2.1367820
63	C	2.8997400	3.8376030	-2.7457360
64	H	2.2736460	4.6616110	-3.0550460
65	C	7.2480130	3.1061210	-3.6480940
66	H	6.5449990	2.6698870	-4.3516070
67	C	-7.7334800	4.0711690	4.2398770
68	H	-7.7695910	4.2700510	5.3070910
69	C	0.4336070	-4.4199100	-3.7598920
70	H	0.5124150	-3.6038050	-4.4710250
71	C	-8.7380530	4.5596190	3.4012900
72	H	-9.5563560	5.1429640	3.8134270
73	C	7.9997140	3.4399690	-1.3735010
74	H	7.8623890	3.2842450	-0.3076390
75	C	6.2731510	-1.1557870	0.8643610
76	C	4.8218930	-2.6199420	2.2167020
77	C	-4.7094950	-2.7478550	-1.9961880
78	C	1.0972990	4.5837550	1.0774040
79	H	1.1430070	3.8980790	0.2374150
80	C	-0.9194050	3.4278530	-2.1720330
81	H	-0.9437920	3.0301600	-1.1622630
82	C	1.8687500	6.5994240	2.1708970
83	H	2.5100280	7.4758280	2.1846800
84	C	1.0011420	6.3488890	3.2352680
85	H	0.9657580	7.0279850	4.0822890
86	C	-7.1409260	-0.5063130	-0.0312570
87	H	-8.1157640	-0.9168070	-0.2698180
88	C	6.1260870	-2.2300520	1.6993430
89	C	7.4805930	-0.6293970	0.3000810

90	H	8.4332960	-1.0476500	0.6075950
91	C	0.1185810	3.5748070	-4.3522440
92	H	0.8980860	3.2698080	-5.0439270
93	C	9.0591740	4.2185300	-1.8403730
94	H	9.7549310	4.6592490	-1.1321190
95	C	-2.6037160	-3.1815770	-2.3278160
96	C	7.4265620	0.3834360	-0.6173980
97	H	8.3345280	0.7663270	-1.0714310
98	C	3.1370880	-3.8835170	3.0112020
99	H	2.5133670	-4.6719670	3.4062850
100	C	7.2985450	-3.0762290	2.0664310
101	C	-1.8964340	4.3396040	-2.5766660
102	H	-2.6789770	4.6240130	-1.8810510
103	C	1.9140670	5.7141480	1.0922960
104	H	2.5894010	5.8937440	0.2612360
105	C	4.4744500	-3.9237660	2.7650700
106	H	5.1511100	-4.7554760	2.8987190
107	C	9.2206200	4.4369230	-3.2099270
108	H	10.0470470	5.0415190	-3.5722980
109	C	8.3123310	3.8783460	-4.1122430
110	H	8.4329820	4.0425470	-5.1792140
111	C	-7.9549700	-3.5407170	-0.8934220
112	H	-7.5927770	-3.5774380	0.1299570
113	C	-0.8596910	4.4842820	-4.7533090
114	H	-0.8357310	4.8885760	-5.7612300
115	C	-0.5116760	-5.3557620	-1.7394600
116	H	-1.1541660	-5.2580440	-0.8701190
117	C	-1.8693490	4.8678450	-3.8672050
118	H	-2.6337570	5.5712440	-4.1835500
119	C	-0.6671850	-3.4321700	2.4103230
120	H	-0.6556290	-3.0951220	1.3780810
121	C	-0.6893750	-4.3341800	5.0513270
122	H	-0.6958660	-4.6792560	6.0813090
123	C	-7.6784560	-2.8126130	-3.1830920
124	H	-7.1102390	-2.2733600	-3.9354350
125	C	1.1615410	-5.5910650	-3.9595840
126	H	1.8080500	-5.6801230	-4.8274460
127	C	0.3141160	-3.4661130	4.6214030
128	H	1.0821420	-3.1327980	5.3129730
129	C	-1.6861240	-4.7495240	4.1656210
130	H	-2.4728750	-5.4170270	4.5042100
131	C	-1.6732050	-4.2966530	2.8463930
132	H	-2.4525220	-4.6005820	2.1553410
133	C	-4.6537060	-4.1004940	-2.5237860
134	H	-5.5014920	-4.7447080	-2.7037410
135	C	-3.3357620	-4.3797020	-2.7047200
136	H	-2.8991580	-5.2864960	-3.0956920
137	C	1.0649450	-6.6430880	-3.0456230
138	H	1.6415250	-7.5508570	-3.1972980
139	C	0.2303130	-6.5205580	-1.9335740
140	H	0.1586320	-7.3297240	-1.2127360
141	C	-9.1370610	-4.1942150	-1.2457580
142	H	-9.6975410	-4.7365070	-0.4896220
143	C	-8.8631390	-3.4621270	-3.5313200
144	H	-9.2145700	-3.4257320	-4.5584630
145	C	-9.5948230	-4.1547860	-2.5641190
146	H	-10.5156260	-4.6621390	-2.8370240
147	C	9.4930210	-4.6856270	2.7693590
148	H	10.3415480	-5.3068240	3.0412280
149	C	7.6286690	-3.2853970	3.4159420
150	H	7.0324580	-2.8114780	4.1901400
151	C	8.0783870	-3.6938980	1.0746890
152	H	7.8115960	-3.5594620	0.0307350
153	C	8.7210790	-4.0794790	3.7632350
154	H	8.9703640	-4.2232080	4.8106630
155	C	9.1666110	-4.4933960	1.4255630
156	H	9.7552770	-4.9711600	0.6476580

Coordinates for **4**

Center Number	Symbol	Coordinates (Angstroms) X	Y	Z
------	-----------	----------------	-------------	-----------
1	Te	5.8533730	5.0382480	5.8395270
2	Te	1.2984610	5.7398810	5.8445720
3	Te	6.2009250	7.6921960	8.7612090
4	Te	1.4795110	6.6410430	1.9922710
5	Ru	7.7988770	6.5852610	6.8610010
6	Ru	-0.2109210	7.0741720	4.0671980
7	O	7.7804530	8.9656080	4.9892590
8	O	0.6245430	9.9450290	4.5619290
9	O	10.2868370	5.5654800	5.4895290
10	N	8.0525810	5.0187250	8.2919670
11	N	4.1702840	8.4543500	8.1995910
12	N	-0.9966070	5.1589450	3.5443540
13	N	3.6584750	6.9844110	2.3309940
14	C	7.7341060	8.0620130	5.7072840
15	C	4.0022470	8.2981090	2.1817950
16	C	4.7973970	4.8464150	2.9083870
17	C	1.7184710	8.7079270	1.5465800
18	C	5.5017650	4.2049600	3.9006440
19	C	2.9601040	6.3444940	9.9405820
20	C	3.3128150	10.6371700	1.4815020
21	C	2.4486040	6.6997390	8.5909210
22	C	-0.0670880	4.2109530	6.3541890
23	C	6.4655620	2.3071450	5.0751340
24	H	6.8063900	1.2769480	5.0964320
25	C	0.0036670	4.0013640	7.7730470
26	H	-0.6110600	3.2276480	8.2218510
27	O	-2.7730330	7.5838590	5.5810400
28	C	6.6990490	3.1415000	6.2201810
29	C	7.7431800	1.3315940	7.5102440
30	C	1.5337240	5.8821070	7.9686260
31	C	4.1002220	4.0526830	1.8632290
32	C	5.3540840	8.4590680	2.5281660
33	H	5.9254110	9.3742520	2.4662200
34	C	4.7483350	6.2887110	2.7913090
35	C	3.9499520	5.7008540	12.4919720
36	H	4.3315260	5.4524420	13.4784840
37	C	3.8997400	4.7275070	11.4947810
38	H	4.2521780	3.7199210	11.6971530
39	C	2.1471150	8.9172830	7.3337850
40	H	1.0884420	8.8398990	7.1282570
41	C	3.4068210	5.0450990	10.2310960
42	H	3.3824560	4.2904640	9.4503040
43	C	0.7676560	4.8224330	8.5523170
44	H	0.7704510	4.7145210	9.6335520
45	C	5.8987420	2.8303770	3.9479520
46	H	5.7813140	2.2243860	3.0536730
47	C	3.5098130	6.9965680	12.2158650
48	H	3.5405000	7.7587070	12.9897630
49	C	-0.7941820	7.4018010	2.0195650
50	C	2.9791440	9.2109930	1.7188870
51	C	7.4107260	2.7813800	7.3459040
52	C	-1.5870850	6.3410520	1.5387220
53	C	2.9092870	7.9447700	8.0122700
54	C	5.5043490	10.3879220	7.6648270
55	C	0.3555400	8.8392070	4.3721390
56	C	4.2256990	4.4044610	0.5085220
57	H	4.8495240	5.2515020	0.2384670
58	C	3.2896050	2.9563690	2.1997950
59	H	3.1653050	2.6909710	3.2457430
60	C	3.0272220	7.3183710	10.9513520
61	H	2.6858680	8.3266660	10.7367110
62	C	6.5856390	9.6857570	8.1230730
63	C	0.4784830	9.3590600	1.2285840
64	H	0.5084930	10.3864970	0.8758150
65	C	9.0960330	6.7968200	9.5232670
66	C	3.0712340	11.2307410	0.2345650
67	H	2.6548500	10.6279330	-0.5679440
68	C	-3.0596250	2.3834880	6.1349650
69	H	-3.6124030	3.2700460	5.8375500
70	C	-1.6250920	5.1587420	2.3151210
71	C	4.2279260	9.7032590	7.6493090
72	C	9.3233690	5.9530990	5.9981540
73	C	2.9714790	10.0246860	7.1092680
74	H	2.6978930	10.9530910	6.6283830
75	C	-1.6623940	2.3757980	6.0149610
76	C	5.8157380	7.2009670	2.9258740
77	H	6.8297900	6.9297730	3.1860160
78	C	-0.9293360	3.5697560	5.4892260
79	C	9.0414810	4.3134910	10.2320080
80	H	9.5716380	4.3424300	11.1737960

81	C	7.9705370	10.0452030	8.2403890
82	H	8.2481190	11.0898470	8.1226760
83	C	8.7481230	5.4320020	9.4107930
84	C	3.8772870	11.4157200	2.5025950
85	H	4.0588940	10.9697920	3.4758620
86	C	8.5307790	3.2122910	9.5821430
87	H	8.5533240	2.1851990	9.9172120
88	C	-0.7149590	8.7272710	1.4041790
89	H	-1.6299680	9.2449400	1.1244120
90	C	-0.9609210	1.2268520	6.4058020
91	H	0.1215260	1.2113830	6.3109710
92	C	3.9366730	13.3380250	1.0387100
93	H	4.1775580	14.3836490	0.8673830
94	C	8.3540510	-1.3947700	7.8036230
95	H	8.5909150	-2.4490130	7.9176250
96	C	-1.6374120	0.1133490	6.9003010
97	H	-1.0783080	-0.7720310	7.1908940
98	C	8.5862490	7.6777420	8.5460260
99	C	-1.2634950	3.9625630	4.1420490
100	C	4.1833460	12.7564430	2.2819720
101	H	4.6117620	13.3454440	3.0880470
102	C	6.7238130	0.3831390	7.6721150
103	H	5.6897210	0.7167830	7.6881220
104	C	-2.2704670	3.9136490	2.1083730
105	H	-2.8359320	3.6320420	1.2309860
106	C	-2.0398350	3.1657140	3.2421230
107	H	-2.3686010	2.1550400	3.4375890
108	C	3.5742320	3.6709740	-0.4778750
109	H	3.6948580	3.9475050	-1.5217460
110	C	7.0266510	-0.9689360	7.8211380
111	H	6.2242930	-1.6897020	7.9539110
112	C	8.9224350	9.1009910	8.4798800
113	H	9.9552470	9.4160430	8.6114630
114	C	7.9376680	3.6614030	8.3589980
115	C	9.0747290	0.8918430	7.4973670
116	H	9.8719490	1.6185130	7.3691330
117	C	3.3809330	12.5710650	0.0155840
118	H	3.1933720	13.0145870	-0.9586380
119	C	2.6290490	2.2302780	1.2112570
120	H	1.9958040	1.3930420	1.4912000
121	C	-3.7348500	1.2730270	6.6360770
122	H	-4.8170970	1.2988090	6.7301410
123	C	-3.0263740	0.1339140	7.0183130
124	H	-3.5544840	-0.7329890	7.4057680
125	C	2.7717710	2.5827880	-0.1303160
126	H	2.2592980	2.0141260	-0.9013570
127	C	-1.7821340	7.3912470	5.0168600
128	C	9.3766690	-0.4605620	7.6392190
129	H	10.4134410	-0.7852830	7.6189450
130	C	5.7646960	14.4544210	6.3125300
131	H	5.8324420	15.4862520	5.9787560
132	C	4.8052630	14.0838870	7.2549950
133	H	4.1237940	14.8269890	7.6601580
134	C	5.5957860	11.7875550	7.1837040
135	C	4.7188650	12.7627940	7.6849580
136	H	3.9771290	12.4784440	8.4257030
137	C	6.5516720	12.1693110	6.2305370
138	H	7.2128750	11.4177930	5.8108750
139	C	6.6348380	13.4924780	5.8008480
140	H	7.3800160	13.7697350	5.0599970
141	C	9.9275060	7.2483500	10.6692780
142	C	11.2141650	7.7646600	10.4620180
143	H	11.6135730	7.8134160	9.4525920
144	C	11.9901760	8.1850760	11.5391840
145	H	12.9893870	8.5739550	11.3630590
146	C	9.4369130	7.1617410	11.9797210
147	H	8.4393740	6.7655540	12.1485040
148	C	10.2119120	7.5917330	13.0551580
149	H	9.8145960	7.5265200	14.0644460
150	C	11.4900340	8.1037950	12.8383970
151	H	12.0943870	8.4367480	13.6775670
152	C	-2.3291690	6.3995600	0.2529010
153	C	-4.0808700	7.3514230	-1.1306860
154	H	-4.9058130	8.0487270	-1.2486150
155	C	-3.3877700	7.3012280	0.0760470
156	H	-3.6822040	7.9470870	0.8986690
157	C	-3.7216100	6.5085810	-2.1819680
158	H	-4.2598010	6.5522120	-3.1247200
159	C	-2.6700340	5.6087070	-2.0166050
160	H	-2.3829270	4.9498440	-2.8316310
161	C	-1.9814350	5.5496100	-0.8065800
162	H	-1.1596550	4.8504940	-0.6787040

References

S1 E. Pacholska, L. Latos-Grażyński and Z. Ciunik, *Angew. Chem. Int. Ed.* **2001**, 40, 4466–4469

S2 M. J. Frisch, G. W. Trucks, H. B. Schlegel, G. E. Scuseria, M. A. Robb, J. R. Cheeseman, G. Scalmani, V. Barone, G. A. Petersson, H. Nakatsuji, X. Li, M. Caricato, A. V. Marenich, J. Bloino, B. G. Janesko, R. Gomperts, B. Mennucci, H. P. Hratchian, J. V. Ortiz, A. F. Izmaylov, J. L. Sonnenberg, D. Williams-Young, F. Ding, F. Lipparini, F. Egidi, J. Goings, B. Peng, A. Petrone, T. Henderson, D. Ranasinghe, V. G. Zakrzewski, J. Gao, N. Rega, G. Zheng, W. Liang, M. Hada, M. Ehara, K. Toyota, R. Fukuda, J. Hasegawa, M. Ishida, T. Nakajima, Y. Honda, O. Kitao, H. Nakai, T. Vreven, K. Throssell, J. A. Montgomery, Jr., J. E. Peralta, F. Ogliaro, M. J. Bearpark, J. J. Heyd, E. N. Brothers, K. N. Kudin, V. N. Staroverov, T. A. Keith, R. Kobayashi, J. Normand, K. Raghavachari, A. P. Rendell, J. C. Burant, S. S. Iyengar, J. Tomasi, M. Cossi, J. M. Millam, M. Klene, C. Adamo, R. Cammi, J. W. Ochterski, R. L. Martin, K. Morokuma, O. Farkas, J. B. Foresman, and D. J. Fox, *Gaussian, Inc.*, Wallingford CT, 2016

S3 G. Scalmani and M. J. Frisch, *J. Chem. Phys.*, **132** (2010) 114110

S4 *CrysAlis PRO*, Ver. 1.171.42.63a (Rigaku OD, 2022)

S5 *CrysAlis PRO* Ver. 1.171.39.46 (Rigaku OD, 2018)

S6 Sheldrick, G. M. SHELXT − Integrated Space-Group and Crystal-Structure Determination. *Acta Crystallogr., Sect. A*: Found. Adv. **2015**, A71, 3−8.

S7 Sheldrick, G. M. Crystal Structure Refinement with SHELXL. *Acta Crystallogr., Sect. C: Struct. Chem.* **2015**, I71, 3−8.
